# A Clinical Practice Guideline for the Emergency Management of Anaphylaxis (2020)

**DOI:** 10.3389/fphar.2022.845689

**Published:** 2022-03-28

**Authors:** Xiaotong Li, Qingbian Ma, Jia Yin, Ya’an Zheng, Rongchang Chen, Yuguo Chen, Tianzuo Li, Yuqin Wang, Kehu Yang, Hongjun Zhang, Yida Tang, Yaolong Chen, Hailong Dong, Qinglong Gu, Daihong Guo, Xuehui Hu, Lixin Xie, Baohua Li, Yuzhen Li, Tongyu Lin, Fang Liu, Zhiqiang Liu, Lanting Lyu, Quanxi Mei, Jie Shao, Huawen Xin, Fan Yang, Hui Yang, Wanhua Yang, Xu Yao, Chunshui Yu, Siyan Zhan, Guoqiang Zhang, Minggui Wang, Zhu Zhu, Baoguo Zhou, Jianqing Gu, Mo Xian, Yuan Lyu, Zhengqian Li, Hangci Zheng, Chang Cui, Shuhua Deng, Chao Huang, Lisha Li, Pengfei Liu, Peng Men, Chunli Shao, Sai Wang, Xiang Ma, Qiang Wang, Suodi Zhai

**Affiliations:** ^1^ Department of Pharmacy, Peking University Third Hospital, Beijing, China; ^2^ School of Pharmaceutical Science, Peking University, Beijing, China; ^3^ Institute for Drug Evaluation, Peking University Health Science Center, Beijing, China; ^4^ Emergency Department, Peking University Third Hospital, Beijing, China; ^5^ Department of Allergy, Peking Union Medical College Hospital, Chinese Academy of Medical Sciences and Peking Union Medical College, Beijing, China; ^6^ State Key Laboratory for Respiratory Disease, Guangzhou Institute of Respiratory Health, The First Affiliated Hospital of Guangzhou Medical University, Guangzhou, China; ^7^ Department of Emergency Medicine and Chest Pain Center, Qilu Hospital of Shandong University, Institute of Emergency and Critical Care Medicine of Shandong University, Ji’nan, China; ^8^ Department of Anesthesiology, Beijing Shijitan Hospital, Capital Medical University, Beijing, China; ^9^ Pharmacy Department, Xuanwu Hospital of Capital Medical University, Beijing, China; ^10^ Evidence-Based Medicine Center, School of Basic Medical Sciences, Lanzhou University, Lanzhou, China; ^11^ Departments of Nursing, Peking University Third Hospital, Beijing, China; ^12^ Department of Internal Medicine, Coronary Heart Disease Center, Fuwai Hospital, Chinese Academy of Medical Sciences and Peking Union Medical College, Beijing, China; ^13^ Department of Anesthesiology and Perioperative Medicine, Xijing Hospital, The Fourth Military Medical University, Xi’an, China; ^14^ Department of Otolaryngology, Children’s Hospital Affiliated to Capital Institute of Pediatrics, Beijing, China; ^15^ Pharmacy Department, Chinese PL A General Hospital, Beijing, China; ^16^ Department of Nursing, Xijing Hospital, The Fourth Military Medical University, Xi’an, China; ^17^ Department of Pulmonary and Critical Care Medicine, Chinese PLA General Hospital, Beijing, China; ^18^ Department of Pharmacy, Peking University People’s Hospital, Beijing, China; ^19^ State Key Laboratory of Oncology in South China, Department of Medical Oncology, Collaborative Innovation Center for Cancer Medicine, Sun Yat-sen University Cancer Center, Guangzhou, China; ^20^ Department of Anesthesiology, Shanghai First Maternity and Infant Hospital, Tongji University School of Medicine, Shanghai, China; ^21^ School of Public Administration and Policy, Renmin University of China, Beijing, China; ^22^ Health Technology Assessment and Health Policy Research Group at Renmin University of China, Beijing, China; ^23^ Department of Pharmacy, Shenzhen Bao’an Pure Chinese Medicine Treatment Hospital, Shenzhen, China; ^24^ Department of Pediatrics, Ruijin Hospital, Shanghai Jiaotong University School of Medicine, Shanghai, China; ^25^ Department of Clinical Pharmacology, General Hospital of Central Theater Command of PLA, Wuhan, China; ^26^ Institute of Antibiotics, Huashan Hospital Fudan University, Shanghai, China; ^27^ Departments of Nursing, The First Hospital of Shanxi Medical University, Taiyuan, China; ^28^ Department of Pharmacy, Ruijin Hospital, Shanghai Jiaotong University School of Medicine, Shanghai, China; ^29^ Institute of Dermatology, Chinese Academy of Medical Sciences and Peking Union Medical College, Nanjing, China; ^30^ Department of Radiology and Tianjin Key Laboratory of Functional Imaging, Tianjin Medical University General Hospital, Tianjin, China; ^31^ Department of Epidemiology and Biostatistics, School of Public Health, Peking University Health Science Center, Beijing, China; ^32^ Department of Emergency, China-Japan Friendship Hospital, Beijing, China; ^33^ Department of Pharmacy, Peking Union Medical College Hospital, Chinese Academy of Medical Sciences, Beijing, China; ^34^ Department of General Surgery, The First Affiliated Hospital of Harbin Medical University, Harbin, China; ^35^ Department of Anesthesiology, Peking University Third Hospital, Beijing, China; ^36^ National Center for Medical Service Administration, National Health Commission of the People’s Republic of China, Beijing, China; ^37^ Department of Physiology, Oklahoma University Health Science Center, Oklahoma City, OK, United States

**Keywords:** anaphylaxis, clinical practice guideline, epinephrine, emergency management, severity grading system

## Abstract

**Background:** For anaphylaxis, a life-threatening allergic reaction, the incidence rate was presented to have increased from the beginning of the 21st century. Underdiagnosis and undertreatment of anaphylaxis are public health concerns.

**Objective:** This guideline aimed to provide high-quality and evidence-based recommendations for the emergency management of anaphylaxis.

**Method:** The panel of health professionals from fifteen medical areas selected twenty-five clinical questions and formulated the recommendations with the supervision of four methodologists. We collected evidence by conducting systematic literature retrieval and using the Grading of Recommendations, Assessment, Development, and Evaluation (GRADE) approach.

**Results:** This guideline made twenty-five recommendations that covered the diagnosis, preparation, emergency treatment, and post-emergency management of anaphylaxis. We recommended the use of a set of adapted diagnostic criteria from the American National Institute of Allergy and Infectious Diseases and the Food Allergy and Anaphylaxis Network (NIAID/FAAN), and developed a severity grading system that classified anaphylaxis into four grades. We recommended epinephrine as the first-line treatment, with specific doses and routes of administration for different severity of anaphylaxis or different conditions. Proper dosage is critical in the administration of epinephrine, and the monitor is important in the IV administration. Though there was only very low or low-quality evidence supported the use of glucocorticoids and H1 antagonists, we still weakly recommended them as second-line medications. We could not make a well-directed recommendation regarding premedication for preventing anaphylaxis since it is difficult to weigh the concerns and potential effects.

**Conclusion:** For the emergency management of anaphylaxis we conclude that:

• NIAID/FAAN diagnostic criteria and the four-tier grading system should be used for the diagnosis

• Prompt and proper administration of epinephrine is critical.

## Introduction

### Background

Anaphylaxis is a severe, life-threatening, systemic allergic reaction that occurs rapidly after exposure to a sensitizing agent [(Sampson et al., 2006; Simons et al., 2011)] (Table 1). Anaphylaxis typically occurs within minutes to hours after exposure to an allergen [(Sampson et al., 2006; Soar et al., 2008)]. Signs and symptoms commonly appear in the mucosal, respiratory, cardiovascular, neurologic and gastrointestinal system [(Sampson et al., 2006; Simons et al., 2011)]. The most common triggers of anaphylaxis are food, insect venom, and medication [(Simons et al., 2011; Jiang et al., 2016)]. In the United States and Europe, incidence rates of anaphylaxis were reported to be 49.8 per 100,000 person-year [(Decker et al., 2008)], 1.5–7.9 per 100,000 person-year [(Panesar et al., 2013)] respectively; lifetime prevalence was estimated to range from 0.05–5.1% [(Lieberman et al., 2006; Panesar et al., 2013; Wood et al., 2014)]. The incidence rate was presented to have increased from the beginning of the 21st century [(Lee et al., 2017a; Yao et al., 2018)].

**TABLE 1:** Level of evidence and strength of recommendation described in GRADE [(Balshem et al., 2011; Schünemann et al., 2013)].

Grade	Definition	Type of study
High	We are very confident that the true effect lies close to that of the estimate of the effect	RCT without rating down
		Observational study rated up by two levels^a^
Medium	We are moderately confident in the effect estimate: The true effect is likely to be close to the estimate of the effect, but there is a possibility that it is substantially different	RCT rated down by one level^b^
		An observational study of increased quality^a^
Low quality	Our confidence in the effect estimate is limited: The true effect may be substantially different from the estimate of the effect	RCT rated down by two level^b^
		Observational study without rating up or down
Very low quality	We have very little confidence in the effect estimate: The true effect is likely to be substantially different from the estimate of effect	RCT with very low quality^b^
		Observational study rated down by one or two level^b^
		Case series
		Case report
		Consensus
Recommended intensity rating		
Strong	When the benefit or risk of an intervention clearly outweigh its counterpart, or clearly do not, guideline panels offer strong recommendations	
Weak	When the benefit-risk is uncertain—either because of low-quality evidence, or because evidence suggests that the balance is close—guideline panels offer week recommendations	

RCT: randomized control trial

a Factors that can increase the quality of the evidence include: large magnitude of an effect, dose-response gradient, effect of plausible residual confounding. The quality of evidence should only rarely be rated up if serious limitations are present.

b Factors that can reduce the quality of the evidence include: risk of bias, inconsistency of results, indirectness of evidence, imprecision, publication bias.

Underdiagnosis and undertreatment are still international public health concerns [(Simon and Mulla, 2008; Sclar and Lieberman, 2014; Jiang et al., 2016; Wang et al., 2017; Cohen et al., 2018; Prince et al., 2018; Choi et al., 2019)], and it is important to address the emergency management of anaphylaxis. A high-quality and comprehensive evidence-based guideline is needed to improve the capability of medical institutions at all levels in the emergency management of anaphylaxis. The National Center for Medical Service Administration (NCMSA) of the National Health Commission of the People’s Republic of China, which is associated with eight medical organizations, began the preparation of this practice guideline. This guideline is developed according to the criteria of the World Health Organization (WHO) handbook for guideline development [(World Health Organization, 2014)] and uses the Grading of Recommendations Assessment, Development, and Evaluation (GRADE) system [(Guyatt et al., 2011; Schünemann et al., 2013)]. Based on prior publications and the experience of experts from fifteen areas, this guideline selects and answers key questions that are key to multidisciplinary health care providers. Adherence to this guideline should improve the care of anaphylaxis patients at Chinese medical institutions. This guideline may also be beneficial to institutions worldwide.

### Scope and Purpose

This aim of this guideline is to provide graded recommendations to health care providers at all types of medical institutions. This guideline is intended to help providers effectively manage anaphylaxis in patients of all ages in a scientific manner by making recommendations for diagnosis, preparation, treatment, and management after emergency treatment. It is anticipated that this guideline would help to save more lives threatened by anaphylaxis around the world. Patients who have experienced anaphylaxis and their caregivers may also be informed by this guideline.

## Methods

**FIGURE 1:**
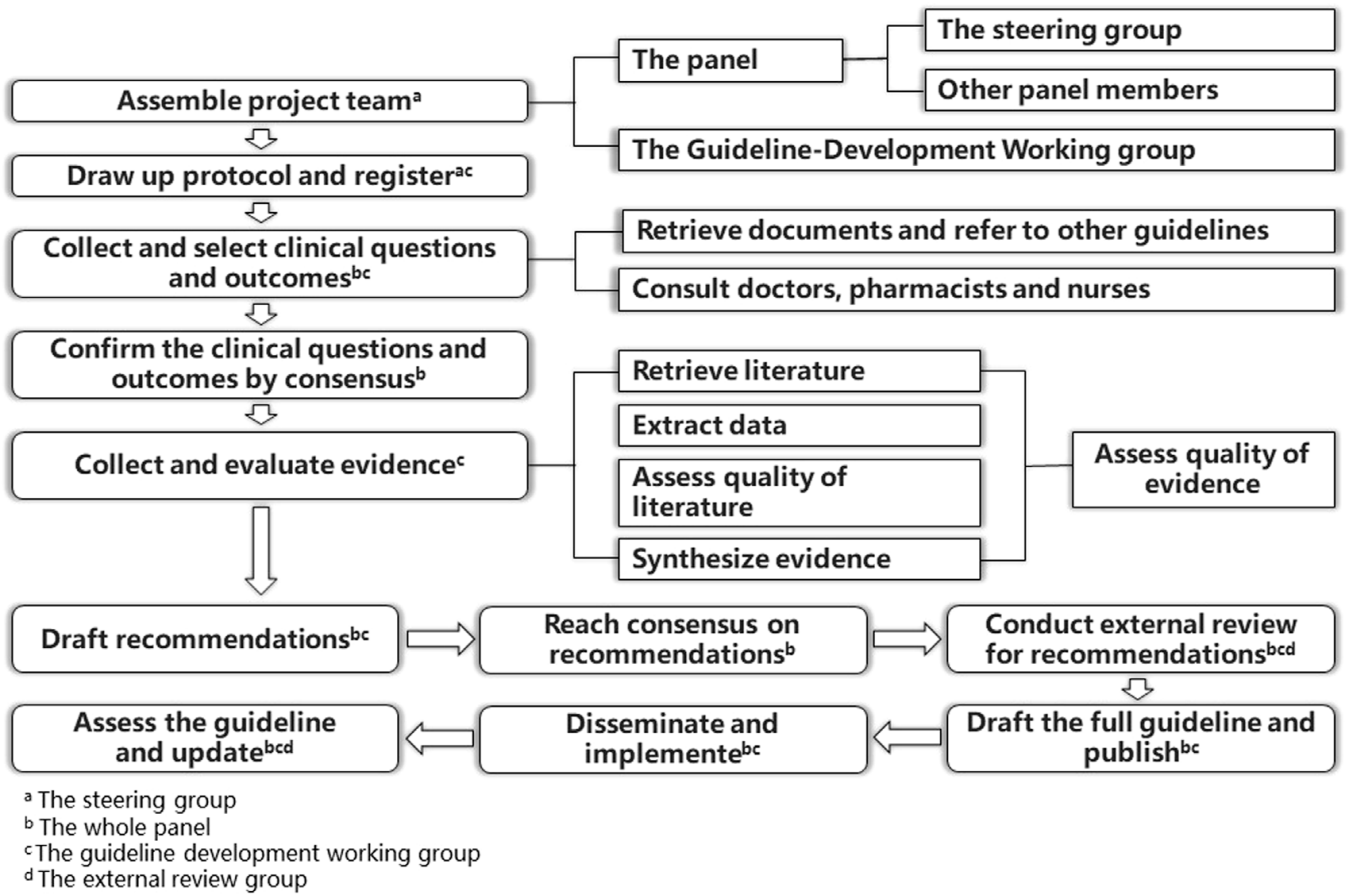
Guideline development process.

This guideline was developed according to the guideline development process set forth by the American Institute of Medicine [(Graham et al., 2011)] and World Health Organization handbook for guideline development [(World Health Organization, 2014)]. It followed the standard published in the Appraisal of Guidelines for Research and Evaluation (AGREE II) [(Brouwers et al., 2010)] and Reporting Items for Practice Guidelines in Healthcare [(Chen et al., 2017)] to ensure quality. The guideline development process is summarized in Figure 1.

### Group Composition and Meetings

The NCMSA launched the guideline project in July 2017 in cooperation with the Drug-Induced Diseases Professional Committee of Chinese Pharmacological Society, Chinese College of Allergy and Asthma, Chinese Society of Allergy, Chinese Society for Emergency Medicine, Chinese Thoracic Society, Chinese Society of Anesthesiology, Hospital Pharmacy Committee of Chinese Pharmaceutical Association, and the Chinese Nursing Association.

The Pharmacy Department of Peking University Third Hospital Pharmacy and Chinese GRADE Center provided technical support. A panel of thirty-four experts from fifteen areas was formed. Details about the team members of this project and their responsibilities are listed in Supplementary Appendix S1.

Five main meetings were held during the development process: three by the steering committee and two by the guideline panel (Supplementary Appendix S2). Additionally, views were shared electronically and small-scale working meetings were conducted.

### Retrieval, Synthesis and Assessment of the Evidence

We searched PubMed, embase, the Cochrane Library, Web of Science, three Chinese databases, clinicaltrials. gov and four guidelines databases (The National Institute for Health and Care Excellence Guidance, 2019; National Guideline Clearinghouse [Internet], 2019; Scottish Intercollegiate Guidelines Network [Internet], 2019; CMA Infobase, 2019) for randomized controlled trials (RCTs), cohort and case-control studies, and clinical guidelines. Since there were 25 clinical questions with sharing population (patients with anaphylaxis), we used one overall search strategy, which specified population and research types, to retrieve evidence for 25 question (Supplementary Appendix S3). We used the Cochrane risk-of-bias tool for randomized trials to assess RCTs (Higgins et al., 2011), used the instruments developed by the Clinical Advances through Research and Information Translation research group at McMaster University to assess risk of bias in cohort and case-control studies (Busse and Guyatt, 2020) and used AGREE II to assess the quality of guidelines (Brouwers et al., 2010).

We summarized evidence in summary of findings (SoF) tables that presented relative and absolute effects of diagnostic criteria and treatment using Grading of Recommendations Assessment, Development and Evaluation (GRADE) approach to assess the overall quality of the original evidence (Supplementary Appendix S4) [(Schünemann et al., 2013; Balshem et al., 2011)]. The quality of original evidence was divided into four levels: high, moderate, low, and very low (Table 1). We also used findings from published systematic reviews (quality assessed by using AMSTAR 2)[(Shea et al., 2017)] to supplement the primary literature. In addition, we assessed the quality and summarized the recommendations of clinical guidelines in Supplementary Appendix S6.

### Consensus on Recommendations

The GRADE system defines recommendations as either strong or weak based on the overall risk-benefit balance that considers the quality of the evidence equally with other factors such as the prognosis, potential for harm and/or for benefit (Table 1) [(Guyatt et al., 2008; Schünemann et al., 2013)]. For example, low quality of evidence of an intervention can result a strong recommendation if there are extremely bad prognosis, limited harm of intervention, and substantial benefit if it exists [(Schünemann et al., 2013)]. The panel reached the consensus of recommendations and their strength by a three-round modified Delphi method [(Dalkey and Helmer, 1963; Dalkey, 1969)], guaranteeing exchanging opinions and precluding peer group pressures [(Williams and Webb, 1994)].

### Other Procedures

Information about guideline registration, selection of clinical questions and outcomes and external review are described in Supplementary Appendices S6–S8.

### Supervision

The process of guideline development was supervised by four methodologists, including two experts in the area of guideline development, one in epidemiology and on in health economics.

## Results

This guideline addresses 25 clinical questions pertaining to the diagnosis, preparation, treatment, and management after emergency treatment of anaphylaxis. In the survey evaluating these recommendations, more than 90% of the 30 surveyed medical practitioners thought these recommendations were accurate, clearly stated and feasible (Supplementary Appendix S9).

**FIGURE 2:**
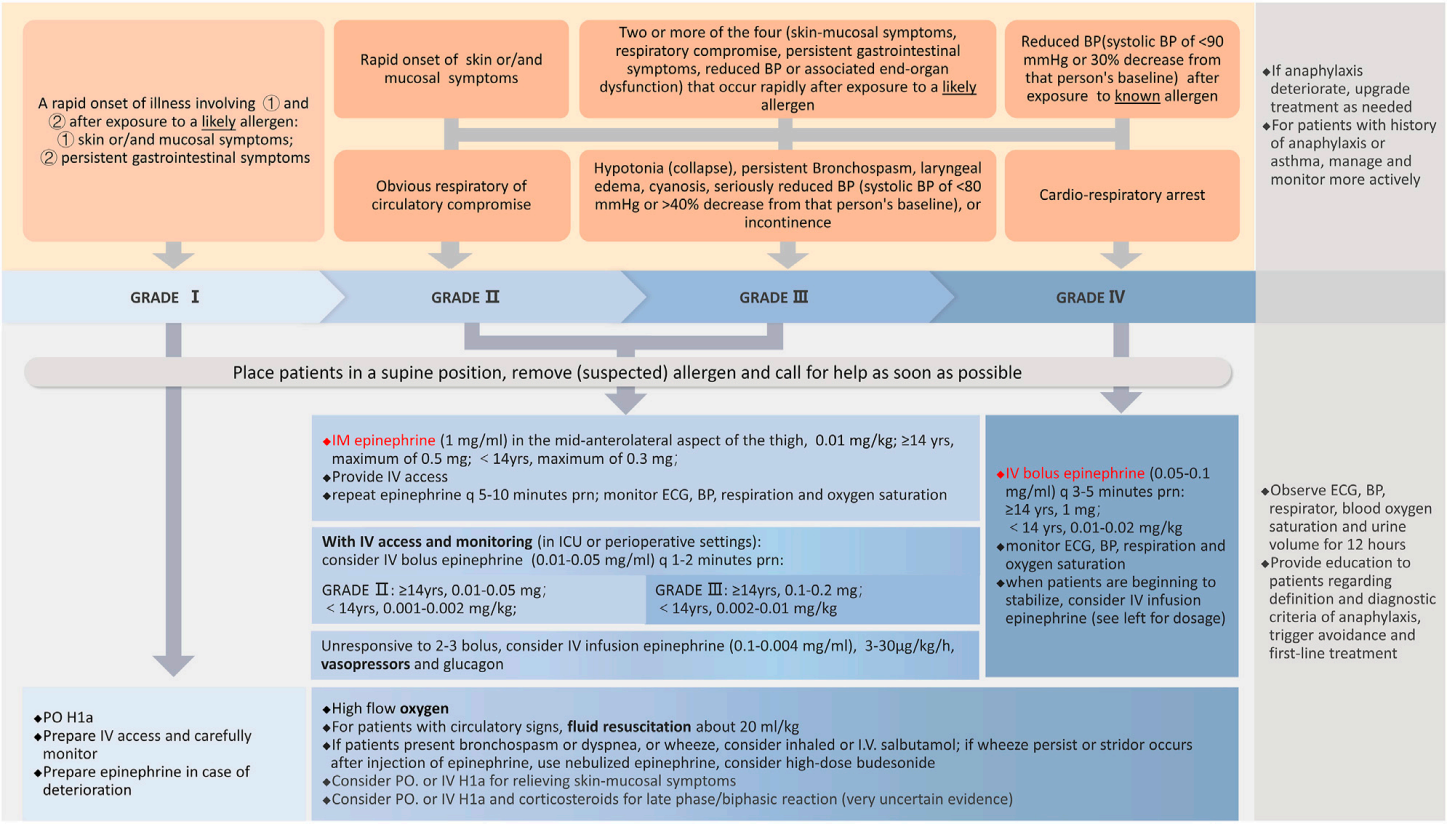
Anaphylaxis diagnosis and treatment flow chart.

The evidence was reported in three parts: (1) SoF tables in Supplementary Appendix S4 if original evidence is available, (2) recommendations of other guidelines [(Chamberlain, 1999; Lieberman et al., 2005; Kroigaard et al., 2007; Muraro et al., 2007; Soar et al., 2008; Harper et al., 2009; Tse and Rylance, 2009; Lieberman et al., 2010; Simons et al., 2011; Simons et al., 2012; Simons et al., 2013; Campbell et al., 2014; Muraro et al., 2014; Ring et al., 2014; Simons et al., 2014; Lieberman et al., 2015; Simons et al., 2015; Kolawole et al., 2017)] in Supplementary Appendix S6; (3) summary of original studies, systematic reviews and recommendations from other guidelines is displayed after every recommendation. Four systematic reviews contributed substantial information to the sections on the diagnosis of anaphylaxis, and the use of epinephrine, corticosteroids and pre-medications. Reommendations for diagnosis and emergency treatment are summarized in Table 2 and briefly organized as a flowchart (Figure 2).

**TABLE 2:** Summary of recommendations for emergency treatment.

No. of question	Recommendations
3	If a person is suspected to have anaphylaxis, inform the person, people nearby, or caregivers that an emergency call should be made immediately, or the patient should be transported directly to an emergency department for care by medical workers. While waiting for emergency medical technicians, the suspected allergen should be removed if possible. People should be placed on the back, or should be sitting up if there is respiratory distress. If vomiting occurs, ensure that the head is turned slightly downward and any substance in the airway should be cleared away to prevent aspiration. If an epinephrine pre-filled injector/auto-injector is available, they should follow the instructions written on the packaging or insert. (Strong recommendation)
4	Cardiovascular and respiratory function should be monitored closely (e.g., blood pressure, heart rate and rhythm, respiration rate, and oxygen saturation). (Strong recommendation)
5	Endotracheal intubation or supraglottic airway device insertion should be performed in the case of respiratory failure or severely labored breathing due to airway edema or bronchospasm. Tracheotomy or (needle) ricothyroidotomy may be considered in the case of an emergent “cannot intubate, cannot oxygenate” scenario or other emergencies. (Strong recommendation)
6.1	Epinephrine is the first-line medicine in GRADE II to IV anaphylaxis. (Strong recommendation)
6.2	Epinephrine should be administered as soon as possible in GRADE II anaphylaxis or higher. (Strong recommendation)
6.3	IM injection of epinephrine is the preferred route of administration in GRADE II and III anaphylaxis. (Strong recommendation)
6.4	The recommended dose of IM epinephrine is 0.01 mg/kg, up to a maximum of 0.5 mg for patients aged ≥14 years, and up to a maximum of 0.3 mg in patients <14 years old. Epinephrine concentration should be 1 mg/ml (1:1000), just the same as commercial preparations. Dosing may be repeated every in 5–15 min if there is no response. (Strong recommendation)
6.5	Intramuscular epinephrine should be injected in the mid-anterolateral thigh. (Strong recommendation)
6.6	IV bolus epinephrine should be administered in GRADE IV patients who face (imminent) cardio-respiratory arrest. GRADE II and GRADE III patients may be considered for IV bolus epinephrine if they already have venous access and are being monitored (i.e., ICU or perioperative patients). (Strong recommendation)
6.7	The dosing instructions for IV bolus of epinephrine is as follows
	GRADE IV: 1 mg for patients ≥14 years old; 0.01–0.02 mg/kg for patients <14 years old
	GRADE III: 0.1–0.2 mg for patients ≥14 years old; 0.002–0.01 mg/kg (2–10 μg/kg) for patients <14 years old
	GRADE II: 0.01–0.05 mg for patients ≥14 years old; 0.001–0.002 mg/kg (1–2 μg/kg) for patients <14 years old
	Commercial epinephrine for injection (1 mg/ml, i.e. 1:1000) must be diluted to a volume of 10–20 ml (0.05–0.1 mg/ml, i.e. 1: 20,000 to 1:10,000)
	If there is no response in 3–5 min (for GRADE IV) or 1–2 min (for GRADE II to III), then another dose of IV bolus epinephrine should be administered. (Weak recommendation)
6.8	In GRADE II or III anaphylaxis, epinephrine may be administered by IV infusion (ideally through infusion pump) when patients are unresponsive to 2–3 doses of IM/IV bolus epinephrine. These patients should already be monitored and have venous access established
	In patients experiencing GRADE IV anaphylaxis, IV infusion of epinephrine may be started when patients begin to stabilize even if cardio-pulmonary symptoms have not been completely relieved. (Weak recommendation)
6.9	The dose of epinephrine IV infusion should be 3–30 μg/kg/h. Epinephrine should be prepared by diluting the commercial solution of 1 mg/ml (1:1000) solution to 0.004–0.1 mg/ml (1:250,000–1:10,000), in a ratio of 1:250 to 1:10. (Weak recommendation)
6.10	SC injection of epinephrine for the emergency management of anaphylaxis is not recommended. (Strong recommendation)
6.11	There is no absolute contraindication to the use of epinephrine in emergency treatment of life-threatening anaphylaxis. However, it should be used with caution in patients with a history of cardiovascular disease and elderly patients. (Weak recommendation)
6.12	In order to reduce the risk of epinephrine-related ADRs, avoid IV epinephrine unless in recommended situations. If IV epinephrine is indicated, the proper concentration of epinephrine should be carefully prepared and checked. During IV administration, there should be continuous monitoring of ECG, BP, respiration, oxygen saturation. (Strong recommendation)
7	H1a is a second-line medicine that is used to relieve skin and mucosal symptoms in anaphylaxis. There is very uncertain evidence that H1a might reduce the risk of biphasic anaphylaxis or that its early administration may mitigate anaphylaxis severity. It may be administered orally or intravenously to patients who are Grade II or higher, and only after epinephrine has been given. In Grade I patients, the agents may be given orally (Weak recommendation)
8	Short-acting inhaled β2 agonist is a second-line medicine that can be used to treat lower respiratory tract symptom, such as bronchospasm, dyspnea, or wheezing. Salbutamol can be inhaled (commonly preferred) or administered intravenously. (Strong recommendation)
9	Glucocorticoids can be used as a second-line medication. Oral, IM or IV glucocorticoids may reduce the risk of biphasic or protracted anaphylaxis (very uncertain evidence). If bronchospasm persists or stridor occurs after epinephrine injection, high-dose nebulized budesonide can be considered. (Weak recommendation)
10	Fluid resuscitation is recommended for patients with circulatory signs. Initially, 20 ml/kg of fluids may be given, and then the amount may be adjusted according to response. (Strong recommendation)
11	After timely treatment, anaphylaxis patients should be monitored for at least 12 h in the hospital, with heartrate, BP, respiration, oxygen saturation, and urine volume. (Weak recommendation)
12	All cases of drug-induced anaphylaxis should be reported to an ADR surveillance system. The report should contain information about suspected triggers, symptoms and their timing relative to the drug exposure, management steps, and clinical outcomes. (Strong recommendation)
13	The key preventive measure is to avoid allergens. Prophylactic medications cannot be routinely used in the general population. Though some medications show the potential of reducing risk of anaphylaxis or related symptoms of drug-induced reactions, concerns remain about using them to prevent anaphylaxis (Table 6). (Weak recommendation)
14	At discharge, health care providers should teach patients and/or caregivers about anaphylaxis including: diagnostic criteria, avoidance of potential triggers, and first-line treatments. (Strong recommendation)

ADR, adverse drug reactions; BP, blood pressure; ECG, electrocardiogram; H1a, H1 antagonist; IM, intramuscular or intramuscularly; IV, intravenous or intravenously.

### Part 1. Diagnosis of Anaphylaxis

**Question 1. What are the clinical diagnostic criteria for anaphylaxis?**

Recommendation. Anaphylaxis should be diagnosis according to the criteria in Table 3. Clinicians should be aware that atypical anaphylaxis symptoms exist (Strong recommendation).

**TABLE 3:** Clinical criteria for the diagnosis of anaphylaxis^a^.

Anaphylaxis is highly likely when any one of the following three criteria are fulfilled
1. Acute onset of an illness (minutes to several hours) with involvement of the skin and/or mucosal tissue (e.g., generalized hives, pruritus or flushing, swelling of the lip, tongue, or uvula) and at least one of the following
a. Respiratory compromise (e.g., hoarseness, cough, chest tightness, dyspnea, wheeze-bronchospasm, stridor, cyanosis, reduced PEF, hypoxemia)
b. Reduced BP or associated symptoms of end-organ dysfunction [e.g., hypotonia (collapse), syncope, incontinence]
2. Two or more of the following that occur rapidly (minutes to several hours) after exposure to a likely allergen
a. Involvement of the skin-mucosal tissue (e.g., generalized hives, pruritus or flushing, swelling of the lip, tongue, or uvula)
b. Respiratory compromise (e.g., hoarseness, cough, chest tightness, dyspnea, wheeze-bronchospasm, stridor, cyanosis, reduced PEF, hypoxemia)
c. Reduced BP or associated symptoms (e.g., hypotonia [collapse], syncope, incontinence)
d. Persistent gastrointestinal symptoms (e.g., abdominal cramps, vomiting)
4. Reduced BP after exposure to a known allergen (within minutes to several hours)
a. Infants and children: low systolic BP (age specific) or >30% decrease in systolic BP^b^
b. Adults: systolic BP of <90 mmHg or >30% decrease from a baseline measurement

BP, blood pressure; PEF, peak expiratory flow.

a Adapted from diagnostic criteria proposed by American National Institute of Allergy and Infectious Diseases and the Food Allergy and Anaphylaxis Network (NIAID/FAAN)[ (Sampson et al., 2006)].

b Low systolic blood pressure in children is defined by age: 1 month to 1 year: < 70 mmHg; age 1–10 years: < [70 mmHg + (2 × age)]; age 11–17 years: < 90 mmHg.

Summary of the evidence. Pooled results of two high-quality studies (Loprinzi Brauer et al., 2016; Campbell et al., 2012) using the NIAID/FAAN diagnostic criteria showed that the sensitivity was 96% (95% CI, 91–99%; high-quality evidence) and specificity was 77% (95% CI, 72–82%; moderate-quality evidence) (Supplementary Table S1 in Supplementary Appendix S4). Only studies with high risk of bias (Enrique et al., 1999; Brown et al., 2004a; Laroche et al., 2014; Wongkaewpothong et al., 2014; Egner et al., 2016; Baretto et al., 2017; Francis et al., 2018; Vitte et al., 2019) were used to confirm the diagnostic accuracy of serum total mast cell tryptase (MCT) and histamine. Overall, the studies showed MCT and histamine may have a lower Youden’s index than the NIAID/FAAN criteria; having a lower sensitivity and higher specificity. Four out of eight guidelines have adopted the NIAID/FAAN diagnostic criteria and the remaining four guidelines recommended other criteria (see Supplementary Appendix S6).

Rationale. Underdiagnosis and undertreatment of anaphylaxis are potentially life-threatening and is an international concern (Bjornsson and Graffeo, 2010; Sclar and Lieberman, 2014; Cohen et al., 2018). The NIAID/FAAN criteria was highly sensitive for the detection of anaphylaxis and deserves to be a first-line assessment tool in all institutional settings (i.e., emergency departments (ED), operating rooms, intensive care units (ICU), and hospital departments of allergy, pediatrics, otolaryngology, dermatology, anesthesiology).

Comments. The skin-mucosal system is the most commonly involved body system, followed by the respiratory system/circulatory and neurological system, and then gastrointestinal system. However, not all cases involve skin or mucosal system symptoms (Brown, 2004; Simons et al., 2011; Worm et al., 2012; Muraro et al., 2014; Yoon et al., 2017; Aurich et al., 2019). In anaphylaxis events involving anaesthesia, respiratory and cardiovascular compromise are predominant while skin-mucosal problem are few (Harper et al., 2018). The time interval from exposure to an allergen to the onset of anaphylaxis symptoms was within 30 min in >50% of patients (Grabenhenrich et al., 2016; Yoon et al., 2017) and was within 1 h in most cases (Grabenhenrich et al., 2016; Harper et al., 2018). Children typically present with more cutaneous and respiratory symptoms than adults, and adults present with more of these symptoms than the elderly. Elderly patients appear to have more cardiovascular symptoms than adults, and these symptoms are more common in adults than in children (Yoon et al., 2017; Aurich et al., 2019). The NIAID/FAAN criteria were also recommended by the guideline from European Academy of Allergy and Clinical Immunology (Muraro et al., 2014; Muraro et al., 2022), the guideline from American Academy of Allergy, Asthma and Immunology and American College of Allergy, Asthma, and Immunology (AAAAI/ACAAI) (Campbell et al., 2014; Shaker et al., 2020), and the 2011 version of guideline from World Allergy Organization (WAO) (Simons et al., 2011). WAO amended the NIAID/FAAN criteria in 2020, but the accuracy of the revised criteria has not been validated yet (Cardona et al., 2020).

When the diagnosis of anaphylaxis is uncertain, laboratory tests after an episode can be used to provide supplemental evidence because the results may be more specific but less sensitive than the NIAID/FAAN criteria. MCT concentrations obtained within 3 h that are either elevated 1.5-times greater than baseline values (might be more accurate) or >11.4 μg/L, both suggest a high likelihood of anaphylaxis. Elevated peak histamine levels >1.0 μg/L obtained within 2 h after the onset of symptoms may also be indicative of anaphylaxis.

**Question 2. How is anaphylaxis classified or graded?**

Recommendation. Anaphylaxis should be graded as Table 4 (Strong recommendation).

**TABLE 4:** Severity grading system for anaphylaxis.

GRADE I	Both a and b below are met, and no signs or symptoms of cardiovascular or respiratory system involvement
	a. Involvement of the skin-mucosal tissue (e.g., generalized hives, pruritus flushing, swelling of lips, tongue, uvula)
	b. Persistent gastrointestinal symptoms (e.g., nausea, abdominal cramps, vomiting)
GRADE II	Either a or b below are met
	a. Respiratory compromise (e.g., hoarseness, cough, chest tightness, dyspnea, wheeze-bronchospasm, stridor, reduced PEF, SpO2 ≤ 92%)
	b. Reduced BP (systolic BP < 90 mmHg or >30% decrease from a baseline measurement) or associated symptoms of end-organ dysfunction (e.g., pallor, dizziness, diaphoresis, transient loss of consciousness, hypotonia [collapse], tachycardia)
GRADE III	Any of the following signs or symptoms are evident: cyanosis, systolic BP of <80 mmHg or >40% decrease from baseline measurement), loss of consciousness, hypersomnia, tachycardia, severe bronchospasm and/or laryngeal edema, incontinence, or other serious cardio- respiratory signs
GRADE IV	Cardio-respiratory arrest

BP: blood pressure; PEF: peak expiratory flow.

Grading is based on the most serious symptom observed; follow-up treatment is described in Part 3.

Summary of the evidence. Three clinical guidelines use the grading system as their standard for classifying the severity of anaphylaxis (Supplementary Appendix S6).

Rationale. The quality of evidence is very low; it is difficult to conduct high-quality research on the effectiveness of severity grading in anaphylaxis. However, assessment of severity is necessary for appropriate management. Guidelines generally rated skin-mucosal, gastrointestinal, respiratory/cardiovascular, and neurologic symptoms from mild to severe [(Kroigaard et al., 2007; Muraro et al., 2014; Ring et al., 2014; Kolawole et al., 2017)], as did published grading systems [(Brown, 2004; Eller et al., 2018)]. Respiratory and/or cardiovascular compromise are the leading cause of death from anaphylaxis [(Pumphrey, 2000; Pumphrey and Roberts, 2000; Brown, 2006; Greenberger et al., 2007)], so they are indicative of severe anaphylaxis.

Comments. The reader should combine this recommendation with the case-specific information to make clinical decisions (i.e., current physical status, comorbidities).

### Part 2. Preparation for Treatment of Anaphylaxis

**Question 3. What instructions should be given to patients or caregivers before the arrival of healthcare providers if anaphylaxis is suspected?**

Recommendation. If a person is suspected to have anaphylaxis, inform the person, people nearby, or caregivers that an emergency call should be made immediately, or the patient should be transported directly to an emergency department for care by medical workers. While waiting for emergency medical technicians, the suspected allergen should be removed if possible. People should be placed on the back, or should be sitting up if there is respiratory distress. If vomiting occurs, ensure that the head is turned slightly downward and any substance in the airway should be cleared away to prevent aspiration. If an epinephrine pre-filled injector/auto-injector is available, they should follow the instructions written on the packaging or insert (Strong recommendation).

Summary of the evidence. Two clinical guidelines provided recommendations on the steps that patients and caregivers should take during an anaphylaxis event (i.e., call for help, remove suspected allergens, elevate extremities, administer epinephrine, maintain and open airway, and other procedures) (Supplementary Appendix S6).

Rationale. Managing anaphylaxis is complicated and is best managed by a trained medical professional. Thus, the most important step for patients and caregivers is to call for medical help in a timely manner. The benefit of elevating the legs is controversial and is not recommended [(Markenson et al., 2010)].

Comment. If the patient needs to be moved, caregivers should use caution, and if possible, they should monitor vital signs (e.g., blood pressure, heart rate, temperature, respiration rate). In the event of a cardiac arrest, cardiopulmonary resuscitation should be started immediately.

### Part 3. Emergency Treatment of Anaphylaxis

**Question 4. How should the monitoring be performed for anaphylaxis patient during emergency management?**

Recommendation. Cardiovascular and respiratory function should be monitored closely (e.g., blood pressure, heart rate and rhythm, respiration rate, and oxygen saturation) (Strong recommendation).

Summary of the evidence. Six clinical guidelines provided recommendations for monitoring of cardio-pulmonary status (e.g., blood pressure, heart rate and rhythm, respiration rate, and oxygen saturation) (Supplementary Appendix S6).

Rationale. Anaphylaxis is life-threatening due to the potential collapse of the cardio-pulmonary system [(Pumphrey, 2000; Greenberger et al., 2007)]. Close monitoring of vital signs and related physiologic function will enable healthcare providers to make quick treatment decisions.

**Question 5. Under what circumstances artificial airways should be established and how to implement it?**

Recommendation. Endotracheal intubation or supraglottic airway device insertion should be performed in the case of respiratory failure or severely labored breathing due to airway edema or bronchospasm. Tracheotomy or (needle) ricothyroidotomy may be considered in the case of an emergent “cannot intubate, cannot oxygenate” scenario or other emergencies (Strong recommendation).

Summary of the evidence. No experimental studies were found that confirmed the effectiveness of artificial airway has been found. Five clinical guidelines have recommended the use of supraglottic airway devices, endotracheal intubation, or cricothyroidotomy in patients with severe laryngeal edema, severe stridor, or hypoventilation who are being ventilated by bag-valve masks (Supplementary Appendix S6).

Rationale. Respiratory arrest should take important responsibility in fatalities in anaphylaxis [(Pumphrey, 2000; Brown, 2006; Greenberger et al., 2007)], which underscores the importance of airway management.

Comment. Endotracheal intubation should be considered first because it is the least invasive. Intubation and supraglottic airway device insertion should be performed by experienced specialists because the procedure may be difficult and failure risks further airway closure and possibly death [(Frerk et al., 2015; Higgs et al., 2018)]. Surgical cricothyroidotomy is relatively contraindicated in young children because it poses a high risk of injury to surrounding structures [(Roberts and Hedges, 2013)]. Patients with a history of lung disease, especially asthma, are at higher risk for morbidity and mortality, thus early intubation should be considered during their assessment [(Bock et al., 2001; Pumphrey, 2004)].

**Question 6. How to use epinephrine correctly in the treatment of anaphylaxis?**

**Question 6.1 What is the role of epinephrine?**

Recommendation. Epinephrine is the first-line medicine in GRADE II to IV anaphylaxis (Strong recommendation).

Evidence summaries: Evidence on the use of epinephrine are showed in the SoF table (Supplementary Table S3 in Supplementary Appendix S4). A cohort study [(Fleming et al., 2015)] showed anaphylaxis patients who received epinephrine before arriving in an emergency department (ED) were less likely to be admitted to the hospital than those who waited to receive the drug in the ED [adjusted odds ratio (aOR) 0.25, 95% confidence interval (CI) 0.10 to 0.62, high-quality evidence). The same study noted that epinephrine probably reduced the median length of ED stay (3 vs. 4 h, *p* = 0.003, moderate-quality evidence]. Two cohort study indicated that (pre-ED) epinephrine was associated with higher risk of ICU admission in children (very low-quality evidence) [(Huang et al., 2012; Robinson et al., 2017)]. One case-control study reported that pre-ED epinephrine was associated with a reduced risk for ≥2 doses of epinephrine [(Hochstadter et al., 2016)], while two other case control studies reported that (prehospital) epinephrine did not influence or increased the odds of further treatment (very low-quality evidence)[(Russell et al., 2010; Gabrielli et al., 2019)]. One case control study suggested epinephrine was associated with a reduction in the risk of subsequent in-ED hypotension among patients with hemodynamically stable anaphylaxis (aOR 0.25, 95% CI 0.09–0.71, low-quality evidence) [(Ko et al., 2016)]. Our meta-analysis of 11 case-control studies [(Smit et al., 2005; Ellis and Day, 2007; Mehr et al., 2009; Lertnawapan and Maek-a-nantawat, 2011; Lee et al., 2014; Rohacek et al., 2014; Alqurashi et al., 2015; Manuyakorn et al., 2015; Sricharoen et al., 2015; Lee et al., 2017b; Kim et al., 2018)] reported that epinephrine had no or minimal effect on biphasic anaphylaxis (very low-quality evidence); so did another systematic review (Lee et al., 2015). Four systematic reviews found no RCTs that could confirm the effectiveness of epinephrine on managing anaphylaxis [(Sheikh et al., 2008; Sheikh et al., 2012; Dhami et al., 2014; Rubin et al., 2014)]. All eight clinical guidelines recommended epinephrine as the first choice of medication for the treatment of anaphylaxis (Supplementary Appendix S6).

Rationale. Animal experiments have demonstrated the vital role of epinephrine in the treatment of anaphylaxis [(Simons et al., 2011; Ma et al., 2017)]. Withholding epinephrine in placebo groups of clinical trials in anaphylaxis would be unethical. Nonetheless, observational studies, pharmacologic evidence, and extensive clinical experience by specialists all support the use of epinephrine. Epinephrine is more effective than H1a or glucocorticoids for the treatment of anaphylaxis (see question 8–10). Therefore, the importance of epinephrine in anaphylaxis should not be ignored due to the lack of RCTs.

Comment. Epinephrine should be readily available for anaphylaxis of all severity because in mild cases the condition may deteriorate rapidly.

**Question 6.2 When should epinephrine be initiated in anaphylaxis?**

Recommendation. Epinephrine should be administered as soon as possible in GRADE II anaphylaxis or higher. (Strong recommendation).

Summary of the evidence. The cohort study by Fleming et al. provided moderate to low-quality evidence to support the early administration of epinephrine (Supplementary Table S4 in Supplementary Appendix S4). Six case-control studies showed inconsistent results for the reduction in risk of biphasic anaphylaxis in early versus late administration of epinephrine [(Mehr et al., 2009; Scranton et al., 2009; Lertnawapan and Maek-a-nantawat, 2011; Alqurashi et al., 2015; Sricharoen et al., 2015; Kim et al., 2018)]. Seven clinical guidelines stated that epinephrine should be injected to anaphylaxis patient as soon as possible (Supplementary Appendix S6).

Rationale. Anaphylaxis can progress rapidly leading to potentially life-threatening respiratory and cardiac arrest within minutes after the onset [(Pumphrey, 2000; Commins, 2017)], which address the importance of early management. This is also supported by animal studies. Early use of epinephrine in mice injected with lethal doses of platelet activating factor reduced mortality [(Ma et al., 2017)]. In a dog model, delay of epinephrine until the onset of shock (i.e., a reduction in mean arterial pressure to 50% of baseline) failed to hasten hemodynamic recovery [(Bautista et al., 2002)].

**Question 6.3 For those who are eligible to receive epinephrine, which patients should receive intramuscular (IM) epinephrine?**

Recommendation. IM injection of epinephrine is the preferred route of administration in GRADE II and III anaphylaxis. (Strong recommendation).

Summary of the evidence. the SoF table in Supplementary Appendix S4 (Supplementary Tables S5–S8) summarizes evidence for the routes of administration for epinephrine. In an randomized control trial (RCT), the time to maximum plasma epinephrine concentration (Tmax) was shorter with IM versus subcutaneous (SC) epinephrine (8 *versus* 34 min, absolute difference -26 min, 95% CI -35 to -17 min, moderate-quality evidence), while the area under the plasma concentration versus time curve (AUC) was also greater (low-quality evidence) [(Simons et al., 1998)]. In two trials there were inconclusive results on peak plasma epinephrine concentrations (Cmax) for IM versus SC [(Simons et al., 1998; Simons et al., 2001)]. In an RCT, no serious adverse reaction was observed in patients who received either IM or SC injections [(Simons et al., 1998)]. A crossover RCT reported that mild transient adverse reactions were slightly higher with IM compared with SC epinephrine (low-quality evidence)[(Simons et al., 2001)]. A cohort study observed that there was no difference in the risk of overdose between IM and SC group [(Jiang et al., 2020)].

In a cohort study, IM epinephrine had a lower risk of cardiovascular complications compared with the intravenous (IV) route in [relative risk (RR) 0.02, 95% CI 0.00–0.11, low quality-evidence] [(Kawano et al., 2017a)]. Another cohort study also observed a lower risk of adverse cardiovascular event when compared to IV bolus (low-quality evidence) [(Campbell et al., 2015)]. Besides, two cohort studies indicated a lower risk of overdose in comparison with IV bolus (very low-quality evidence)[(Campbell et al., 2015; Jiang et al., 2020)]. However, there was no cardiovascular safety advantage for IM compared with IV infusion (very low-quality evidence) [(Campbell et al., 2015)].

Three guidelines recommended IM as the preferred route of administration for patients without cardio-respiratory arrest; another three guidelines recommended IM for patients without cardio-respiratory arrest except those who have established venous access in ICU or during perioperative period (Supplementary Appendix S6).

Rationale. While AUC and Cmax were not significantly different than SC epinephrine, the IM epinephrine with shorter Tmax exerts its pharmacologic action faster. With regard to safety, IM epinephrine appears to be safer than the IV route (particularly IV bolus), and nearly as safe as SC epinephrine. Therefore, in patients not experiencing cardio-respiratory arrest, the IM route of injection is recommended for epinephrine.

**Question 6.4 What is the dose of IM epinephrine?**

Recommendation. The recommended dose of IM epinephrine is 0.01 mg/kg, up to a maximum of 0.5 mg for patients aged ≥14 years, and up to a maximum of 0.3 mg in patients <14 years old. Epinephrine concentration should be 1 mg/ml (1:1000), just the same as commercial preparations. Dosing may be repeated every in 5–15 min if there is no response (Strong recommendation).

Summary of the evidence. One systematic review found no RCTs evaluating the effect of different doses of epinephrine [(Dhami et al., 2014)]. Four clinical guidelines recommended dosage as 0.01 mg/kg, concentration as 1 mg/ml (1:1000) with the maximum dose for adults as about 0.5 mg and the maximum dose for children as about 0.3 mg; four guidelines recommended specific doses for adults and children; six guidelines recommended intervals of 5–15 min for repeated dose (Supplementary Appendix S6).

Rationale. This dosing standard has been in place for many years [(Simons et al., 2011; Muraro et al., 2014)] and is supported by safety data [(Campbell et al., 2015; Kawano et al., 2017a)]. The Tmax of IM epinephrine was reported to be 8 ± 2 min [(Simons et al., 1998)], which supports the re-dosing interval of 5–15 min that is recommended in six guidelines.

Comments. Close observation is required for the response to epinephrine. Establish venous access and be prepared to give a second epinephrine dose.

**Question 6.5 At which site should the IM epinephrine be administered?**

Recommendation. Intramuscular epinephrine should be injected in the mid-anterolateral thigh. (Strong recommendation).

Summary of the evidence. Evidence is summarized in the SoF table (Supplementary Table S9 in Supplementary Appendix S4). A randomized cross-over trial comparing IM injections into the thigh or upper arm showed that the Cmax of epinephrine was higher when given in the thigh (low-quality evidence) [(Simons et al., 2001)]. This trial also provided very uncertain evidence that IM injection into the thigh may increase the risk of mild transient adverse effects (very low-quality evidence). No other comparative studies were found [(Dhami et al., 2014)]. All five clinical guidelines recommended the (medial) latera thigh as the injection site (Supplementary Appendix S6).

Rationale. IM injections to the mid-anterolateral thigh delivers more drug without serious safety concerns. Thus, it is preferred even if some healthcare providers may be accustomed to upper arm injections [(Chooniedass et al., 2017)].

**Question 6.6 For those who should be prescribed epinephrine, which patients should receive epinephrine by IV bolus?**

Recommendation. IV bolus epinephrine should be administered in GRADE IV patients who face (imminent) cardio-respiratory arrest. GRADE II and GRADE III patients may be considered for IV bolus epinephrine if they already have venous access and are being monitored (i.e., ICU or perioperative patients). (Strong recommendation).

Summary of the evidence. As described in the recommendation of Question 6.3: the incidence rate of adverse cardiovascular events and overdose was higher with IV bolus versus IM epinephrine (low-quality evidence) (Supplementary Table S6 in Supplementary Appendix S4) [(Campbell et al., 2015; Kawano et al., 2017a; Jiang et al., 2020)]. Six clinical guidelines recommended epinephrine IV bolus for patients who are experiencing (imminent) cardio-pulmonary arrest, unresponsive after 2-3 injections of IM epinephrine, or undergoing monitoring in the perioperative setting (Supplementary Appendix S6).

Rationale. Serious adverse events associated with IV epinephrine include arrhythmia, hypertensive crisis, and pulmonary edema [(Campbell et al., 2015)]. However, Epinephrine given as IV bolus improves spontaneous circulation more rapidly than other routes and the time saved may be critical in patients who are at risk for cardio-pulmonary arrest [(Link et al., 2015; Gough and Nolan, 2018)]. Hence, the benefits outweigh the risks in patients facing imminent circulatory collapse. In addition, the risk is mitigated through the effective use of monitoring equipment that ensure the safety of patient. Hence, for patients with careful hemodynamic monitoring, the proper use of IV bolus epinephrine is thought to exert its effect safely and rapidly [(Kroigaard et al., 2007; Harper et al., 2009; Kolawole et al., 2017)].

Comments. Continuous clinical monitoring is critical in patients receiving IV epinephrine because of the risk of adverse events.

**Question 6.7 What is the dose of IV bolus epinephrine?**

Recommendation. The dosing instructions for IV bolus of epinephrine is as follows:

GRADE IV: 1 mg for patients ≥14 years old; 0.01–0.02 mg/kg for patients <14 years old; GRADE III: 0.1–0.2 mg for patients ≥14 years old; 0.002–0.01 mg/kg (2–10 μg/kg) for patients <14 years old; GRADE II: 0.01–0.05 mg for patients ≥14 years old; 0.001–0.002 mg/kg (1–2 μg/kg) for patients <14 years old; Commercial epinephrine for injection (1 mg/ml, i.e. 1:1000) must be diluted to a volume of 10–20 ml (0.05–0.1 mg/ml, i.e. 1: 20,000 to 1:10,000).

If there is no response in 3–5 min (for GRADE IV) or 1–2 min (for GRADE II to III), then another dose of IV bolus epinephrine should be administered (Weak recommendation).

Summary of the evidence. No original study has been retrieved. Recommendations from seven guidelines are showed in Supplementary Appendix S6. Six clinical guidelines recommended a dosage in GRADE II and III anaphylaxis of 0.02–0.2 mg for adults, and 0.001–0.01 mg/kg (1–10 μg/kg) for children <12 years old; in GRADE IV anaphylaxis, 1 mg for adults, and 0.005–0.02 mg/kg (5–20 μg/kg) for children <12 years old; the recommended concentration and dosing interval was 1:10,000 (0.1 mg/ml) to 1:100,000 (0.01 mg/ml) and 1–5 min. One guideline recommended titrating according to response in the presence of continuous hemodynamic monitoring. All guidelines recommended a slow IV bolus administration.

Comment. There is a high risk of adverse cardiovascular event and overdose with IV bolus epinephrine [(Simons et al., 2011; Campbell et al., 2015)]. The stock concentration of epinephrine must be diluted and all preparations should be carefully checked by at least one other professional.

**Question 6.8 For those who are eligible to receive epinephrine, which patients should receive epinephrine by IV infusion?**

Recommendation. In GRADE II or III anaphylaxis, epinephrine may be administered by IV infusion (ideally through infusion pump) when patients are unresponsive to 2-3 doses of IM/IV bolus epinephrine. These patients should already be monitored and have venous access established.

In patients experiencing GRADE IV anaphylaxis, IV infusion of epinephrine may be started when patients begin to stabilize even if cardio-pulmonary symptoms have not been completely relieved (Weak recommendation).

Summary of the evidence. Findings are summarized in the SoF table (Supplementary Tables S6, S8 in Supplementary Appendix S4). As described in the recommendation of Question 6.7, IV administration might increase the risk of cardiovascular complications (low-quality evidence) (Kawano et al., 2017a) but a related study did not find IV infusion would do so (very low-quality evidence) (Campbell et al., 2015). Eight clinical guidelines suggested that patients who require multiple doses of epinephrine may benefit from epinephrine IV infusion if they have venous access and adequate monitoring (Supplementary Appendix S6).

Rationale. Infusion by IV pump exerts a rapid pharmacologic effect and have advantages in dosing titration. Use of epinephrine IV infusion is thought to be effective and well tolerated when the rate of infusion is carefully adjusted according to patient response [(Kroigaard et al., 2007; Harper et al., 2009; Simons et al., 2011; Kemp and Kemp, 2014; Lieberman et al., 2015; Kolawole et al., 2017)]. IV infusion can be dangerous for patients in the absence of monitoring [(Soar et al., 2008; Simons et al., 2011; Kolawole et al., 2017)], and considering that establishing IV access and monitoring procedures is time consuming, so prompt IM epinephrine may be more suitable.

**Question 6.9 What is the dose of epinephrine IV infusion?**

Recommendation. The dose of epinephrine IV infusion should be 3–30 μg/kg/h. Epinephrine should be prepared by diluting the commercial solution of 1 mg/ml (1:1000) solution to 0.004–0.1 mg/ml (1:250,000–1:10,000), in a ratio of 1:250 to 1:10. (Weak recommendation).

Summary of the evidence. Dosage studies were not found. Seven clinical guidelines suggested dosages of 2–120 μg/kg/h using a concentration of 0.001 mg/ml (1:1,000,000) to 0.1 mg/ml (1:10,000) ml (Supplementary Appendix S6).

Comment. A dosing error in the administration of epinephrine can be life-threatening (Kanwar et al., 2010). Dose and concentration in epinephrine IV infusion can be complicated, so health professionals should pay close attention to the final epinephrine dose and concentration. A concentration of 0.01–0.05 mg/ml (1:100–1:20) is suitable for most cases because it may be easier to modify the infusion rate in this range.

**Question 6.10 For those who are eligible to receive epinephrine, which patients should receive epinephrine by subcutaneous injection?**

Recommendation. SC injection of epinephrine for the emergency management of anaphylaxis is not recommended. (Strong recommendation).

Summary of the evidence. Findings are summarized in the SoF table (Supplementary Table S5 in Supplementary Appendix S4). As described in the recommendations of Questions 6.3: SC route likely delays the effect of epinephrine without obvious difference in safety compared with IM administration (low-quality evidence) [(Simons et al., 1998; Simons et al., 2001; Jiang et al., 2020)]. Five guidelines explicitly did not recommend the SC route in administrating epinephrine (Supplementary Appendix S6).

Rationale. SC administration of epinephrine delays the onset of effect because subcutaneous tissue is not well perfused (Jin et al., 2015).

**Question 6.11 What are the contraindications for epinephrine in anaphylaxis patients?**

Recommendation. There is no absolute contraindication to the use of epinephrine in emergency treatment of life-threatening anaphylaxis. However, it should be used with caution in patients with a history of cardiovascular disease and elderly patients (Weak recommendation).

Summary of the evidence. No original study has been retrieved. A systematic review of poor quality included case reports that did not find evidence to contraindicate the use of SC epinephrine in older patients without a history of coronary artery disease (Safdar et al., 2001). No contraindications were noted in four clinical guidelines. These guidelines remarked that there is some risk of adverse events in the elderly and patients with cardiovascular disease, but in most cases, the benefits outweigh the risk (Supplementary Appendix S6).

Rationale. In general, caution should be used in patients with coronary heart disease, cardiomyopathy, uncontrolled hypertension, diabetes mellitus, hyperthyroidism or glaucoma (Par Pharmaceutical, 2019). The effect of epinephrine may be potentiated in patients concurrently taking long-term monoamine oxidase inhibitors (Product Information, 2004). Epinephrine use in pregnant women with blood pressure (BP) ≥ 130/80 mmHg may present a risk to the fetus (Product Information, 2004). Nonetheless, epinephrine remains the most effective option in these patients and anaphylaxis poses a greater risk of harm than the potential adverse drug event.

**Question 6.12 What methods can be used to mitigate and treat adverse drug reactions caused by epinephrine?**

Recommendation. In order to reduce the risk of epinephrine-related adverse drug reactions (ADRs), avoid IV epinephrine unless in recommended situations. If IV epinephrine is indicated, the proper concentration of epinephrine should be carefully prepared and checked. During IV administration, there should be continuous monitoring of electrocardiogram, BP, respiration, oxygen saturation (Strong recommendation).

Summary of the evidence. Among seven guidelines that had related recommendations, six stressed the importance of circulatory and respiratory monitoring, particularly in the IV administration of epinephrine; three guidelines remarked on the importance of the concentration of epinephrine and the rate of infusion or injection (Supplementary Appendix S6).

Rationale. Common adverse reactions to epinephrine are palpitations, pallor, sweating, nausea, vomiting, weakness, dizziness, headache, tremors, anxiety, and difficulty breathing (each occurring in 1–10% of patients). Serious adverse reactions include: ventricular arrhythmias, acute hypertension, cerebral hemorrhage, pulmonary edema, and injection site infections (Cardona et al., 2017; IBM Micromedex, 2019).

Inappropriate dose or concentration of epinephrine might lead to devastating consequences (Institute for Safe Medica, 2018). Therefore, dose, concentration and administrating speed should be paid attention to, and patients should be carefully monitored when receive IV epinephrine.

Comment. When regional epinephrine adverse reactions (e.g., white, partially weaker perception) occurs, 0.5–1.5 mg phentolamine diluted in 1 ml sodium chloride injection could be used by local invasion at epinephrine injection site (Cluck et al., 2013).

Comment for question 6: Some studies indicates that inhalation [(Mellem et al., 1991; Frechen et al., 2015)] or intranasal [(Srisawat et al., 2016)] epinephrine administration do well in pharmacokinetics. Still, because of the uncertainty of dose and bad taste of epinephrine, they should not be substituted for injection of epinephrine [(Simons et al., 2000; Simons et al., 2011)]. However, nebulized epinephrine administered through a tracheal tube, or a mask with compressor, might decrease edema and obstruction in the oropharynx and larynx [(Lieberman et al., 2010; Simons et al., 2011; Muraro et al., 2014; Lieberman et al., 2015)].

**Question 7. What is the role of H1 antagonist?**

Recommendation. H1 antagonist (H1a) is a second-line medicine that is used to relieve skin and mucosal symptoms in anaphylaxis. There is very uncertain evidence that H1a might reduce the risk of biphasic anaphylaxis or that its early administration may mitigate anaphylaxis severity. It may be administered orally or intravenously to patients who are Grade II or higher, and only after epinephrine has been given. In Grade I patients, the agents may be given orally (Weak recommendation).

Summary of the evidence. Findings are summarized in the SoF table (Supplementary Table S10 in Supplementary Appendix S4). The body evidence for this question is very uncertain.

A case-control study (Gabrielli et al., 2019) reported that prehospital antihistamine may narrowly reduce the risk of hospitalization while a cohort study (Russell et al., 2010) found the overall use of antihistamine may have an opposite result (very low-quality evidence). Two case-control studies (Hochstadter et al., 2016; Gabrielli et al., 2019) suggested that prehospital antihistamine may reduce the risk of further treatment, when one of them (Hochstadter et al., 2016) found that in-ED antihistamine may lead to a contrary result (very low-quality evidence).

A pooled-analysis of nine case-control studies (Smit et al., 2005; Ellis and Day, 2007; Mehr et al., 2009; Scranton et al., 2009; Lertnawapan and Maek-a-nantawat, 2011; Rohacek et al., 2014; Alqurashi et al., 2015; Sricharoen et al., 2015; Kim et al., 2018) showed that antihistamines may have a slight reduction in the risk of biphasic anaphylaxis [odds ratio (OR) 0.70, 95% CI 0.38 to 1.40, very low-quality evidence]. A case-control study (Ko et al., 2016) likewise also suggested that H1a may lower the risk of subsequent in-ED hypotension (OR 0.70, 95% CI 0.20 to 2.42, very low-quality evidence).

Three systematic reviews did not list any pertinent high-quality studies on this topic (Sheikh et al., 2007; Dhami et al., 2014; Rubin et al., 2014). Eight clinical guidelines recommend PO, IV, or IM administration of H1a as a second or third-line treatment, stating that it might be useful to alleviate cutaneous symptoms or prevent biphasic reactions (Supplementary Appendix S6).

Rationale. Some studies showed that administration of H1a was associated with higher risk of hospitalization (Russell et al., 2010) and further treatment (Hochstadter et al., 2016). One possible explanation is that in-ED H1a aggravates the anaphylaxis, but the more reasonable one is that severe patients are more likely to receive antihistamines (indication bias). Still, most evidence (Supplementary Table S10 in Supplementary Appendix S4) showed a beneficial point estimate, which suggested a favorable effect, though the 95% CI crossing the null point significantly reduced the certainty (Zeng et al., 2021). The AAAAI/ACAAI guideline (Shaker et al., 2020) (2020) also reported a favorable estimate (0.71) in their meta-analysis for the effect of H1a on biphasic anaphylaxis, with an insignificant 95% CI (0.47–1.06) that considerably reduce the precision of the evidence (Zeng et al., 2021). Although all positive results are of very low quality and AAAAI/ACAAI made recommendation against the use of H1a in preventing biphasic anaphylaxis, considering H1a general have a good safety profile, there might be serious indication bias, and the underlying mechanism for the benefits is theoretically possible, we think the overall effect of H1a might be beneficial.

Comment. H1a agents are not a substitute for epinephrine. Up to 1-in-4 patients experience biphasic reactions (0.4–23.3%), clinically defined as symptoms that recur after the initial resolution of anaphylaxis without further exposure to allergens [(Tole and Lieberman, 2007; Simons et al., 2011; Lee et al., 2015)]. The onset time of biphasic anaphylaxis ranges from minutes to several days [(Lee et al., 2015)].

The recommended dose of H1a are as follow: diphenhydramine, 20–50 mg for adults, 1 mg/kg for children (up to a maximum of 50 mg); chlorpheniramine, 10 mg for adults, 2.5–5 mg for children; clemastine, 2 mg for adults and 0.0125–0.025 mg/kg for children.

**Question 8. What is the role of inhaled β2-agonist?**

Recommendation. Short-acting inhaled β2 agonist is a second-line medicine that can be used to treat lower respiratory tract symptom, such as bronchospasm, dyspnea, or wheezing. Salbutamol can be inhaled (commonly preferred) or administered intravenously. (Strong recommendation).

Summary of the evidence. Two systematic reviews did not cite any relevant high-quality studies of β_2_ agonist in anaphylaxis (Dhami et al., 2014; Rubin et al., 2014). Eight clinical guidelines recommended inhaled β_2_ agonist, mainly salbutamol, to relieve bronchospasm; five of them stated that β_2_ agonists might need to be delivered via nebulization (Supplementary Appendix S6).

Rationale. β_2_ agonist exerts it is bronchodilatory effects on β_2_ adrenoceptors located on airway smooth muscle cells (Billington et al., 2017). Though no direct evidence supports the use of β_2_ agonists for the management of anaphylaxis, it is effect on anaphylaxis can be extrapolated from evidence of effectiveness in patients with asthma (Global initiative for ast, 2020; Global Initiative for Asthma, 2020).

Comment. The suggested dose for salbutamol is 2–12 puffs by metered dose inhaler with a spacer; or 2–5 mg in 3 ml of saline by nebulizer; or 0.1–0.4 mg administered intravenously.

**Question 9. What is the role of glucocorticoids?**

Recommendation. Glucocorticoids can be used as a second-line medication. Oral, IM or IV glucocorticoids may reduce the risk of biphasic or protracted anaphylaxis (very uncertain evidence). If bronchospasm persists or stridor occurs after epinephrine injection, high-dose nebulized budesonide can be considered (Weak recommendation).

Summary of the evidence. Findings are published in the systematic review and summarized in SoF table (Supplementary Table S11 in Supplementary Appendix S4). A case-control study (Gabrielli et al., 2019) reported that glucocorticoid use increased the risk of hospitalization in anaphylaxis patients (aOR 2.88, 95% CI 1.13–7.36, low-quality evidence) while a cohort study suggested glucocorticoids reduce the risk of ICU admission (RR 0.77, 95% CI 0.56–1.07 very low-quality evidence). Similarly, another cohort study (Michelson et al., 2015) reported glucocorticoids may reduce the risk of prolonged length of hospital stay of ≥2 days (aOR 0.61, 95% CI 0.41–0.93, low-quality evidence) whereas a third cohort study (Okubo et al., 2018) showed glucocorticoid use may prolong hospital stays by 0.39 days (95% CI 0.29–0.49 days, very low-quality evidence). One case-control study (Gabrielli et al., 2019) reported an inconclusive association between glucocorticoids and the need for further treatment, namely, a positive correlation with rate of ED use of IV fluids and a negative association with the risk of ≥2 epinephrine doses (very low-quality of evidence). Three cohort studies (Grunau et al., 2015; Michelson et al., 2015; Okubo et al., 2018) reported that glucocorticoids may have little to no effect on allergy-related hospital revisit (very low-quality evidence). The meta-analysis of 12 case-control studies (Smit et al., 2005; Ellis and Day, 2007; Mehr et al., 2009; Scranton et al., 2009; Lertnawapan and Maek-a-nantawat, 2011; Lee et al., 2014; Rohacek et al., 2014; Alqurashi et al., 2015; Manuyakorn et al., 2015; Sricharoen et al., 2015; Lee et al., 2017b; Kim et al., 2018) suggested glucocorticoids may slightly reduce the risk of biphasic anaphylaxis (OR 0.84, 95% CI 0.43 to 1.63, very low-quality evidence). Glucocorticoids may also reduce the risk of subsequent in-ED hypotension (OR 0.70, 95% CI 0.20 to 2.42, very low-quality evidence) (Ko et al., 2016), and the risk use of epinephrine administration beyond the day of anaphylaxis-onset (aOR 0.63, 95% CI 0.48 to 0.84, low quality evidence) (Michelson et al., 2015).

Four systematic reviews found no clinical trial on glucocorticoids in anaphylaxis (Choo et al., 2010; Choo et al., 2012; Dhami et al., 2014; Rubin et al., 2014; Liyanage et al., 2017). One meta-analysis of case series and observational studies reported that the administration of steroids may be associated with an increased risk of biphasic reaction, but the difference was not significant (Tole and Lieberman, 2007). Eight guidelines made recommendations on the use of glucocorticoids, most of them as second-line or auxiliary treatments (Supplementary Appendix S6). They reported that the agents, given parenterally or orally, may be beneficial in relieving respiratory symptoms and preventing biphasic/protracted anaphylaxis. One guideline also recommended nebulized glucocorticoids.

Rationale. The effect of suppressing the immune system of glucocorticoids can take several hours (Williams, 2018), so it may be difficult for it to be effective in acute phase treatment. The evidence is poor and controversial regarding the effect of glucocorticoids for hospitalization, length of hospital stay, and allergy-related re-hospitalization. However, patients with more severe anaphylaxis may be more likely to receive glucocorticoids (indication bias). Even with potentially serious indication bias, some studies still observed some beneficial effects of glucocorticoids (Michelson et al., 2015; Ko et al., 2016; Gabrielli et al., 2019). Besides, the updated guideline from AAAAI/ACAAI (2020) made a weak recommendation against the use of glucocorticoids in the prevention of anaphylaxis, but they did report a favorable point estimation as 0.87, even after pooling all adjusted and unadjusted observational studies using different definitions of the outcome, which might be seriously affected by the aforementioned indication bias (Shaker et al., 2020). The 95% CI reported in AAAAI/ACAAI guideline (0.74–1.02) did significantly impair the certainty (Shaker et al., 2020), but this might be an underestimation of the effect due to indication bias. Additionally, given the role of glucocorticoids in managing other types of allergic reactions (Pourmand et al., 2018; Global initiative for ast, 2020), glucocorticoids might have the potential to reduce the length of hospital stay and/or the risk of biphasic anaphylaxis.

The use of nebulized budesonide in relieving bronchospasm is extrapolated from evidence in other allergic reactions and it is very uncertain (Muraro et al., 2014; Global initiative for ast, 2020). However, considering limited harm and potentially substantial benefit (if it exists), this should be a consideration.

Comment. Glucocorticoids are not a substitute for epinephrine and their use should not preclude or precede epinephrine. Table 5 summarizes the dosage and administration of three common parenteral glucocorticoids: hydrocortisone, methylprednisolone, and dexamethasone. Oral prednisone or prednisolone may be administered (0.5–1 mg/kg, up to a maximum of 50 mg).

**TABLE 5:** Dosage and administration of corticosteroid injections.

Agent	Route of administration	Dose	Maximum dose for adults (mg)	Maximum dose for children (mg)	Notes
Hydrocortisone	IV or IM	2–4 mg/kg	200	100	Rapid onset, but may contain alcohol, which may be hazardous in the management of anaphylaxis
Methylprednisolone	IV or IM	1–2 mg/kg	100	50	Rapid onset, but may not available at the primary care level
Dexamethasone	IV or IM	0.1–0.4 mg/kg	20	10	Slow onset, inexpensive, widely available

**Question 10. What is the role of fluid resuscitation?**

Recommendation. Fluid resuscitation is recommended for patients with circulatory signs. Initially, 20 ml/kg of fluids may be given, and then the amount may be adjusted according to response. (Strong recommendation).

Summary of the evidence. We did not find any high-quality studies on the use of fluid resuscitation in anaphylaxis, nor did two systematic reviews (Dhami et al., 2014; Rubin et al., 2014). All eight clinical guidelines generally recommended fluid resuscitation as a first- or second-line treatment for patients with circulatory failure, with the recommended dose as around 20 ml/kg, which could be changed if appropriate (Supplementary Appendix S6).

Rationale. During anaphylaxis vascular permeability increased, which shifts intravascular fluid into the extravascular space, potentially leading to life-threatening circulatory collapse (Brown et al., 2004b; LoVerde et al., 2018). Fluid resuscitation helps maintain blood volume.

Comment: Extrapolating from evidence for other conditions, in most cases, crystalloid solutions are preferred over colloidal solutions, and sodium solution over sugar solution (Padhi et al., 2013; Perel et al., 2013).

Comment for emergency management of refractory anaphylaxis: The incidence of refractory anaphylaxis is reported to be 3–5% of patients [(Francuzik et al., 2018)]. For refractory anaphylaxis, noninvasive ventilation should be performed routinely and invasive ventilation should be performed when indicated (recommendation 6). Cardiopulmonary resuscitation should be prepared. Vasopressors combined with epinephrine may improve the outcome in in-hospital cardiac arrest patients [(Layek et al., 2014)]. so some guidelines suggest the use of vasopressor might be beneficial [(Lieberman et al., 2005; Soar et al., 2008; Lieberman et al., 2010; Simons et al., 2011; Campbell et al., 2014; Ring et al., 2014; Lieberman et al., 2015)]. Evidence did not support recommendations among different vasopressors [(Gamper et al., 2016)], so dopamine, norepinephrine and vasopressin can be titrated with careful monitor [(Lieberman et al., 2005; Soar et al., 2008; Lieberman et al., 2010; Simons et al., 2011; Campbell et al., 2014; Ring et al., 2014; Lieberman et al., 2015)]. Glucagon is pathophysiologically rational for anaphylaxis patients taking β-blockers but only case reports are available to support this [(Thomas and Crawford, 2005; Rukma, 2019)].

### Part 4. Post-emergency Management

**Question 11. How long should anaphylaxis patients be monitored after emergency treatment?**

Recommendation. After the relief of symptoms, anaphylaxis patients, especially those with hypotension, are recommended to be monitored for at least 12 h in the hospital, with heartrate, BP, respiration, oxygen saturation, and urine volume (Weak recommendation).

Summary of the evidence. One pre-post study revised a clinical pathway in a pediatric emergency department, recommending patients with a high risk of biphasic anaphylaxis (history of biphasic or severe reactions or asthma, progression of or persistent symptoms, hypotension, or requirement of >1 epinephrine dose or fluid bolus) to be admitted to the hospital, decreasing the recommended length of observation from 8 to 4 h for other anaphylaxis patients, and dispensing an epinephrine autoinjector with instructions when discharging patients; this revision was reported to reduce the hospitalization rate (RR 0.44, 95% CI 0.34–0.56) but increased the rate of 3-days allergy-related revisit though not significantly (RR 4.18, 95% CI 0.50–34.98), providing very-low quality evidence (Supplementary Table S17 in Supplementary Appendix S4) (Lee et al., 2018). Five clinical guidelines recommended 4–12 h of monitoring in uniphasic anaphylaxis, and up to several days of admission for severe or protracted anaphylaxis (Supplementary Appendix S6).

Rationale. The effect of the composite intervention cannot be entirely credited to the reduction in time of monitor (Lee et al., 2018). The high point estimation (RR 4.18) of the risk of 3-days allergy-related revisit in the post-intervention group also reduced our confidence on a short-time monitor (Lee et al., 2018). Besides, risk factors for biphasic anaphylaxis were supported by very low-quality evidence (Shaker et al., 2020), making it difficult to predict biphasic anaphylaxis. Additionally, the incidence of biphasic anaphylaxis occurs in 0.4–23.3% of cases, and the mean and median time to the second phase was 9.9 and 11 h after control of initial symptoms, respectively (Tole and Lieberman, 2007; Lee et al., 2015), suggesting the monitor time less than 12 h might not be sufficient. Unmonitored biphasic anaphylaxis may induce falling, traffic collisions, or other accidents, while these events were not reported in the pre-post study. Hence, it might be more beneficial to supervise patients with anaphylaxis for at least 12 h rather than a shorter time, especially in patients who develop hypotension.

Comment: The AAAAI/ACAAI guideline/systematic review reported that a more severe initial episode of anaphylaxis (OR 2.11, 95% CI 1.23–3.61), repeated epinephrine doses required with the initial onset (OR 4.82, 95% CI 2.70–8.58), wide pulse pressure (OR 2.11, 95% CI 1.32–3.37), cutaneous signs and symptoms (OR 2.54, 95% CI 1.25–5.15), drug trigger in children (OR 2.35, 95% CI 1.16–4.76) and unknown trigger (OR 1.63, 95% CI 1.14–2.33) might be associated with biphasic anaphylaxis (Cardona et al., 2020).

**Question 12. How should cases of drug-induced anaphylaxis be reported?**

Recommendation. All cases of drug-induced anaphylaxis should be reported to an ADR surveillance system. The report should contain information about suspected triggers, symptoms and their timing relative to the drug exposure, management steps, and clinical outcomes (Strong recommendation).

Summary of the evidence. Two guidelines encouraged the reporting of drug-induced anaphylaxis (Supplementary Appendix S6).

Rationale. Reporting drug-induced anaphylaxis cases can uncover potential safety signals that may help clinicians and regulatory agencies to make public safety warnings and policies (Inácio et al., 2017).

Comment. The Chinese website of adverse drug reaction surveillance system is located at: http://www.adrs.org.cn/.

**Question 13. How can anaphylaxis be prevented in short term?**

Recommendation. The key preventive measure is to avoid allergens. Prophylactic medications cannot be routinely used in the general population. Though some medications show the potential of reducing risk of anaphylaxis or related symptoms of drug-induced reactions, concerns remain about using them to prevent anaphylaxis (Table 6) (Weak recommendation).

**TABLE 6:** Summary of potential anaphylaxis preventive medication and their concerns.

Premedication	Effects	Concerns	Dosage reported to be effective in adults
Epinephrine	May reduce the risk of antivenom-induced anaphylaxis [(de Silva et al., 2011)] and of early adverse drug reactions when the baseline risk is high (>30%) (Habib, 2011)	Effect on all-cause anaphylaxis is unverified	0.25 mg, SC, immediately before exposure [(de Silva et al., 2011)]
		As a high alert medication, epinephrine commonly produces some adverse reactions and may cause severe adverse reaction. There is no antidote for epinephrine	
Two-dose glucocorticoids	Probably reduce the risk of ICM-induced cutaneous symptoms, respiratory symptoms and Grade 3^a^ reaction (Delaney et al., 2006; Tramã¨R et al., 2006)	Effect on all-cause anaphylaxis is unverified	Methylprednisolone 32 mg, PO, at 6–24 h and then 2 h in advance of exposure (Tramã¨R et al., 2006)
		Did not appear to significantly reduce ICM-induced hemodynamic symptoms due to a low baseline risk (Tramã¨R et al., 2006)	
		The number needed to treat to prevent one episode of a potentially life-threatening, ICM-related reaction was 100–150 patients (Tramã¨R et al., 2006)	
		May mask the early signs of a life-threatening anaphylaxis (Suresh et al., 2012)	
H_1_ antagonist	May reduce the risk of ICM-induced cutaneous symptoms (Tramã¨R et al., 2006) and anaphylactoid reactions (Delaney et al., 2006); may reduce the risk of anaphylaxis in patients experiencing allergic reactions [(Kawano et al., 2017b)]	Effect on all-cause anaphylaxis in unselected population is unverified	Chlorpheniramine 10 mg, SC, 15 min in advance of exposure (Delaney et al., 2006) Hydroxyzine 100 mg, PO, 12 h in advance of exposure (Delaney et al., 2006)
		Does not significantly reduce ICM-induced respiratory symptoms (Tramã¨R et al., 2006)	
		May mask the early signs of life-threatening anaphylaxis (Suresh et al., 2012)	

ICM, iodinated contrast media; PO, per orem (by mouth); SC, subcutaneous.

Findings provided by our systematic review are summarized in Supplementary Tables S13–S17 in Supplementary Appendix S4.

a GRADE, 3 ICM-induced adverse reaction is defined as various combinations of respiratory and hemodynamic symptoms.

Summary of the evidence. Comprehensive evidence provided by our systematic review is summarized in the SoF table (Supplementary Tables S13–S17 in Supplementary Appendix S4). No study was found that supports the efficacy of allergen avoidance. One cohort study reported that prophylactic use of H1a may reduce the risk of anaphylaxis (aOR 0.34, 95% CI 0.17–0.70, low-quality evidence). And severe anaphylaxis (RR 0.70, 95% CI 0.18 to 2.64, very low-quality evidence) among patients experiencing allergic reaction [(Kawano et al., 2017b)]. An RCT [(de Silva et al., 2011)] reported that epinephrine likely reduced the risk of antivenom-induced anaphylaxis (OR 0.85, 95% CI 0.71 to 1.00, low-quality evidence) and antivenom-reduced severe anaphylaxis (OR 0.62, 95% CI 0.51–0.74, moderate quality evidence). Two RCTs [(Fan et al., 1999; de Silva et al., 2011)] and one cohort study [(Caron et al., 2009)] reported uncertain findings for the use of glucocorticoids, H1a, or their combination for the prevention of anaphylaxis (very low-quality evidence). Because the evidence is indirect, it is not sufficiently robust for generalization to an unselected population.

One systematic review [(Habib, 2011)] found that epinephrine-containing premedication significantly reduced the risk of early adverse reactions to antivenom while non-epinephrine premedication did not. One high-quality systematic review [(Delaney et al., 2006)] indicated that H1a and a two-dose glucocorticoids regimen each significantly reduce the risk of anaphylactoid reactions caused by iodinated radiological contrast media (ICM). A systematic review [(Tramã¨R et al., 2006)] reported that H1a significantly reduced the risk of ICM-induced cutaneous symptoms and there was a nonsignificant reduction in respiratory symptoms. They also reviewed studies showing that glucocorticoids significantly reduce the risk of cutaneous symptoms and respiratory symptoms, with a nonsignificant reduction in the risk of hemodynamic symptoms. Finally, they noted that two-dose glucocorticoid regimens reduced the risk of Grade 1 and Grade 3 ICM-related reactions more than one-dose regimen, when two regimens showed equivalent effects on at Grade 2 reactions.

Seven guidelines noted that avoiding exposure to known allergens is the most effective preventive measure and that the effectiveness of drug prophylaxis is unknown (Supplementary Appendix S6).

Rationale. There is no direct evidence that avoidance of allergens reduces the risk of anaphylaxis, but it should theoretically. Nonetheless, it is difficult to completely avoid suspected triggers. With weak evidence and concerns (Table 6), the overall benefit of premedication in patients who cannot avoid a potential trigger is unclear. We cannot make a well-directed recommendation.

Comment. When prophylactic medication is needed, dosages of premedication reported to show beneficial effects are shown in Table 6. Two-dose glucocorticoids may perform better than one-dose glucocorticoids (Delaney et al., 2006; Tramã¨R et al., 2006; Cai et al., 2012).

Immunotherapy may prevent anaphylaxis in long-term anaphylaxis management, which should be performed with the supervision of experienced allergists or immunologists (Simons et al., 2011; Muraro et al., 2014).

**Question 14. What education should be provided to patients when they are discharged?**

Recommendation. At discharge, health care providers should teach patients and/or caregivers about anaphylaxis including: diagnostic criteria, avoidance of potential triggers, and first-line treatments. (Strong recommendation).

Summary of the evidence. Two systematic review (Choo and Sheikh, 2007; Nurmatov et al., 2008) did not identify any high-quality studies on the effect of patient education. Seven clinical guidelines recommended patient and caregiver education on the important aspects of anaphylaxis (Supplementary Appendix S6).

Rationale. One RCT reported that providing a 6 h comprehensive training course can reduce the anxiety of patients at-risk for anaphylaxis [(Brockow et al., 2015)]. In addition, information contained in the three learning objectives are thought to safeguard the safety of potential anaphylaxis patients [(Kroigaard et al., 2007; Harper et al., 2009; Simons et al., 2011; Muraro et al., 2014; Lieberman et al., 2015)].

### Dissemination and Implementation

This guideline is intended for publication in an open access journal. An interpretation in Chinese will be published and will be made available on websites of the medical societies participating the guideline development. The guideline has been introduced (Minutes of The 11th China, 2020; Zhai, 2019) at relevant academic meetings and more presentations are planned. Additionally, this guideline has been used to inform the update of the Chinese National Formulary, the Chinese Formulary of National Essential Medicines, and the drafting of the Chinese Guiding Principles for Skin Testing of β-lactam antibiotics. It will be used to inform the drafting of other Chinese regulations and professional reference books. These activities will facilitate the dissemination and implementation of this guideline. Surveys will be conducted to assess the dissemination and implementation of this guideline.

### Plans for Updating

We would update this guideline once we retrieve striking findings that would change the clinical practice obviously. Otherwise, we would update this guideline after five to 10 years.

## Discussion

In this first comprehensive anaphylaxis guideline that used GRADE framework, we recommended NIAID/FAAN diagnostic criteria and four-tier grading system for the diagnosis and elaborated on how to use epinephrine.

### Gaps in Knowledge

It is challenging to conduct research on the emergency management of anaphylaxis because it is difficult to predict and diagnose, and when it occurs, the onset and progression is rapid. Therefore, there are many knowledge gaps in the evidence. Real world evidence is greatly expanding in the age of modern technology and communications (Sherman et al., 2016). It is becoming easier to conduct multicenter trials (Paul et al., 2005) to generate evidence on managing anaphylaxis. From the perspective of this guideline, there are several gaps and that we hope can draw attention for future research:

1) Diagnostic criteria for anaphylaxis with high sensitivity and specificity and that are easy to implement by clinical workers.
2) Comparison of the safety and efficacy of IM epinephrine and monitored IV infusion epinephrine.
3) Use of nebulized epinephrine to decrease edema and obstruction in the upper airways.
4) Efficacy of inhaled β-2 agonist in managing edema of lower airway, bronchospasm, wheeze, and stridor.
5) Use of glucocorticoids and H1a improve secondary outcomes in anaphylaxis patients.

There is an RCT aimed verifying the efficacy of glucocorticoids in preventing biphasic anaphylaxis; it plans to enroll 210 participants and has been recruiting for 2 years (AL-ANSARIK, 2018). We call for more inter-center and international collaborations to generate evidence quickly and efficiently.

### Strengths

This guideline was the first guideline that used GRADE framework to present evidence and make recommendations for the comprehensive emergency management of anaphylaxis. In addition, healthcare providers that are engaged in different medical specialties posed candidate questions and the multidisciplinary panel voted for key questions, and we combined the evidence and experience of professionals from fifteen areas to formulate recommendations. These process made sure that this guideline meets the critical need of medical workers from various departments.

### Limitation

The majority of the evidence for this guideline was of low or very-low quality. Hence, we make these efforts: (1) we combined original evidence, experience of experts from around the world via other guidelines, and the experience of Chinese healthcare professionals; (2) we used a Delphi method, which helped create consensus by anonymous voting and feedback where there was little or no concrete evidence and when expert opinion was important (Eubank et al., 2016).

## Conclusion

For the emergency management of anaphylaxis we conclude that:

• NIAID/FAAN diagnostic criteria and four-tier grading system should be used for the diagnosis;
• Prompt and proper administration of epinephrine is critical;
• Robust evidence is needed for glucocorticoids and H1a in both the treatment and prevention of anaphylaxis.

We call for international collaborations to generate more robust evidence for managing anaphylaxis.

## Exclusive licences

The Corresponding Author has the right to grant on behalf of all authors and does grant on behalf of all authors, a worldwide licence to the Publishers and its licensees in perpetuity, in all forms, formats and media (whether known now or created in the future), to i) publish, reproduce, distribute, display and store the Contribution, ii) translate the Contribution into other languages, create adaptations, reprints, include within collections and create summaries, extracts and/or, abstracts of the Contribution, iii) create any other derivative work(s) based on the Contribution, iv) to exploit all subsidiary rights in the Contribution, v) the inclusion of electronic links from the Contribution to third party material where-ever it may be located; and, vi) licence any third party to do any or all of the above.

## Abbreviations

AAAAI, american academy of allergy, asthma and immunology; ACAAI, american college of allergy, asthma, and immunology; ADRs, adverse drug reactions; AGREE II, appraisal of guidelines for research and evaluation; aOR, adjusted odds ratio; AUC, area under the plasma concentration versus time curve; BP, blood pressure; Cmax, peak plasma concentrations; ED, emergency department; GRADE, grading of recommendations assessment, development, and evaluation; H1a, H1 antagonist; ICM, radiological contrast media; ICU, intensive unit care; IM, intramuscular or intramuscularly; IV, intravenous or intravenously; MCT, mast cell tryptase; NCMSA, national center for medical service administration; NIAID/FAAN, american national institute of allergy and infectious diseases and the food allergy and anaphylaxis network; OR, odds ratio; RCT, randomized controlled trials; RR, risk ratio; SC, subcutaneous or subcutaneously; SoF, summary of findings; Tmax, maximum plasma concentration.
