# Supplementary material for: A Clinical Practice Guideline for the Emergency Management of Anaphylaxis (2020)
Source: Front Pharmacol. 2022 Mar 28;13:845689. doi: 10.3389/fphar.2022.845689 (PMC8996305; doi:10.3389/fphar.2022.845689)
Supplement: Supplementary file 1 [file DataSheet1.docx]

1. Assembling guideline development project team

The NCMSA invited Drug-Induced Diseases Professional Committee of Chinese Pharmacological Society, Chinese College of Allergy and Asthma, Chinese Society of Allergy, Chinese Society for Emergency Medicine, Chinese Thoracic Society, Chinese Society of Anesthesiology, Hospital Pharmacy Committee of Chinese pharmaceutical Association, Chinese Nursing Association. Pharmacy Department of Peking University Third Hospital and Chinese GRADE Center to build the steering group. The steering group members invited representatives in 18 specialties to form the guideline Expert Advisory Group and the Guideline-Development Working group in July 2017.

Health care providers from a wide range of areas who are familiar with values and preferences of anaphylaxis patients participated in the guideline development, so we did not invite patients to derectly participate in the guideline development.

**Table 1. List of members of guideline development project team**

| **Name** | **Institution** | **Title** | **Specialty** |
| --- | --- | --- | --- |
| **The Steering Group in the panel^a^** | | | |
| **Rongchang Chen** | The First Affiliated Hospital Of Guangzhou Medical University | Professor | Respirology |
| **Yuguo Chen** | Qilu Hospital of Shandong University | Professor | Emergency medicine |
| **Tianzuo Li** | Beijing Shijitan Hospital, Capital Medical University | Professor | Anesthesiology |
| **Qiang Wang** | National Center for Medical Service Administration, National Health Commission of the People’s Republic of China | Professor | Government representative |
| **Yuqin Wang** | Xuanwu Hospital of Capital Medical University | Professor | Pharmacology |
| **Kehu Yang** | Evidence-based Medicine Center of Lanzhou University, Chinese GRADE Center | Professor | Evidence-based medicine |
| **Jia Yin** | Peking Union Medical College Hospital | Professor | Allergology |
| **Suodi Zhai** | Peking University Third Hospital  Drug Evaluation of Peking University Health Science Center | Professor | Pharmacy |
| **Hongjun Zhang** | Peking University Third Hospital | Professor | Nursing |
| **Other panel members^b^** | | | |
| **Yaolong Chen** | Evidence-based Medicine Center of Lanzhou University, Chinese GRADE Center | Professor | Evidence-based medicine |
| **Hailong Dong** | Xijing Hospital, The Fourth Military Medical University; | Professor | Anesthesiology |
| **Qinglong Gu** | Children's Hospital Affiliated to Capital Institute of Pediatrics | Professor | Pediatry |
| **Daihong Guo** | The Chinese PLA General Hospital | Professor | Pharmacy |
| **Xuehui Hu** | Xijing Hospital, The Fourth Military Medical University | Professor | Nursing |
| **Lixin Xie** | The Chinese PLA General Hospital | Professor | Respirology |
| **Baohua Li** | Peking University Third Hospital | Professor | Nursing |
| **Yuzhen Li** | Peking University People’s Hospital | Professor | Pharmacy |
| **Tongyu Lin** | Sun Yat-sen University Cancer Center | Professor | Oncology |
| **Fang Liu** | Committee of Hospital Pharmacy, Chinese Pharmaceutical Association  Drug Evaluation of Peking University Health Science Center | Chief pharmacist | Pharmacy |
| **Zhiqiang Liu** | Shanghai First Maternity and Infant Hospital | Professor | Anesthesiology |
| **Lanting Lyu** | Health Technology Assessment and Health Policy Research Group at Renmin University | Associate professor | Health economics |
| **Qingbian Ma** | Peking University Third Hospital | Chief physician | Emergency medicine |
| **Quanxi Mei** | Shenzhen Bao’an Pure Chinese Medicine Treatment Hospital | Professor | Traditional Chinese Medicine |
| **Jie Shao** | Ruijin Hospital, Shanghai Jiaotong University School of Medicine | Chief physician | Allergology |
| **Yida Tang** | Fuwai Hospital, Chinese Academy of Medical Sciences and Peking Union Medical College | Professor | Cardiology |
| **Huawen Xin** | General Hospital of Central Theater Command of PLA | Professor | Pharmacy |
| **Fan Yang** | Huashan Hospital, Fudan University | Chief physician | Infectious disease |
| **Hui Yang** | The First Hospital of Shanxi Medical University | Professor | Nursing |
| **Wanhua Yang** | Ruijin Hospital, Shanghai Jiaotong University School of Medicine | Professor | Pharmacy |
| **Xu Yao** | Institute of Dermatology and Hospital for Skin Diseases, Chinese Academy of Medical Sciences & Peking Union Medical Collage | Professor | Allergology |
| **Chunshui Yu** | Tianjin Medical University General Hospital | Professor | Radiology |
| **Siyan Zhan** | School of Public, Peking University | Professor | Epidemiology |
| **Guoqiang Zhang** | China-Japan Friendship Hospital | Professor | Emergency medicine |
| **Ya’an Zheng** | Peking University Third Hospital | Chief physician | Emergency medicine |
| External review group leader^c^ | | | |
| Qingbian Ma | Peking University Third Hospital | Chief physician | Emergency medicine |
| Ya’an Zheng | Peking University Third Hospital | Chief physician | Emergency medicine |
| Minggui Wang | Huashan Hospital, Fudan University | Professor | Infectious disease |
| Zhu Zhu | Peking Union Medical College Hospital | Professor | Pharmacy |
| Baoguo Zhou | The First Affiliated Hospital of Harbin Medical University | Associate professor | Surgery |
| The Guideline-Development Working group^d^ | | | |
| Chang Cui | Peking University Third Hospital  Drug Evaluation of Peking University Health Science Center | Master Candidate | Evidence-based medicine |
| Shuhua Deng | Peking University Third Hospital | Senior nurse | Nursing |
| Jianqing Gu | Peking Union Medical College Hospital | Attending physician | Allergology |
| Chao Huang | National Center for Medical Service Administration, National Health Commission of the People’s Republic of China | Associate professor | Government representative |
| Lisha Li | Peking Union Medical College Hospital | Attending physician | Allergology |
| Xiaotong Li | Peking University Third Hospital  Drug Evaluation of Peking University Health Science Center | Master | Evidence-based medicine |
| Pengfei Liu | Beijing Shijitan Hospital, Capital Medical University | Attending physician | Anesthesiology |
| Yuan Lyu | Qilu Hospital of Shandong University | Attending physician | Emergency medicine |
| Zhengqian Li | Peking University Third Hospital | Attending physician | Anesthesiology |
| Xiang Ma | Peking University Third Hospital  Drug Evaluation of Peking University Health Science Center | PhD candidate | Allergology |
| Peng Men | Peking University Third Hospital  Drug Evaluation of Peking University Health Science Center | PhD candidate | Evidence-based medicine |
| Chunli Shao | Fuwai Hospital, Chinese Academy of Medical Sciences & Peking Union Medical College | Associate chief physician | Cardiology |
| Kuan Wang | The Chinese People’s Liberation Army General Hospital | Associate chief physician | Respirology |
| Sai Wang | Xuanwu Hospital of Capital Medical University | Senior pharmacist | Pharmacology |
| Mo Xian | The First Affiliated Hospital Of Guangzhou Medical University | Attending Physician | Respirology |
| Yajuan Xiong | Peking University Health Science Center | Master candidate | Pharmacy |
| Hangci Zheng | Peking University Third Hospital  Drug Evaluation of Peking University Health Science Center | Master | Pharmacy |
| Pengxiang Zhou | Peking University Third Hospital  Drug Evaluation of Peking University Health Science Center | Master | Evidence-based medicine |

GRADE: Chinese Grading of Recommendations Assessment，Development and Evaluation (GRADE) Center.

^a^ Responsibilities of the steering group: identify the topic and scope of the guideline; set up the guideline expert advisory group, guideline-development working group and the external review group; manage the conflict of interest; supervise the drafting of the protocol and approve it; pose and confirm clinical questions and outcomes; supervise the forming of evidence; reach consensus on recommendations; examine and approve the external review; revise and approve the guideline.

^b^ Responsibilities of the guideline expert advisory group: pose and confirm clinical questions and outcomes; supervise the forming of evidence; reach consensus on recommendations; revise the guideline.

^c^ Responsibilities of the external review group: evaluate the scope of guideline and clinical questions; evaluate the accuracy, clarity and feasibility of recommendations; make suggestions for revision.

^d^ Responsibilities of the guideline-development working group: draft the protocol; pose and collect clinical questions and outcomes; form and evaluate evidence; conduct external reviews, draft the guideline.

1. Timeline of the guideline development


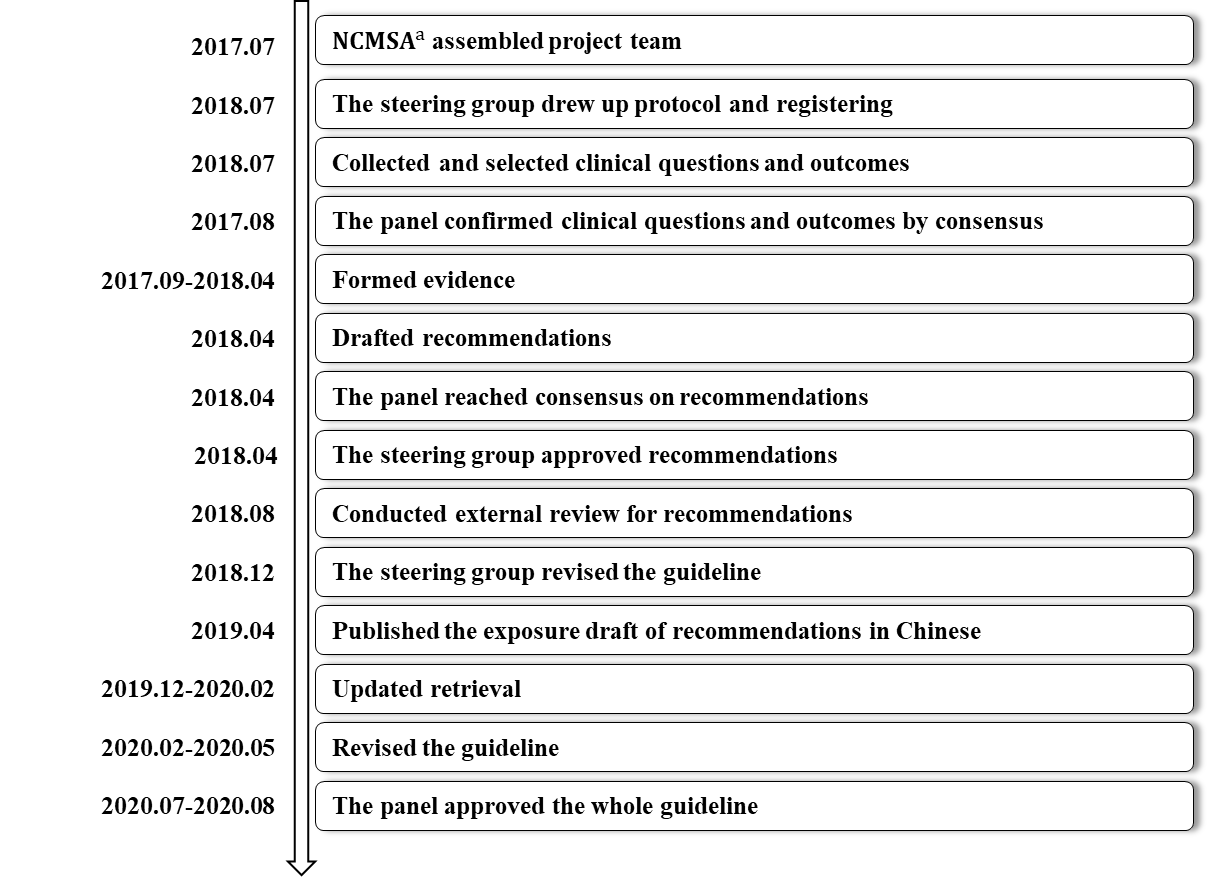


^a^ the National Center for Medical Service Administration (NCMSA) of National Health Commission of the People’s Republic of China

**Figure 1. Timeline of the guideline development**

1. Search stratagy

As mentioned in the manuscript, all 25 clinical questions focused on the same population, so we used one strategy to identify articles with specific population and study types. Besides, we checked reference lists of included studies to identify potentially relevant studies.

**Search strategy for PubMed**

1 Anaphylaxis[Mesh] OR Anaphylaxis[All fields] OR (anaphylactic react*)[TW] OR (anaphylactic shock*)[TW] OR (anaphylactic syndrome*)[TW] OR (anaphylactoid react*)[TW] OR (anaphylactoid shock*)[TW] OR (anaphylactoid syndrome*)[TW] OR (acute systemic allergic react*)[TW]

2 animals[mh] NOT humans [mh]

3 2 not 1

4 Clinical pathway[mh] OR Clinical protocol[mh] OR Consensus[mh] OR Consensus development conferences as topic[mh] OR Critical pathways[mh] OR Guidelines as topic [Mesh:NoExp] OR Practice guidelines as topic[mh] OR Health planning guidelines[mh] OR guideline[pt] OR practice guideline[pt] OR consensus development conference[pt] OR consensus development conference, NIH[pt] OR position statement*[tiab] OR policy statement*[tiab] OR practice parameter*[tiab] OR best practice*[tiab] OR standards[ti] OR guideline[ti] OR guidelines[ti] OR ((practice[tiab] OR treatment*[tiab]) AND guideline*[tiab]) OR CPG[tiab] OR CPGs[tiab] OR consensus*[tiab] OR ((critical[tiab] OR clinical[tiab] OR practice[tiab]) AND (path[tiab] OR paths[tiab] OR pathway[tiab] OR pathways[tiab] OR protocol*[tiab])) OR recommendat*[ti] OR (care[tiab] AND (standard[tiab] OR path[tiab] OR paths[tiab] OR pathway[tiab] OR pathways[tiab] OR map[tiab] OR maps[tiab] OR plan[tiab] OR plans[tiab])) OR (algorithm*[tiab] AND (screening[tiab] OR examination[tiab] OR test[tiab] OR tested[tiab] OR testing[tiab] OR assessment*[tiab] OR diagnosis[tiab] OR diagnoses[tiab] OR diagnosed[tiab] OR diagnosing[tiab])) OR (algorithm*[tiab] AND (pharmacotherap*[tiab] OR chemotherap*[tiab] OR chemotreatment*[tiab] OR therap*[tiab] OR treatment*[tiab] OR intervention*[tiab]))

5 systematic[sb] OR meta-analysis[pt] OR meta-analysis as topic[mh] OR meta-analysis[mh] OR meta analy*[tw] OR metanaly*[tw] OR metaanaly*[tw] OR met analy*[tw] OR integrative research[tiab] OR integrative review*[tiab] OR integrative overview*[tiab] OR research integration*[tiab] OR research overview*[tiab] OR collaborative review*[tiab] OR collaborative overview*[tiab] OR systematic review*[tiab] OR technology assessment*[tiab] OR technology overview*[tiab] OR "Technology Assessment, Biomedical"[mh] OR HTA[tiab] OR HTAs[tiab] OR comparative efficacy[tiab] OR comparative effectiveness[tiab] OR outcomes research[tiab] OR indirect comparison*[tiab] OR ((indirect treatment[tiab] OR mixed-treatment[tiab]) AND comparison*[tiab]) OR Embase*[tiab] OR Cinahl*[tiab] OR systematic overview*[tiab] OR methodological overview*[tiab] OR methodologic overview*[tiab] OR methodological review*[tiab] OR methodologic review*[tiab] OR quantitative review*[tiab] OR quantitative overview*[tiab] OR quantitative synthes*[tiab] OR pooled analy*[tiab] OR Cochrane[tiab] OR Medline[tiab] OR Pubmed[tiab] OR Medlars[tiab] OR handsearch*[tiab] OR hand search*[tiab] OR meta-regression*[tiab] OR metaregression*[tiab] OR data synthes*[tiab] OR data extraction[tiab] OR data abstraction*[tiab] OR mantel haenszel[tiab] OR peto[tiab] OR der-simonian[tiab] OR dersimonian[tiab] OR fixed effect*[tiab] OR "Cochrane Database Syst Rev"[Journal:__jrid21711] OR "health technology assessment winchester, england"[Journal] OR "Evid Rep Technol Assess (Full Rep)"[Journal] OR "Evid Rep Technol Assess (Summ)"[Journal] OR "Int J Technol Assess Health Care"[Journal] OR "GMS Health Technol Assess"[Journal] OR "Health Technol Assess (Rockv)"[Journal] OR "Health Technol Assess Rep"[Journal]

6 randomized controlled trial[pt] OR controlled clinical trial[pt] OR randomized[tiab] OR placebo[tiab] OR clinical trials as topic[mesh:noexp] OR randomly[tiab] OR trial[ti] NOT (animals[mh] NOT humans [mh])

7 (“Case-Control Studies”[Mesh] OR "retrospective studies"[mesh] OR “Control Groups”[Mesh] OR “Matched-Pair Analysis”[mesh] OR (case[TIAB] AND control[TIAB]) OR (cases[TIAB] AND controls[TIAB]) OR (cases[TIAB] AND controlled[TIAB]) OR (case[TIAB] AND comparison*[TIAB]) OR (cases[TIAB] AND comparison*[TIAB]) OR “control group”[TIAB] OR “control groups”[TIAB]) OR (cohort studies[mesh] OR longitudinal studies[mesh] OR follow-up studies[mesh] OR prospective studies[mesh] OR retrospective studies[mesh] OR cohort[TIAB] OR  prospective[TIAB] OR retrospective[TIAB]) OR (Cross-Sectional Studies[Mesh] OR cross-sectional[TIAB] OR Prevalence[mesh] OR prevalence[tiab] OR transversal study[tiab]) OR (Incidence[mesh] OR incidence[tiab]) OR “Epidemiologic Studies”[Mesh]

8 (case series[tw]) OR (cases and series[tw])

9 economics[Subheading:noexp] OR cost[Title/Abstract] OR health care costs[MeSH:noexp] OR randomized controlled trial[Publication Type] OR cost effectiveness[Title/Abstract] OR cost effective[Title/Abstract]

10 3 and (or/4-9)

11 limit 11 by filters: Publication date from 1990/01/01

**Search strategy for Embase.com**

1 'anaphylaxis'/exp OR anaphylaxis OR (anaphylactic AND react*) OR (anaphylactic AND shock*) OR (anaphylactic AND syndrome*) OR (anaphylactoid AND react*) OR (anaphylactoid AND shock*) OR (anaphylactoid AND syndrome*) OR (acute AND systemic AND allergic AND react*)

2 [animals]/lim NOT [humans]/lim

3 1 not 2

4 [chinese]/lim OR [english]/lim

5 3 AND 4

6 (‘clinical pathway’/exp OR ‘clinical pathway’) OR (‘clinical protocol’/exp OR ‘clinical protocol’) OR (‘ consensus’/exp OR consensus) OR (‘consensus development conference’/exp OR ‘consensus development conference’) OR (‘consensus development conferences as topic’/exp OR ‘consensus development conferences as topic’) OR ‘critical pathways’/de OR (‘guideline’/exp OR guideline) OR ‘guidelines as topic’/de OR (‘practice guideline’/exp OR ‘practice guideline’) OR ‘practice guidelines as topic’/de OR ‘health planning guidelines’/de OR (‘treatment guidelines’/exp OR ‘treatment guidelines’) OR ('guideline'/de or 'practice guideline'/de or 'consensus development conference'/de or 'consensus development conference, NIH'/de) OR (((position statement*) or (policy statement*) or (practice parameter*) or (best practice*)):ti,ab,kw) OR ( (standards or guideline or guidelines):ti,kw) OR (((practice or treatment* or clinical) near guideline*):ti,ab) OR ((CPG or CPGs):ti,ab) OR (consensus*:ti,ab,kw) OR (((critical or clinical or practice) near/2 (path or paths or pathway or pathways or protocol*)):ti,ab,kw) OR (recommendat*:ti,kw) OR ((care near/2 (standard or path or paths or pathway or pathways or map or maps or plan or plans)):ti,ab,kw) OR ((algorithm* near/2 (screening or examination or test or tested or testing or assessment* or diagnosis or diagnoses or diagnosed or diagnosing)):ti,ab,kw) OR ((algorithm* near/2 (pharmacotherap* or chemotherap* or chemotreatment* or therap* or treatment* or intervention*)):ti,ab,kw)

7 'meta-analysis'/de OR ‘meta-analysis’/de OR ‘systematic review’/de OR 'meta-analysis as topic'/de OR 'systematic review as topic'/de OR (‘biomedical technology assessment’/exp OR ‘biomedical technology assessment’) OR (((systematic* near/3 (review* or overview*)) or (methodologic* near/3 (review* or overview*))):ti,ab,kw) OR (((quantitative near/3 (review* or overview* or synthes*)) or (research near/3 (integrati* or overview*))):ti,ab,kw) OR (((integrative near/3 (review* or overview*)) or (collaborative near/3 (review* or overview*)) or (pool* near/3 analy*)):ti,ab,kw) OR ((data synthes* or data extraction* or data abstraction*):ti,ab,kw) OR ((handsearch* or hand search*):ti,ab,kw) OR ((mantel haenszel or peto or der simonian or dersimonian or fixed effect* or latin square*):ti,ab,kw) OR ((met analy* or metanaly* or technology assessment* or HTA or HTAs or technology overview* or technology appraisal*):ti,ab,kw) OR ((meta regression* or metaregression*):ti,ab,kw) OR (meta-analy* or metaanaly* or systematic review* or biomedical technology assessment* or bio-medical technology assessment*) OR ((medline or cochrane or pubmed or medlars or embase or cinahl):ti,ab) OR ('health technology assessment reports':jt OR 'health technology assessment':jt OR 'gms health technology assessment':jt OR 'international journal of technology assessment in health care':jt OR 'evidence report/technology assessment':jt OR 'health technology assessment winchester, england':jt OR 'cochrane database of systematic reviews':jt) OR ((comparative near/3 (efficacy or effectiveness)):ti,ab,kw) OR ((outcomes research or relative effectiveness):ti,ab,kw) OR (((indirect or indirect treatment or mixed-treatment) near comparison*):ti,ab,kw)

8  'clinical trial'/de OR 'randomized controlled trial'/de OR 'randomization'/de OR 'single blind procedure'/de OR 'double blind procedure'/de OR 'crossover procedure'/de OR 'placebo'/de OR 'prospective study'/de OR ('randomi?ed controlled' NEXT/1 trial*) OR rct OR 'randomly allocated' OR 'allocated randomly' OR 'random allocation' OR (allocated NEAR/2 random) OR (single NEXT/1 blind*) OR (double NEXT/1 blind*) OR ((treble OR triple) NEAR/1 blind*) OR placebo*

9  ‘Case-Control Studies’/de OR ‘Control Groups’/de OR ‘Matched-Pair Analysis’/de OR ‘retrospective studies’/de OR ((case* near/5 control*) or (case near/3 comparison*) or control group*):ti,ab OR ‘cohort studies’/de OR ‘longitudinal studies’/de OR ‘follow-up studies’/de OR ‘prospective studies’/de OR ‘retrospective studies’/de OR cohortti,ab or longitudinalti,ab or prospectiveti,ab or retrospectiveti,ab OR ‘Cross-Sectional Studies’/de OR ‘Prevalence’/de OR (cross-sectional or prevalence or transversal):ti,ab,kw OR ‘Epidemiologic Studies’/de OR ‘Incidence’/de or incidence:ti,ab,kw

10 (case* series)

11 ‘cost effectiveness analysis’/de OR ‘randomized’ OR ‘economic’

12 5 and (or/6-11)

13 limit 12 by filters: Publication date from 1990/01/01

**Search strategy for Web of science**

1 TS=(Anaphylxis OR anaphylactic react* OR anaphylactic shock* OR anaphylactic syndrome* OR anaphylactoid react* OR anaphylactoid shock* OR anaphylactoid syndrome* OR acute systemic allergic react*)

2 TS=(rat or rats or cow or cows or chicken* or horse or horses or mice or mouse or bovine or animal)

3 TS=human

4 1 not (2 not 3)

6 Language: (English)

7 4 and 5

8 limit 7 by filters: Publication date from 1990/01/01

**Search strategy for Cochrane**

1. MeSH descriptor: [Anaphylaxis] explode all trees
2. anaphylaxis:ti,ab,kw (Word variations have been searched)
3. anaphylactic:ti,ab,kw (Word variations have been searched)
4. anaphylactoid:ti,ab,kw (Word variations have been searched)
5. 1 OR 2 OR 3 OR 4

**Search strategy for China National Knowledge Internet database** (http://www.cnki.net)

1 SU='严重'*('过敏'+'变态反应')+ '休克'*'过敏'

2 TI= '严重'*('过敏'+'变态反应')+ '休克'*'过敏'

3 AB= '严重'*('过敏'+'变态反应')+ '休克'*'过敏'

4 KY= '严重'*('过敏'+'变态反应')+ '休克'*'过敏'

5 1 or 2 or 3 or 4

1. limit 5 by filters: Publication date from 1990/01/01

**Search strategy for Wanfang database** (http://www.med.wanfangdata.com.cn)

1 (主题:休克 * 主题:过敏) + (主题:严重 * 主题:变态反应) + (主题:严重 * 主题:过敏)

2 (摘要:休克 * 摘要:过敏) + (摘要:严重 * 摘要:变态反应) + (摘要:严重 * 摘要:过敏)

3 (题名或关键词:休克 * 题名或关键词:过敏) + (题名或关键词:严重 * 题名或关键词:变态反应) + (题名或关键词:严重 * 题名或关键词:过敏)

4 1 or 2 or 3

5 主题:动物 + 主题:兔 + 主题:鼠 + 主题:猪 + 主题:马 + 主题:鸡 + 主题:狗

6 4 not 5

7 limit 6 by filters: Publication date from 1990/01/01

**Search strategy for Sinomed** (http://www.sinomed.ac.cn/).

1 "过敏"[常用字段:智能]) AND "休克"[常用字段:智能]

2 "严重"[常用字段:智能]) AND "变态反应"[常用字段:智能]

3 "过敏"[常用字段:智能]) AND "严重"[常用字段:智能]

4 1 or 2 or 3

5 体外研究[特征词]

6 4 not 5

7 核心期刊[文献来源]

8 6 and 7

9 limit 8 by filters: Publication date from 1990/01/01

**Search strategy for The National Institute for Health and Care Excellence**

1. anaphylaxis
2. anaphylactic
3. anaphylactoid
4. 1 or 2 or 4

**Search strategy for National Guideline Clearinghouse (NGC)**

The NGC was shut down at July 18, 2018, so the NGC was retrieved up to June 2018.

1. anaphylaxis
2. anaphylactic
3. anaphylactoid
4. 1 or 2 or 4

**Search strategy for Scottish Intercollegiate Guidelines Network**

1. anaphylaxis
2. anaphylactic
3. anaphylactoid
4. 1 or 2 or 4

**Search strategy for CMA Infobase: Clinical Practice Guidelines Database**

1. anaphylaxis
2. anaphylactic
3. anaphylactoid
4. 1 or 2 or 4

**Search strategy for Clinicaltrials.gov**

1. Conditions: anaphylaxis
2. Conditions: anaphylactic
3. Conditions: anaphylactoid
4. 1 or 2 or 3
5. Summary of Primary Evidence

Using the search strategy in Appendix 3 to retrieve literature available from 1990 to December 2019, we did not find original studies for question 2, 3, 4, 5, 6.4, 6.7, 6.9, 6.11, 6.12, 8, 10, 12 and 14. For remaining questions, original evidence founded by the systematical literature retrieval for **each** clinical question was summarized using GRADE^[1,2]^. The summary of finding (SoF) tables list the considerations for rating the quality of evidence, the relative and absolute changes in effect of the study outcome, as well as the level of certainty supported by the evidence.

For original evidence, outcomes of interest were rated for importance by consensus (see Appendix 8). For each outcome, we included the highest-quality body of evidence. For example, if evidence for an outcome of moderate-quality consisted of low-risk cohort studies, and evidence of low-quality consisted of high-risk case-control studies, then they cannot be pooled. Thus, we only include the former body of evidence. We considered safety events less susceptible to prognostic factors than efficacy evidence, thus we rated the evidence for adverse effects down less when considering the risk of bias adjusting for prognostic factors.

## Table 1 Summary of quality of original evidence by using GRADE

| **Recommendation** | **Quality of Evidence by using GRADE** | **Notes** |
| --- | --- | --- |
| 1. Diagnosis | High to moderate (Table 2, only for NIAID/FAAN criteria) | For mast cell tryptase level and histamine level, only low-quality studies were found so we included these low-quality studies. But since different studies reported different thresholds, sensitivity and specificity of laboratory tests can not be poolded together. Hence, GRADE system is not applicable for them. Details could be seen in systematic review^[1]^. |
| 1. Grading | Very low (no primary study have been found) |  |
| 1. Non-professional preparation | Very low (no primary study have been found) |  |
| 1. Emergency monitor | Very low (no primary study have been found) |  |
| 1. Artificial airways | Very low (no primary study have been found) |  |
| 6.1 Status of epinephrine | High to very low (Table 3) |  |
| 6.2 Timing of epinephrine | Moderate to very low (Table 4) |  |
| 6.3 Status of I.M. epinephrine | Moderate to very low (Table 5-8) |  |
| 6.4 Dosage of I.M. epinephrine | Very low (no primary study have been found) |  |
| 6.5 Site of I.M. epinephrine | Low to very low (Table 9) |  |
| 6.6 Status of I.V. bolus epinephrine | Low to very low (Table 6, 7) |  |
| 6.7 Dosage of I.V. bolus epinephrine | Very low (no primary study have been found) |  |
| 6.8 Status of I.V. infusion epinephrine | Low to very low (Table 6, 8) |  |
| 6.9 Dosage of I.V. infusion epinephrine | Very low (no primary study have been found) |  |
| 6.10 Status of S.C. epinephrine | Moderate to very low (Table 5) |  |
| 6.11 Contraindication of epinephrine | Very low (no primary study have been found) |  |
| 6.12 Reduce ADR^a^ of epinephrine | Very low (no primary study have been found) |  |
| 7. H1a | Very low (Table 10) |  |
| 8. β2-agonist | Very low (no primary study have been found) |  |
| 9. Glucocorticoids | Low to very low (Table 11) |  |
| 10. Fluid resuscitation | Very low (no primary study have been found) |  |
| 11. Monitor duration | Very low (Table 12) |  |
| 12. Report drug-induced anaphylaxis | Very low (no primary study have been found) |  |
| 13. Prophylactic intervention | Moderate to very low (Table 13-16) |  |
| 14. Patient education | Very low (no primary study have been found) |  |

NIAID/FAAN: American National Institute of Allergy and Infectious Diseases and the Food Allergy and Anaphylaxis Network

ADR: adverse drug reaction

## Table 2. GRADE summary of findings: American National Institute of Allergy and Infectious Disease and the Food Allergy and Anaphylaxis Network (NIAID/FAAN) criteria in diagnosing anaphylaxis

The result of sensitivity analysis excluding one study with high risk of bias in our syetematic review was reported as the evidence^[3]^, because high-quality evidence can be more convicing.

| Summary accuracy | Sensitivity: 96 (95% CI 91 to 99), specificity: 77 (95% CI 72 to 82); diagnostic odds ratio 74.29 (95% CI 26.11 to 211.41)  Positive likelihood ratio: 4.19 (95% CI 2.51 to 6.99); Negative likelihood ratio:0.06 (95% CI 0.02 to 0.13) | | | | | |
| --- | --- | --- | --- | --- | --- | --- |
| Prevalence | 30%, 50%, 70% | | | | | |
| Outcome | No of participants (studies) | Results per 1000 patients tested (95% CI) | | | Certainty/Quality of evidence | Plain languages summary |
|  |  | pre-test probability of 30% | pre-test probability of 50% | pre-test probability of 70% |  |  |
| True positives | 388 (2) | 288 (273 to 297) | 480 (455 to 496) | 672 (637 to 693) | High Ꚛ Ꚛ Ꚛ Ꚛ | We are very certain that the NIAID/FAAN criteria identify 96% anaphylaxis patients |
| False negatives |  | 12 (3 to 27) | 20 (5 to 45) | 28 (7 to 63) |  |  |
| True negatives |  | 539 (504 to 574) | 385 (360 to 410) | 231 (216 to 246) | Moderate Ꚛ Ꚛ Ꚛ O  (serious inconsistency)^a^ | The NIAID/FAAN criteria probably identify 77% non-anaphylaxis patients |
| False positives |  | 161 (126 to 196) | 115 (90 to 140) | 69 (54 to 84) |  |  |

NIAID/FAAN: American National Institute of Allergy and Infectious Disease and the Food Allergy and Anaphylaxis Network.

^a^ Diagnostic accuracy studies started at high certainty/quality of evidence. Additionally, we rated it down one level for in inconsistency (substantial I^2^ as 79.7%).

| Table 3. GRADE summary of findings: Epinephrine in anaphylaxis | | | | | | |
| --- | --- | --- | --- | --- | --- | --- |
| **Outcomes** | **No of participants (studies)** | **Relative effects** | **Absolute effect estimates** | | **Certainty/Quality of evidence** | **Plain languages summary** |
|  |  |  | **Baseline risk for control group (per 1000)** | **Difference (95% CI) (per 1000)** |  |  |
| Hospitalization | 234  (1 cohort study) | Pre-ED epinephrine vs in-ED epinephrine:  aOR 0.25 (95% CI 0.10 to 0.62) | 170 | -122. (-150 to -87) | High Ꚛ Ꚛ Ꚛ Ꚛ  (Large magnitude of an effect, plausible residual confounding)^a^ | We are very certain of the effect of epinephrine on hospitalization. |
| ICU admission | 364  (1 case control study) | Pre-ED epinephrine vs non epinephrine:  RR 1.75 (95% CI 0.54 to 5.53)^b^ | 23 | 17 (-11 to 104) | Very low Ꚛ O O O  (Very serious risk of bias and imprecision)^c^ | We are very uncertain of the effect of epinephrine on ICU admission. |

|  | 213  (1 cohort study) | RR 2.08 (95% CI 0.27 to 16.2) | 23 | 25 (-17 to 350) | Very low Ꚛ O O O  (Very serious risk of bias and imprecision)^c^ |  |
| --- | --- | --- | --- | --- | --- | --- |

| Median length of ED stay | 234  (1 cohort study) | Pre-ED epinephrine vs in-ED epinephrine= 3 hours vs 4 hours, P=0.003 | 4 hours | Not available^d^ | Moderate Ꚛ Ꚛ Ꚛ O  (Plausible residual confounding)^e^ | Epinephrine probably reduce the length of ED stay |
| --- | --- | --- | --- | --- | --- | --- |
| ≥2 doses of epinephrine | 965  (1 case control study) | Pre-ED epinephrine vs non pre-ED epinephrine:  aOR 0.25 (95% CI 0.04 to 0.60) | 45 | -34 (-43 to -18) | High Ꚛ Ꚛ Ꚛ Ꚛ  (Large magnitude of an effect, plausible residual confounding)^f^ | We are very certain of the effect of epinephrine on the risk of ≥2 doses of epinephrine |
| ED intravenous fluid | 3498  (1 case control study) | Prehospital epinephrine vs non prehospital epinephrine:  aOR 1.00 (95% CI 0.83 to 1.47) | Not available^g^ | Not available^g^ | Very low Ꚛ O O O  (Very serious imprecision)^c^ | We are very uncertain of the effect of epinephrine on ED intravenous fluid. |
| ≥ 3 ED interventions^h^ | 123  (a cohort study) | RR 1.56 (95% CI 1.12 to 2.19) | 444 | 249 (53 to 528) | Very low Ꚛ O O O  (Very serious risk of bias)^h^ | We are very uncertain of the effect of epinephrine on multiple ED interventions^h^. |
| Subsequent in-ED hypotension | 340  (1 case control study) | aOR 0.25 (95% CI 0.09 to 0.71) | 150 | -112 (-43 to -136) | Moderate Ꚛ Ꚛ Ꚛ O  (Large magnitude of an effect)^i^ | Epinephrine probably reduce the risk of subsequent in-ED hypotension |
|  |  |  |  |  |  |  |
| Biphasic anaphylaxis | 3315  (10 case control studies) | OR 0.90 (95% CI 0.53 to 1.52) | Not available^g^ | Not available^g^ | Very low Ꚛ O O O  (Very serious risk of bias and imprecision, serious inconsistency)^j^ | We are very uncertain of the effect of epinephrine on biphasic anaphylaxis. |

More details about included studies for this clinical question could be seen in the systematic review^[2]^.

ED: emergency department; NA: not available.

^a^ Observational studies started at low certainty/quality of evidence. Then we rated it up two levels: one for large magnitude of the effect (aOR=0.25 [95% CI 0.10 to 0.62] and crude RR=0.38 [(95% CI 0.25 to 0.60]), and one for effect of plausible confounding (early use vs late use, rather than use vs nonuse), while we did not find factors that can seriously reduce the quality of evidence.

^b^ Original reported the OR of comparing no pre-ED epinephrine to pre-ED epinephrine as 0.56 (95% CI 0.17 to1.87). We converted it to the RR of comparing pre-ED epinephrine to no pre-ED epinephrine so the table would be easier to understand.

^c^ Observational studies started at low certainty/quality of evidence. Then we rated it down for very serious risk of bias (unadjusted analysis) and imprecision (very wide confidence interval).

^d^ Length of ED stay was presented as median and interquartile range, so difference could not be calculated.

^e^ Observational studies started at low certainty/quality of evidence. Then we rated it up one level for effect of plausible confounding (early use vs late use, rather than use vs nonuse), while we did not find factors that can seriously reduce the quality of evidence.

^f^ Observational studies started at low certainty/quality of evidence. Then we rated it up two levels: one for large magnitude of the effect (aOR=0.25 [95% CI 0.04 to 0.60], RR was not availabel), and one for effect of plausible confounding (early use vs late use and nonuse), while we did not find factors that can seriously reduce the quality of evidence.

^g^ Baseline was not reported, and no study reported this outcome among anaphylaxis patients who didn’t receive epinephrine, so baseline risk and difference were not available here.

^h^ Observational studies started at low certainty/quality of evidence. Then we rated it down for very serious risk of bias (unadjusted analysis)

^i^ Observational studies started at low certainty/quality of evidence. Then we rated it up one level for large magnitude of the effect (aOR=0.25 [95% CI 0.09 to 0.71], and crude RR=0.37 [(95% CI 0.16 to 0.86]).

^j^ Observational studies started at low certainty/quality of evidence. Then we rated it down for very serious risk of bias (unadjusted analysis) and imprecision (very wide confidence interval), serious inconsistency (substantial I^2^ as 58%).

| Table 4. GRADE summary of findings: Timing of epinephrine in anaphylaxis | | | | | | | | |
| --- | --- | --- | --- | --- | --- | --- | --- | --- |
| **Outcomes** | **No of participants (studies)** | **Relative effects** | | | **Absolute effect estimates** | | **Certainty/Quality of evidence** | **Plain languages summary** |
|  |  |  |  |  | **Baseline risk for control group (per 1000)** | **Difference (95% CI) (per 1000)** |  |  |
| Hospitalization | 234  (1 cohort study) | Pre-ED epinephrine vs in-ED epinephrine:  OR 0.25 (95% CI 0.10 to 0.62) | | | 170 | -122. (-150 to -87) | Moderate Ꚛ Ꚛ Ꚛ O  (Large magnitude of an effect)^a^ | Early administration probably reduce hospitalization. |
| Median length of ED stay | 234  (1 cohort study) | Pre-ED epinephrine vs in-ED epinephrine= 3 hours vs 4 hours, P=0.003 | | | 4 hours | Not available^b^ | Low Ꚛ Ꚛ O O | Early administration probably reduce the length of ED stay. |
|  | **No of participants (studies)** | | **Time from symptom to**  **administration of epinephrine** | | | **P value** |  |  |
|  |  |  | **Uniphasic grop** | **Biphasic group** | |  |  |  |
| Biphasic anaphylaxis | 47  (1 case control study) | | 1.67 (0-27.07)^c^ hrs | 2.67 (0-15.75)^c^ hr | | 0.115 | Very low Ꚛ O O O  (Serious inconsistence)^f^ | We are very uncertain of the effect of early administration on biphasic anaphylaxis. |
|  | 60  (1 case control study) | | 8.5 ± 13.8^d^ min | 8.2 ± 12.8^d^ min | | 0.94 |  |  |
|  | 107  (1 case control study) | | 40 (1-300)^c^ min | 28 (3-130)^c^ min | | 0.7 |  |  |
|  | 208  (1 case control study) | | 70 (40-135)^e^ min | 240 (122.5-380)^e^ min | | 0.002 |  |  |
|  | 139  (1 case control study) | | 6 (3-13)^e^ min | 4.5 (3-15)^e^ | | Not reported |  |  |
|  | 484  (1 case control study) | | 59 (25-105)^e^ min | 64 (25-175)^e^ min | | P=0.35 |  |  |

ED: emergency department.

^a^ Observational studies started at low certainty/quality of evidence. In addition, we rated up one level for large magnitude of the effect (OR=0.25 and crude RR=0.38 [(95% CI 0.25 to 0.60]), while we did not find factors that can seriously reduce the quality of evidence.

^b^ Length of ED stay was presented as median and interquartile range, so difference could not be calculated.

^c^ Time was presented as median (rang).

^d^ Time was presented as mean (SD).

^e^ Time was presented as median (IQR).

^f^ Results of different studies could not be pooled together. Observational studies started at low certainty/quality of evidence. In addition, we rated down for serious inconsistence (different studies showed different directions).

## Table 5. GRADE summary of findings: Administration methods (IM vs SC) for epinephrine in anaphylaxis

| **Outcomes** | **No of participants (studies)** | **Results** | | **Relative/absolute effects** | **Certainty/Quality of evidence** | **Plain languages summary** |
| --- | --- | --- | --- | --- | --- | --- |
|  |  | **IM group** | **SC group** |  |  |  |
| T_max_ (min) | 17  (1 RCT) | 8 ± 2 | 34 ± 14 | -26 (95% CI -35 to -17) | Moderate Ꚛ Ꚛ Ꚛ O  (Serious indirectness)^a^ | IM probably accelerate the efficacy of epinephrine in managing anaphylaxis^b^. |
| AUC (ng/ml/min) | 17  (1 RCT) | 108 ± 18 | 67 ± 13 | 41 (95% CI 26 to 56) | Low Ꚛ Ꚛ O O  (Very serious indirectness)^c^ | IM may make epinephrine be more effective in the rescue of anaphylaxis^b^. |
| Serious adverse effects | 17  (1 RCT) | 0/8 | 0/9 | Not applicable | Low Ꚛ Ꚛ O O  (Very serious imprecision)^d^ | IM may has no effect on epinephrine-induced serious adverse effects |
| Mild transient adverse effects | 13  (1 crossover RCT) | 4/13 | 3/13 | RR 1.33 (95% CI 0.37 to 4.82) | Low Ꚛ Ꚛ O O  (Very serious imprecision)^d^ | IM may increase the risk of epinephrine-induced mild transient adverse reaction. |
| Overdose | 54 children  (1 cohort study) | 5/7 | 14/19 | RR 0.97 (95% CI 0.56 to to 1.66) | Very low Ꚛ O O O  (Serious risk of bias and imprecision)^e^ | We are very uncertain of the effect of IM on overdose. |
|  | 524 adults  (1 cohort study) | 51/81 | 116/177 | RR 0.96 (95% CI 0.79 to 1.17) |  |  |
| C_max_^f^ | 17  (1 RCT) | 2136 ± 351 | 1802 ± 214 | 334 (95% CI 53 to 615) | Very low Ꚛ O O O  (Very serious indirectness and inconsistence)^g^ | We are very uncertain of the effect of IM on enhancing the efficacy of epinephrine^b^. |
|  | 13  (1 crossover RCT) | 1821 ± 426 | 2877 ± 567 | -1051 (95% CI -1432 to -670) |  |  |

T_max_: time at which maximum plasma epinephrine concentration was achieved; AUC, area under the plasma concentration versus time curve; Cmax, peak plasma epinephrine concentrations;

^a^ RCT started at high quality of evidence. We rated down one level for serious indirectness (indirect outcome).

^b^ This summary doesn’t come from this body of evidence directly and we had rated down the quality evidence because of (very) serious indirectness.

^c^ RCT started at high quality of evidence. We rated down two levels for very serious indirectness (seriously indirect outcome).

^d^ RCT started at high quality of evidence. We rated down three levels: one for serious risk of bias (unclearly defined adverse effect) and two for imprecision (wide confidence interval and insufficient sample size).

^e^ Observational studies started at low certainty/quality of evidence. Then we rated it down for very serious risk of bias (unadjusted analysis) and imprecision (very wide confidence interval).

^f^ One RCT included adults and the another one included children, so they can not be pooled.

^g^ RCT started at high quality of evidence. We rated down three levels for very serious indirectness (seriously indirect outcome) and inconsistence (two studies reported different directions of severe anaphylaxis).

## Table 6. GRADE summary of findings: Administration methods (IM vs IV) for epinephrine in anaphylaxis

| **Outcomes** | **No of participants (studies)** | **Results** | | **Relative effects** | **Certainty/Quality of evidence** | **Plain languages summary** |
| --- | --- | --- | --- | --- | --- | --- |
|  |  | **IM group** | **IV bolus group** |  |  |  |
| Cardiovascular complications^a^ | 243  (1 cohort study) | 2/217 | 3/7 | RR 0.02 (95% CI 0.00 to 0.11) | Low Ꚛ Ꚛ O O  (Serious risk of bias, large magnitude on an effect)^b^ | IM might reduce the risk of adverse cardiovascular complications^a^. |

^a^ Cardiovascular complications: (1) new onset of ventricular fibrillation or tachycardia, atrial flutter or fibrillation, or multifocal atrial tachycardia; (2) acute stroke, defined as a new neurologic deficit18; (3) elevated cardiac troponin T (above 99th percentile of the upper reference limit (normal sensitivity troponin, Roche Elecsys, Hoffman Laroche, Laval, QC; 99th percentile reference limit > 0.01 ng/ml)); and, (4) the following new ischemic ECG findings: ST-segment elevation greater than 1 mm, ST-segment depression greater than 0.5 mm; left bundle branch block; T-wave inversions, or pathological Qwave changes.

^b^ Observational studies started at low certainty/quality of evidence. Additionally, we rated down one level for serious risk of bias (unadjusted analysis) and then rated up one level for very large magnitude on the effect (RR=0.02). Though serious limitation is present, the very large magnitude on the effect still worths rating up one level, since this limitation is unlikely to explain all of the apparent benefit.

## Table 7. GRADE summary of findings: Administration methods (IM vs IV bolus) for epinephrine in anaphylaxis

| **Outcomes** | **No of participants (studies)** | **Results** | | **Relative effects** | **Certainty/Quality of evidence** | **Plain languages summary** |
| --- | --- | --- | --- | --- | --- | --- |
|  |  | **IM group** | **IV bolus group** |  |  |  |
| Adverse cardiovascular event^a^ | 346  (1 cohort study) | 4/316 | 4/30 | RR 0.09 (95% CI 0.03 to 0.36) | Low Ꚛ Ꚛ O O  (Serious risk of bias, large magnitude on an effect)^b^ | IM might reduce the risk of adverse cardiovascular event^a^. |
| Overdose^a^ | 346  (1 cohort study) | 0/316 | 4/30 | RR 0.01 (95% CI 0.00 to 0.20) | Very low Ꚛ O O O  (Serious risk of bias)^c^ | We are very uncertain of th effect of IM on the risk of overdose. |
|  | 54 children  (1 cohort study) | 5/7 | 19/19 | RR 0.70 (95% CI 0.44 to 1.31) |  |  |
|  | 524 adults  (1 cohort study) | 51/81 | 212/213 | RR 0.63 (95% CI 0.535 to 0.748) |  |  |

^a^ Adverse cardiovascular event comprised arrhythmia, ischemia, stroke, angina, hypertension.

^b^ Observational studies started at low certainty/quality of evidence. Additionally, we rated down one level for serious risk of bias (unadjusted analysis) and then rated up one level for very large magnitude on the effect (RR<0.1). Though serious limitation is present, the very large magnitude on the effect still worths rating up one level, since this limitation is unlikely to explain all of the apparent benefit.

^c^ Observational studies started at low certainty/quality of evidence. Additionally, we rated down one level for serious risk of bias (unadjusted analysis).

## Table 8. GRADE summary of findings: Administration methods (IM vs IV infusion) for epinephrine in anaphylaxis

| **Outcomes** | **No of participants (studies)** | **Results** | | **Relative effects** | **Certainty/Quality of evidence** | **Plain languages summary** |
| --- | --- | --- | --- | --- | --- | --- |
|  |  | **IM group** | **IV infusion group** |  |  |  |
| Adverse cardiovascular event^a^ | 320  (1 cohort study) | 4/316 | 0/4 | Not available, no events in IV infusion group | Very low Ꚛ O O O  (Serious risk of bias, very serious imprecision)^b^ | We are very uncertain of the effect of IM in adverse cardiovascular event. |

^a^ Adverse cardiovascular event comprised arrhythmia, ischemia, stroke, angina, hypertension.

^b^ Observational studies started at low certainty/quality of evidence. In addition, we rated down for serious risk of bias (unadjusted analysis) and very serious imprecision (wide confidence interval and insufficient sample size).

## Table 9. GRADE summary of findings: IM site for epinephrine in anaphylaxis

| **Outcomes** | **No of participants (studies)** | **Results** | | **Relative/absolute effects** | **Certainty/Quality of evidence** | **Plain languages summary** |
| --- | --- | --- | --- | --- | --- | --- |
|  |  | **Thigh group** | **Arm group** |  |  |  |
| C_max_ | 13  (1 crossover RCT) | 9722 ± 4801 | 1821 ± 426 | 7901 (95% CI 5281 to 10521) | Low Ꚛ Ꚛ O O  (Very serious indirectness)^a^ | IM on thigh may enhance the efficacy of epinephrine^b^. |
| Mild transient adverse effects | 13  (1 crossover RCT) | 6/13 | 4/13 | RR 1.5 (95% CI 0.55 to 4.10) | Very low Ꚛ O O O  (Serious risk of bias, very serious imprecision)^c^ | We are very uncertain of the effect of injecting in thigh on epinephrine-induced mild transient adverse reaction. |

Cmax, peak plasma epinephrine concentrations

^a^ RCT started at high quality of evidence. We rated down two level for very serious indirectness (indirect outcome).

^b^ This summary doesn’t come from this body of evidence directly and we had rated down the quality evidence because of very serious indirectness.

^c^ RCT started at high quality of evidence. We rated down three levels: one for serious risk of bias (unclearly defined adverse effect) and two for imprecision (very wide confidence interval).

| Table 10. GRADE summary of findings: H1 antagonist in anaphylaxis | | | | | | | | | | | |
| --- | --- | --- | --- | --- | --- | --- | --- | --- | --- | --- | --- |
| **Outcomes** | | **No of participants (studies)** | | **Relative effects** | | **Absolute effect estimates** | | | | **Certainty/Quality of evidence** | **Plain languages summary** |
|  |  |  |  |  |  | **Baseline risk for control group (per 1000)** | | **Difference (95% CI) (per 1000)** | |  |  |
| Admission to ICU/hospital ward | 3498  (1 case control study) | | Prehospital antihistamine vs non prehospital antihistamine:  OR 0.86 (95% CI 0.54 to 1.36) | | Not available^a^ | | Not available^a^ | | Very low Ꚛ O O O  (Very serious imprecision)^b^ | | We are very uncertain of the effect of antihistamine on hospital/ICU admission. |
| Hospitalization | 123  (1 cohort study) | | RR 3.05 (95% CI 1.49 to 6.25) | | 129 | | 393 (95% CI 63.21 to 677.25) | | Very low Ꚛ O O O  (Very serious risk of bias)^c^ | | We are very uncertain of the effect of H1a on hospitalization |
| ≥2 doses of epinephrine | | 965  (1 case control study) | | Pre-ED antihistamine vs non pre-ED antihistamine:  OR 0.50 (95% CI 0.20 to 1.18) | | Not available^a^ | | Not available^a^ | | Very low Ꚛ O O O  (Very serious risk of bias and imprecision)^d^ | We are very uncertain of the effect of pre-ED antihistamine on the risk of ≥2 doses of epinephrine^g^ |
| ED intravenous fluid | | 3498  (1 case control study) | | Prehospital antihistamine vs non prehospital antihistamine  OR 0.82 (95% CI 0.64 to 1.33) | | Not available^a^ | | Not available^a^ | | Very low Ꚛ O O O  (Very serious imprecision)^b^ | We are very uncertain of the effect of prehospital antihistamine on ED intravenous fluid. |
| ≥2 doses of epinephrine | | 965  (1 case control study) | | In-ED antihistamine vs non in-ED antihistamine:  OR 3.6 (95% CI 1.39 to 9.21) | | Not available^a^ | | Not available^a^ | | Very low Ꚛ O O O  (Very serious risk of bias)^e^ | We are very uncertain of the effect of in-ED antihistamine on the risk of ≥2 doses of epinephrine^g^ |
| Biphasic anaphylaxis | | 2067  (9 case control studies) | | Antihistamine vs non-antihistamine:  OR 0.70 (95% CI 0.38 to 1.40) | | Not available^a^ | | Not available^a^ | | Very low Ꚛ O O O  (Very risk of bias and imprecision)^f^ | We are very uncertain of the effect of antihistamine on biphasic anaphylaxis. |
| Subsequent in-ED hypotension | | 340  (1 case control study) | | OR 0.70 (95% CI 0.20 to 2.42) | | Not available^a^ | | Not available^a^ | | Very low Ꚛ O O O  (Very serious risk of bias and imprecision)^g^ | We are very uncertain of the effect of H1a on subsequent in-ED hypotension |

ED: emergency department; H1a: H_1_ antagonist.

^a^ Baseline was not reported, and no study reported this outcome among anaphylaxis patients who didn’t receive H_1_ antagonist, so baseline risk and difference were not available here.

^b^ Observational studies started at low certainty/quality of evidence. Then we rated it down for very serious imprecision (very wide confidence interval).

^c^ Observational studies started at low certainty/quality of evidence. Then we rated it down for very serious risk of bias (unadjusted analysis)

^d^ Observational studies started at low certainty/quality of evidence. Then we rated it down for very serious risk of bias (did not adjust severity of anaphylaxis) and very serious imprecision (very wide confidence interval).

^e^ Observational studies started at low certainty/quality of evidence. Then we rated it down for very serious risk of bias (did not adjust severity of anaphylaxis)

^f^ Observational studies started at low certainty/quality of evidence. Then we rated it down for very serious risk of bias (unadjusted analysis) and very serious imprecision (very wide confidence interval).

^g^ Observational studies started at low certainty/quality of evidence. Then we rated it down for very serious risk of bias (unadjusted analysis) and very serious imprecision (very wide confidence interval).

| Table 11. GRADE summary of findings: Glucocorticoids in anaphylaxis | | | | | | |
| --- | --- | --- | --- | --- | --- | --- |
| **Outcomes** | **No of participants (studies)** | **Relative effects** | **Absolute effect estimates** | | **Certainty/Quality of evidence** | **Plain languages summary** |
|  |  |  | **Baseline risk for control group (per 1000)** | **Difference (95% CI) (per 1000)** |  |  |
| Admission to ICU/hospital ward | 3498  (1 case control study) | Prehospital GCs vs non prehospital GCs:  aOR 2.88 (95% CI 1.13-7.36) | Not available^a^ | Not available^a^ | Low Ꚛ Ꚛ O O | Glucocorticoids may increase the risk of admission to ICU/hospital ward. |
| ICU admission^b^ | 5203  (1 cohort study)^c^ | RR 0.77 (95% CI 0.56 to 1.07) | 45 | -10 (-20 to 3) | Very low Ꚛ O O O  (Very serious risk of bias and imprecision)^c^ | We are very uncertain of the effect of glucocorticoids on ICU admission among hospitalized patients. |
| Prolonged length of stay^d^ | 5203  (1 cohort study) | aOR 0.61 (95% CI 0.41 to 0.93) | Not available^a^ | Not available^a^ | Low Ꚛ Ꚛ O O | Glucocorticoids may reduce the risk of prolonged length of stay^d^. |
| Length of hospital stay | 6161  (1 cohort study) | Not applicable^e^ | 1.84 ± 1.04 days | 0.39 (95% CI 0.29 to 0.49) day | Very low Ꚛ O O O  (Very serious risk of bias)^f^ | We are very uncertain of the effect of glucocorticoids on length of hospital stay. |
| 3-day allergy-related revisit^h^ | 5203  (1 cohort study)^i^ | aOR 1.01 (95% CI 0.50 to 2.05) | Not available^a^ | Not available^a^ | Very low Ꚛ O O O  (Very serious imprecision)^g^ | We are very uncertain of the effect of glucocorticoids on 3-day allergy-related revisit among discharged patients. |
| 7-day allergy-related revisit | 473  (1 cohort study) | aOR 1.12 (95% CI 0.41 to 3.27) | 56 | 13 (-33 to 127) | Very low Ꚛ O O O  (Very serious imprecision)^g^ | We are very uncertain of the effect of glucocorticoids on 7-day allergy-related revisit. |
| 10-day readmission | 5113 (1 cohort study) | aRR 1.13 (95% CI 0.60 to 2.16) | 1 | 0 (0 to 1) | Very low Ꚛ O O O  (Very serious imprecision)^g^ | We are very uncertain of the effect of glucocorticoids on 10-day readmission. |
| ≥2 doses of ED epinephrine | 3498  (1 case control study) | Prehospital GCs vs non prehospital GCs:  aOR 0.79 (95% CI 0.19 to 3.39) | Not available^a^ | Not available^a^ | Very low Ꚛ O O O  (Very serious imprecision)^g^ | We are very uncertain of the effect of glucocorticoids on multiple ED epinephrine. |
| ED IV fluids | 3498  (1 case control study) | Prehospital GCs vs non prehospital GCs:  aOR 1.53 (95% CI 0.77 to 3.03) | Not available^a^ | Not available^a^ | Very low Ꚛ O O O  (Very serious imprecision)^g^ | We are very uncertain of the effect of glucocorticoids on ED IV fluids. |
| Biphasic anaphylaxis | 3547  (12 case control studies) | OR 0.84 (95% CI 0.43 to 1.63) | Not available^a^ | Not available^a^ | Very low Ꚛ O O O  (Very serious risk of bias and imprecision)^d^ | We are very uncertain of the effect of glucocorticoids on biphasic anaphylaxis. |
| Subsequent in-ED hypotension | 340  (1 case control study) | OR 0.70 (95% CI 0.20 to 2.42) | Not available^a^ | Not available^a^ | Very low Ꚛ O O O  (Very serious risk of bias and imprecision)^c^ | We are very uncertain of the effect of epinephrine on subsequent in-ED hypotension |
| Epinephrine administration beyond the day of anaphylaxis-onset | 5203  (1 cohort study) | aOR 0.63 (95% CI 0.48 to 0.84) | Not available^a^ | Not available^a^ | Low Ꚛ Ꚛ O O | Glucocorticoids may reduce the risk of epinephrine administration beyond the day of anaphylaxis-onset among hospitalized patients. |
| Total hospitalization costs | 6161  (1 cohort study) | Not applicable^d^ | 6334 ±1013 ¥^j^ | 3896 (95% CI 2464 to 5562) ¥^i^ | Very low Ꚛ O O O  (Very serious risk of bias)^f^ | We are very uncertain of the effect of glucocorticoids on total hospitalization cost. |

More details about included studies for this clinical question could be seen in the systematic review^[3]^.

ED: emergency department.

^a^ Baseline was not reported, and no study reported this outcome among anaphylaxis patients who didn’t receive glucocorticoids, so baseline risk and difference were not available here.

^b^ ICU admission was only reported for hospitalized anaphylaxis patients in this study.

^c^ Observational studies started at low certainty/quality of evidence. Then we rated it down for very serious risk of bias (unadjusted analysis) and very serious imprecision (very wide confidence interval).

^d^ Prolonged length of stay was defined as hospital stay of ≥2 days. This outcome was only reported for hospitalized anaphylaxis patients in this study.

^e^ This continuous outcome was presented as absolute difference.

^f^ Observational studies started at low certainty/quality of evidence. Then we rated it down for very serious risk of bias (did not adjust severity of anaphylaxis and co-interventions).

^g^ Observational studies started at low certainty/quality of evidence. Then we rated it down for very serious imprecision (very wide confidence interval).

^h^ 3-day allergy-related revisit was only reported for discharged patients.

^i^ Japanese yuan

## Table 12. GRADE summary of findings: Monitor duration for anaphylaxis

| **Outcomes** | **No of participants (studies)** | **Relative effects** | **Absolute effect estimates** | | **Certainty/Quality of evidence** | **Plain languages summary** |
| --- | --- | --- | --- | --- | --- | --- |
|  |  |  | **Baseline risk for control group (per 1000)** | **Difference (95% CI) (per 1000)** |  |  |
| Hospitalization^a^ | 439  (1 cohort study) | 4 hrs group vs 8 hrs group:  RR 0.44 (95% CI 0.34 to 0.56) | 582 | -326 (95% CI  -326 to -256) | Very low Ꚛ O O O  (Serious risk of bias)^b^ | We are very uncertain of the effect of reducing monitor duration from 8hrs to four hrs on hospitalization. |
| 3-day allergy-related revisit^c^ | 267  (1 cohort study) | 4 hrs group vs 8 hrs group:  RR 4.18 (95% CI 0.50 to 34.98) | 13 | 41.34 (95% CI  -7 to 441) | Very low Ꚛ O O O  (Serious risk of bias, very serious imprecision)^d^ | We are very uncertain of the effect of reducing monitor duration from 8hrs to four hrs on 3-day allergy related revisit among discharged patients. |

^a^ In 4hrs group, if any of below was present, patient was recommended to be hospitalized: ① history of biphasic or severe reactions, ② progression of or persistent symptoms, ③ history of severe asthma, ④ current asthma flare, ⑤ hypotension during ED stay, ⑥ requires >1 epinephrine dose, or ⑦ requires fluid bolus. But this handling was not reported to be practiced in 8 hrs group.

^b^ Observational studies started at low certainty/quality of evidence. In addition, we rated down for serious risk of bias (as the preceding note, two groups were not comparable).

^c^ 3-day allergy-related revisit was only reported for discharged patients.

^d^ Observational studies started at low certainty/quality of evidence. In addition, we rated down for serious risk of bias (unadjusted analysis) and very serious imprecision (very wide confidence interval).

## Table 13. GRADE summary of findings: H1 antagonist for preventing all-cause anaphylaxis

| **Outcomes** | **No of participants (studies)** | **Relative effects** | **Absolute effect estimates** | | **Certainty/Quality of evidence** | **Plain languages summary** |
| --- | --- | --- | --- | --- | --- | --- |
|  |  |  | **Baseline risk for control group (per 1000)** | **Difference (95% CI) (per 1000)** |  |  |
| Anaphylaxis**^a^** | 2376  (1 cohort study) | aOR 0.34 (95% CI 0.17 to 0.70) | 34 | -22 (95% CI  -28 to -10) | For patients with allergic reaction:  Low Ꚛ Ꚛ O O  For unselected population:  Very low Ꚛ O O O  (Very serious indirectness)^b^ | H1a may reduce the risk of anaphylaxis in patients with allergic reaction.  We are very uncertain of the effect of H1a on preventing anaphylaxis in unselected population |
| Severe anaphylaxis**^a^**^c^ | 2376  (1 cohort study) | RR 0.70 (95% CI 0.18 to 2.64) | 6 | -2 (95% CI -5 to 10) | For patients with allergic reaction and unselected population:  Very low Ꚛ O O O  (Very serious risk of bias and imprecision)^d^ | We are very uncertain of the effect of H1a on preventing anaphylaxis in patients with allergic reaction and unselected population. |

H1a: H_1_ antagonist.

^a^ The time interval between H1a administration and onset of anaphylaxis was not reported. However, it should be shorter than the time to anaphylaxis (48 min [IQR 27 to 167] in H1a group and 29 min [IQR 21 to 60] in non-H1a group)

^b^ Observational studies started at low certainty/quality of evidence. We rated down for very serious risk indirectness (patients with allergic reaction).

^c^ Criteria for severe case of anaphylaxis in included study: Patients experienced hypotension (sBP<90), oxygen saturation <92% and/or neurological symptoms during transportation by EMS and/or observation at ED.

^d^ Observational studies started at low certainty/quality of evidence. We rated down for very serious risk of bias (unadjusted analysis) imprecision (very wide confidence interval). Since the evidence equality for patients with allergic reaction is very low, considering indirectness, the evidence quality for unselected population is also very low.

## Table 14. GRADE summary of findings: Epinephrinea for preventing antivenom-induced anaphylaxis

| **Outcomes** | **No of participants (studies)** | **Relative effects** | **Absolute effect estimates** | | **Certainty/Quality of evidence** | **Plain languages summary** |
| --- | --- | --- | --- | --- | --- | --- |
|  |  |  | **Baseline risk for control group (per 1000)** | **Difference (95% CI) (per 1000)** |  |  |
| Anaphylaxis | 1007  (1 RCT) | OR 0.85 (95% CI 0.71 to 1.00)^b^ | 762^b^ | -144 (95% CI  -22 to 0)^b^ | For antivenom recipients:  Low Ꚛ Ꚛ O O  (Serious indirectness and imprecision)^c^  For unselected population:  Very low Ꚛ O O O  (Very serious indirectness, serious imprecision)^d^ | Epinephrine may reduce the risk of antivenom-induced anaphylaxis.  We are very uncertain of the effect of epinephrine on preventing anaphylaxis among unselected population. |
| Severe anaphylaxis^e^ | 1007  (1 RCT) | OR 0.62 (95% CI 0.51 to 0.74)^b^ | 333^b^ | -127 (95% CI  -163 to -87)^b^ | For antivenom recipients:  Moderate Ꚛ Ꚛ Ꚛ O  (Serious indirectness)^f^  For unselected population:  Very low Ꚛ O O O  (Very serious indirectness)^g^ | Epinephrine probably reduce the risk of antivenom-induced sever anaphylaxis.^e^  We are very uncertain of the effect of epinephrine on preventing severe anaphylaxis among unselected population.^e^ |

^a^ SC Epinephrine was administrated immediately before infusion of antivenom in included study.

^b^ Both intervention and control group contained people receiving hydrocortisone or promethazine but the proportions in two group were comparable.

^c^ We rated down two levels: one for serious indirectness (unverified anaphylaxis criteria) and serious imprecision (wide confidence interval).

^d^ We rated down three levels for very serious indirectness (unverified anaphylaxis criteria, antivenom-induced anaphylaxis) and serious imprecision (wide confidence interval).

^e^ Criteria for severe case of anaphylaxis in included study: drowsiness or altered consciousness, systolic BP < 80 mm Hg, cyanosis, confusion.

^f^ We rated down one level for serious indirectness (unverified anaphylaxis criteria)

^g^ We rated down three levels for very serious indirectness (unverified anaphylaxis criteria, antivenom-induced anaphylaxis)

## Table 15. GRADE summary of findings: Glucocorticoidsa for preventing antivenom-induced anaphylaxis

| **Outcomes** | **No of participants (studies)** | **Relative effects** | **Absolute effect estimates** | | **Certainty/Quality of evidence** | **Plain languages summary** |
| --- | --- | --- | --- | --- | --- | --- |
|  |  |  | **Baseline risk for control group (per 1000)** | **Difference (95% CI) (per 1000)** |  |  |
| Anaphylaxis | 1007  (1 RCT) | OR 1.07 (95% CI 0.87 to 1.32)^b^ | 740^b^ | 52 (95% CI  -96 to 236)^b^ | For antivenom recipients and unselected population:  Very low Ꚛ O O O  (Serious indirectness, very serious imprecision)^c^ | We are very uncertain of the effect of glucocorticoids on preventing anaphylaxis among antivenom recipients and unselected population. |
| Severe anaphylaxis^d^ | 1007  (1 RCT) | OR 0.80 (95% CI 0.53 to 1.21)^b^ | 314^b^ | -63 (95% CI  -148 to 66)^b^ | For antivenom recipients and unselected population:  Very low Ꚛ O O O  (Serious indirectness, very serious imprecision)^c^ | We are very uncertain of the effect of glucocorticoids on preventing severe anaphylaxis among antivenom recipients and unselected population. |

^a^ IV hydrocortisone was administrated immediately before infusion of antivenom in included study.

^b^ Both intervention and control group contained people receiving epinephrine or promethazine but the proportions in two group were comparable.

^c^ We rated down three levels: one for serious indirectness (unverified anaphylaxis criteria) and two for very serious imprecision (very wide confidence interval). Since the evidence equality for antivenom recipients is very low, considering indirectness, the evidence quality for unselected population is also very low.

^d^ Criteria for severe case of anaphylaxis in included study: drowsiness or altered consciousness, systolic BP < 80 mm Hg, cyanosis, confusion.

## Table 16. GRADE summary of findings: H1 antagonista for preventing antivenom-induced anaphylaxis

| **Outcomes** | **No of participants (studies)** | **Relative effects** | **Absolute effect estimates** | | **Certainty/Quality of evidence** | **Plain languages summary** |
| --- | --- | --- | --- | --- | --- | --- |
|  |  |  | **Baseline risk for control group (per 1000)** | **Difference (95% CI) (per 1000)** |  |  |
| Anaphylaxis^b^ | 1007  (1 RCT) | OR 1.00 (95% CI 0.74 to 1.35)^c^ | 747^c^ | 0 (95% CI  -269 to 261)^c^ | For antivenom recipients and unselected population:  Very low Ꚛ O O O  (Serious indirectness, very serious imprecision)^d^ | We are very uncertain of the effect of H1a on preventinganaphylaxis among antivenom recipients and unselected population. |
|  | 101  (1 RCT) | RR 0.98 (95% CI 0.50 to 1.93) | 250 | -5 (95% CI -236 to 232) |  |  |
| Severe anaphylaxis | 1007  (1 RCT) | OR 0.87 (95% CI 0.50 to 1.52)^ce^ | 327^c^ | -43 (95% CI  -163 to 170)^c^ | For antivenom recipients and unselected population:  Very low Ꚛ O O O  (Serious indirectness, very serious imprecision and inconsistency)^g^ | We are very uncertain of the effect of H1a on preventing severe anaphylaxis among antivenom recipients and unselected population. |
|  | 101  (1 RCT) | RR 1.06 (95% CI 0.07 to 16.50)^f^ | 19 | 1.14 (95% CI -18 to 295) |  |  |

H1a: H_1_ antagonist.

^a^ IV promethazine was administrated immediately before infusion of antivenom in the RCT recruiting 1007 patients; IV promethazine was administrated 30 min before infusion of antivenom in the RCT recruiting 101 patients;

^b^ Criteria in two included studies were both unverified and not the same, and the control group in the first study contained people receiving epinephrine or promethazine, so they could not be pooled together.

^c^ Both intervention and control group contained people receiving epinephrine or promethazine but the proportions in two group were comparable.

^d^ We rated down three levels: one for serious indirectness (unverified anaphylaxis criteria) and two for very serious imprecision (very wide confidence interval). Since the evidence equality for antivenom recipients is very low, considering indirectness, the evidence quality for unselected population is also very low.

^e^ Criteria for severe case of anaphylaxis in this study: drowsiness or altered consciousness, systolic BP < 80 mm Hg, cyanosis, confusion.

f Criteria for severe case of anaphylaxis in this study: glottal oedema, hypotension, and shock.

^g^ We rated down three levels: one for serious indirectness (unverified anaphylaxis criteria), two for very serious imprecision (very wide confidence interval) and inconsistency (two studies reported different directions of severe anaphylaxis). Since the evidence equality for antivenom recipients is very low, considering indirectness, the evidence quality for unselected population is also very low.

## Table 17. GRADE summary of findings: Glucocorticoidsa plus H1 antagonista for preventing antivenom-induced anaphylaxis

| **Outcomes** | **No of participants (studies)** | **Relative effects** | **Absolute effect estimates** | | **Certainty/Quality of evidence** | **Plain languages summary** |
| --- | --- | --- | --- | --- | --- | --- |
|  |  |  | **Baseline risk for control group (per 1000)** | **Difference (95% CI) (per 1000)** |  |  |
| Anaphylaxis | 129  (1 cohort study) | RR 0.04 (95% CI 0.01 to 0.27) | 487 | -458 (95% CI -482 to -356) | For antivenom recipients and unselected population:  Very low Ꚛ O O O  (Very serious risk of bias, serious indirectness)^b^ | We are very uncertain of the effect of GCs plus H1a on preventinganaphylaxis among antivenom recipients and unselected population. |
| Severe anaphylaxis | 129  (1 cohort study) | RR 0.29 (95% CI 0.03 to 2.39) | 66 | -47 (95% CI  -64 to -91) | For antivenom recipients and unselected population:  Very low Ꚛ O O O  (Very serious risk of bias and imprecision, serious indirectness)^c^ | We are very uncertain of the effect of GCs plus H1a on preventing severe anaphylaxis among antivenom recipients and unselected population. |

GCs: glucocorticoids; H1a: H_1_ antagonist.

^a^ IV promethazine and hydrocortisone was administrated 15-20 min before infusion of antivenom in included study.

^b^ Observational studies started at low certainty/quality of evidence. We rated down for very serious risk of bias (two groups were included in different period and received antivenom from different administration ways, unadjusted analysis) and serious indirectness (unverified anaphylaxis criteria). Since the evidence equality for antivenom recipients is very low, considering indirectness, the evidence quality for unselected population is also very low.

^c^ Observational studies started at low certainty/quality of evidence. We rated down for very serious risk of bias (two groups were included in different period and received antivenom from different administration ways, unadjusted analysis) and imprecision (very wide confidence interval), serious indirectness (unverified anaphylaxis criteria). Since the evidence equality for antivenom recipients is very low, considering indirectness, the evidence quality for unselected population is also very low.

**Reference**

1. Guyatt G, Oxman AD, Akl EA, et al. GRADE guidelines: 1. Introduction-GRADE evidence profiles and summary of findings tables. J Clin Epidemiol. 2011;64(4):383-94
2. Schünemann H, Brożek J, Guyatt G H, et al. Handbook for grading the quality of evidence and the strength of recommendations using the GRADE approach. Updated October 2013. Available: https://gdt.gradepro.org/app/handbook/handbook.html#h.f7lc8w9c3nh8 (accessed 2020 May. 10).
3. Li X, He N, Zheng H et al. Diagnostic criteria of anaphylaxis: systematic review and meta-analysis. Another supplementary material for this guideline (https://www.frontiersin.org/articles/10.3389/fphar.2022.845689/full#supplementary-material).
4. Li X, Cui C, Wu M Zhai et al. Epinephrine for the treatment of anaphylaxis: systematic review and meta-analysis. Front Pharmacol, 2021. Another supplementary material for this guideline (https://www.frontiersin.org/articles/10.3389/fphar.2022.845689/full#supplementary-material)..
5. Li X, Xiong Y, Zheng X et al. Glucocorticoids for the treatment of anaphylaxis: systematic review and meta-analysis. Front Pharmacol, 2021. Another supplementary material for this guideline (https://www.frontiersin.org/articles/10.3389/fphar.2022.845689/full#supplementary-material).
6. Li X, Zheng S, He N et al. Prophylactic premedication for Anaphylaxis: a systematic review. Front Pharmacol, 2021. Another supplementary material for this guideline (https://www.frontiersin.org/articles/10.3389/fphar.2022.845689/full#supplementary-material).
7. Quality and characteristics of included systematic reviews

## Table 1. Quality of included systematic reviews by using AMSTAR 2

| Study | 1 | 2^a^ | 3 | 4^a^ | 5 | 6 | 7^a^ | 8 | 9^a^ | 10 | 11^a^ | 12 | 13^a^ | 14 | 15^a^ | 16 |
| --- | --- | --- | --- | --- | --- | --- | --- | --- | --- | --- | --- | --- | --- | --- | --- | --- |
| Sheikh 2008/2010 | 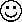 | 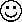 | 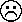 | 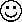 | 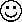 | 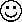 | 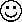 | NA | NA | NA | NA | NA | NA | NA | NA | 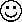 |
| Sheikh 2012 | 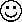 | 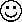 | 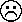 | 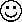 | 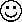 | 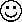 | 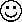 | NA | NA | NA | NA | NA | NA | NA | NA | 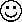 |
| Rubin 2014 | 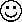 | 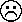 | 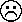 | 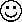 | 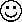 | 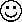 | 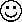 | NA | NA | NA | NA | NA | NA | NA | NA | 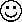 |
| Dhami 2013 | 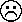 | 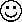 | 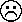 | 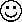 | 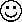 | 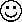 | 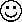 | 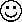 | 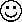 | 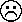 | NA | NA | 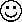 | 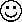 | NA | 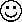 |
| Safdar 2001 | 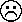 | 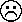 | 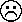 | 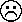 | 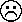 | 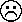 | 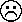 | 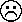 | 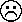 | 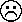 | NA | NA | 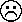 | 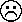 | NA | 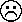 |
| Sheikh 2007/2010 | 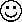 | 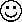 | 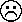 | 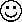 | 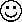 | 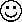 | 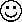 | NA | NA | NA | NA | NA | NA | NA | NA | 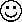 |
| Choo 2010  /2012 | 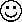 | 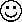 | 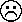 | 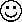 | 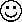 | 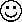 | ? | NA | NA | NA | NA | NA | NA | NA | NA | 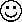 |
| Habib 2011 | 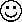 | 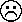 | 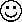 | 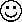 | ? | ? | 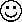 | 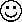 | 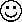 | 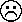 | 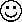 | 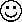 | 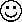 | 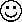 | 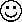 | 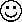 |
| Delaney 2006 | 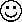 | 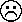 | 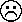 | 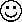 | 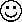 | 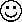 | 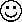 | 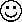 | 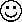 | 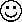 | 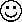 | 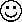 | 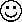 | 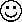 | 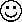 | 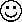 |
| Tramer 2006 | 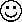 | 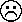 | 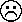 | ? | ? | 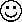 |  |  |  |  |  |  |  |  |  |  |
| Nurmatov 2008 |  |  |  |  |  |  |  |  |  |  | NA | NA |  |  | NA |  |
| Liyanage 2017 |  |  |  |  | ? | ? |  |  |  |  | NA | NA | ? |  | NA |  |
| Choo 2007 |  |  |  |  |  |  |  | NA | NA | NA | NA | NA | NA | NA | NA |  |

1. Did the research questions and inclusion criteria for the review include the components of PICO?
2. Did the report of the review contain an explicit statement that the review methods were established prior to the conduct of the review and did the report justify any significant deviations from the protocol?
3. Did the review authors explain their selection of the study designs for inclusion in the review?
4. Did the review authors use a comprehensive literature search strategy?
5. Did the review authors perform study selection in duplicate?
6. Did the review authors perform data extraction in duplicate?
7. Did the review authors provide a list of excluded studies and justify the exclusions?
8. Did the review authors describe the included studies in adequate detail?
9. Did the review authors use a satisfactory technique for assessing the risk of bias (RoB) in individual studies that were included in the review?
10. Did the review authors report on the sources of funding for the studies included in the review?
11. If meta-analysis was performed did the review authors use appropriate methods for statistical combination of results?
12. If meta-analysis was performed, did the review authors assess the potential impact of RoB in individual studies on the results of the meta-analysis or other evidence synthesis?
13. Did the review authors account for RoB in individual studies when interpreting/discussing the results of the review?
14. Did the review authors provide a satisfactory explanation for, and discussion of, any heterogeneity observed in the results of the review?
15. If they performed quantitative synthesis did the review authors carry out an adequate investigation of publication bias (small study bias) and discuss its likely impact on the results of the review?
16. Did the review authors report any potential sources of conflict of interest, including any funding they received for conducting the review?

^a^ AMSTAR 2 critical domains.

## Table 2. Characteristic of included systematic reviews

| Study | Population | Intervention/Exposure | Control | Outcome | Study type | Date for literature search | Included studies |
| --- | --- | --- | --- | --- | --- | --- | --- |
| Sheikh 2008/2010 | Anaphylaxis patients | Epinephrine | No intervention, placebo, other adrenergic agonists; and different  treatment approaches to the administration of epinephrine | (1) Death; (2) resolution of upper airway obstruction; (3)resolution of lower airway obstruction (symptomatic bronchospasm); (4)improvement in arterial blood pressure (greater than 50 mm Hg systolic); (5) resolution of urticarial; (6) requirement for second dose of adrenaline; (7) admission to hospital; (8) length of emergency department stay; (9) length of hospital stay; (10) re-presentation for therapy within 24 hours; (11) adverse events due to therapy, in either treatment arm | RCT or Quasi-RCT | November 2020 | No |
| Sheikh 2012 | Anaphylaxis patients | Epinephrine auto-injector | No treatment, placebo, alternative pharmacological agent, or adrenaline by an alternative  route. | (1) Death; (2) process outcome; (3) resolution of airway obstruction (4)improvement in arterial blood pressure (> 50 mm Hg systolic in an adult); (5) hospital attendance or admission; (6) e-presentation for treatment within 72 hours; (7) cost-effectiveness; (8) adverse events due to treatment in either treatment arm | RCT or Quasi-RCT | January 2012 | No |
| Safdar 2001 | Older patients | Subcutaneous epinephrine | NR | Cardiovascular adverse effect | NR | September 1999 | /^a^ |
| Sheikh 2007/2010 | Anaphylaxis patients | H1a | Placebo or no intervention | (1) clinical improvement by any objective measure; (2) mortality rate; (3) hospitalization rate; (4) length of emergency department visit; (5) length of hospital stay; (6) re-presentation rate to hospital; (7) iatrogenic adverse events; (8) rate of persistent/delayed/biphasic reactions. | RCT or Quasi-RCT | November 2010 | No |
| Choo 2010  /2012 | Anaphylaxis patients | Glucocorticoids | Any control treatment (either placebo, adrenaline (epinephrine), antihistamine, or any combination of  these). | (1) Mortality rate; (2) Prevention of biphasic or prolonged anaphylaxis; (3) Incidence of cardiovascular manifestations (4) Incidence of respiratory manifestations; (5) Incidence of gastrointestinal manifestations; (6) Incidence of other clinical manifestations as included in the studies | RCT or Quasi-RCT | September 2011 | No |
| Rubin 2014 | Pediatric anaphylaxis patients | Acute treatment (pharmacologic, supportive measures) | Any control treatment | Any and all outcome measures | RCT | December 2012, | No |
| Dhami 2013 | This SR aimed at assessing the effectiveness of interventions for: (1) the acute management of anaphylaxis, (2) non-pharmacological approaches for the long-term management of anaphylaxis. | | | | SR +/- meta analyses, RCT, Quasi-RCTs, controlled clinical trials, controlled before-after designs, interrupted time series studies | NR | 2 SRs which found no RCT or quasi-RCT |
| Choo 2010  /2012 | Anaphylaxis patients | Glucocorticoids | Any control treatment (either placebo, adrenaline (epinephrine), antihistamine, or any combination of  these). | (1) Mortality rate; (2) Prevention of biphasic or prolonged anaphylaxis; (3) Incidence of cardiovascular manifestations (4) Incidence of respiratory manifestations; (5) Incidence of gastrointestinal manifestations; (6) Incidence of other clinical manifestations as included in the studies | RCT or Quasi-RCT | September 2011 | No |
| Habib 2011 | Snakebite victims who receive antivenoms | Premedication | Placebo or no pre-medication | Early adverse reaction | RCT or cohort study | September 2010 | 3 RCTs  4 cohort studies |
| Delaney 2006 | Patients receiving x-ray contrast as an injection | Premedication | Placebo or no pre-medication | Allergic reactions | Prospective RCT | June 2005 | 6 RCTs |
| Tramer 2006 | Patients receiving iodinated contrast media | Premedication | Placebo or no premedication | Distinct allergy related symptoms, symptom categories (grades), non-specific  Symptoms, adverse drug reactions | RCT | October 2005 | 9 RCTs |
| Nurmatov 2008 | Patients who had previously experienced anaphylaxis | Any educational  intervention | NR | Any description of the  components of these plans, barriers and facilitators to their use, and any  insights into their likely clinical effectiveness or acceptability | NR | 2007 | 19 studies of various design |
| Liyanage 2017 | Anaphylaxis patients | Glucocorticoids | NR | NR | NR | March 2016 | 22 human studies |
| Choo 2007 | Patients who had previously experienced anaphylaxis | Any educational intervention | NR | (1) Clinical improvement by any objective measures; (2) Hospitalization rate: A&E attendance, admission and readmission; (3) Length of hospital stay; (4) Mortality rate; (5) Morbidity (symptoms, use of rescue medications, quality of life, functional health status, days off work/school); (6) Health service use (length of emergency department visit, primary care practitioners visits) | RCT or Quasi-RCT | June 2006 | No |

NR: not reported.

^a^ This study cited many articles in the result and it was difficult to summarize what studies had been included.

**Reference**

1. Shea BJ, Reeves BC, Wells G, et al. AMSTAR 2: a critical appraisal tool for systematic reviews that include randomised or non-randomised studies of healthcare interventions, or both. BMJ. 2017;358:j4008. Published 2017 Sep 21. DOI:10.1136/bmj.j4008
2. Quality and recommendations of other guideline

## Table 1. Quality of included guidelines by using AGREE II^[1]^

| Number | Guidelines | Domain scores（%） | | | | | | Overall quality |
| --- | --- | --- | --- | --- | --- | --- | --- | --- |
|  |  | 1.Scope and purpose | 2. Stakeholder involvement | 3. Rigor of development | 4. Clarity and presentation | 5. Applicability | 6. Editorial independence |  |
| 1 | EAACI | 97 | 79 | 77 | 100 | 75 | 100 | 6 |
| 2 | ANZAAG & ANZCA | 94 | 63 | 68 | 100 | 73 | 100 | 5 |
| 3 | AAAAI | 100 | 73 | 61 | 100 | 65 | 100 | 5 |
| 4 | WAO | 94 | 66 | 44 | 100 | 65 | 100 | 4 |
| 5 | UKRC | 32 | 100 | 67 | 100 | 67 | 100 | 4 |
| 6 | DGAKI | 69 | 69 | 39 | 97 | 71 | 83 | 4 |
| 7 | AAGBI | 83 | 55 | 22 | 78 | 54 | 50 | 2 |
| 8 | SSAI | 92 | 52 | 24 | 82 | 27 | 50 | 3 |

EAACI: European Academy of Allergy and Clinical Immunology; ANZAAG: Australian and New Zealand Anaesthetic Allergy Group; ANZCA: Australian and New Zealand College of Anaesthetists; AAAAI: American Academy of Allergy, Asthma and Immunology; WAO: World Allergy Organization; RCUK: The Resuscitation Council; DGAKI: the German Society for Allergology and Clinical Immunology; AeDA: he Association of German Allergologists; GPA: the Society of Pediatric Allergy and Environmental Medicine; DAAU: the German Academy of Allergology and Environmental Medicine; BVKJ: the German Professional Association of Pediatricians; ÖGAI: the Austrian Society for Allergology and Immunology; SGAI: the Swiss Society for Allergy and Immunology; DGAI: the German Society of Anaesthesiology and Intensive Care Medicine; DGP: the German Society of Pharmacology; DGPM: the German Society for Psycho somatic Medicine; AGATE: he German Working Group of Anaphylaxis Training and Education; DAAB: the patient organization German Allergy and Asthma Association; ACAAI: the American College of Allergy, Asthma and Immunology; ASCIA: Australasian Society of Clinical Immunology and Allergy Anaphylaxis Working Party; AAGBI: Association of Anaesthetists of Great Britain and Ireland; SSAI: Scandinavian Society of Anaesthesiology and Intensive Care Medicine.

## Table 2. Summary of recommendations in other guidelines

| Guidelines | 1. EAACI | | 2. NZAAG & ANZCA | | 3. AAAAI | 4. WAO | 5. UKRC | 6. DGAKI | 7. AAGBI | 8. SSAI |
| --- | --- | --- | --- | --- | --- | --- | --- | --- | --- | --- |
| Grading used in guidelines | recommendation grade: A-D | | recommendation grade: A-D | | recommendation grade (2014): Strong recommendation, moderate, weak, no recommendation  recommendation grade (2015): Strong recommendation, recommendation, weak, no recommendation |  |  |  |  |  |
| 1. Diagnosis | Note 1.1 | | Note 1.2 | | Note 1.3 | Note 1.4 | Note 1.5 | Note 1.6 | Note 1.7 | Note 1.8 |
| 2. Grading |  | | Note 2.1 | |  |  |  | Note 2.2 |  |  |
| 3. Non-professional preparation | Note 3.1 | |  | |  |  |  | Note 3.2 |  |  |
| 4. Emergency monitor | Note 4.1 | | Note 4.2 | | Note 4.3 | Note 4.4 | Note 4.5 | Note 4.6 |  |  |
| 5. Artificial airways |  | | Note 5.1 | | Note 5.2 | Note 5.3 |  | Note 5.4 | Note 5.5 |  |
| 6.1 Status of epinephrine | Note 6.1.1 | | Note 6.1.2 | | Note 6.1.3 | Note 6.1.4 | Note 6.1.5 | Note 6.1.6 | Note 6.1.7 | Note 6.1.8 |
| 6.2 Timing of epinephrine |  |  |  | | Note 6.2.1 | Note 6.2.2 | Note 6.2.3 | Note 6.2.4 |  | Note 6.2.5 |
| 6.3 Status of I.M. epinephrine | Note 6.3.1 | | Note 6.3.2 | |  |  | Note 6.3.3 | Note 6.3.4 | Note 6.3.5 | Note 6.3.6 |
| 6.4 Dosage of I.M. epinephrine | Note 6.4.1 | | Note 6.4.2 | | Note 6.4.3 | Note 6.4.4 | Note 6.4.5 | Note 6.4.6 | Note 6.4.7 | Note 6.4.8 |
| 6.5 Site of I.M. epinephrine | Note 6.5.1 | | Note 6.5.2 | | Note 6.5.3 | Note 6.5.4 | Note 6.5.5 | Note 6.5.5 |  |  |
| 6.6 Status of I.V. bolus epinephrine |  | | Note 6.6.1 | | Note 6.6.2 | Note 6.6.3 | Note 6.6.4 | Note 6.6.5 | Note 6.6.6 |  |
| 6.7 Dosage of I.V. bolus epinephrine |  | | Note 6.6.1 | | Note 6.6.2 | Note 6.6.3 | Note 6.6.4 | Note 6.6.5 | Note 6.6.6 | Note 6.6.7 |
| 6.8 Status of I.V. infusion epinephrine | Note 6.8.1 | | Note 6.8.2 | | Note 6.8.3 | Note 6.8.4 | Note 6.8.5 | Note 6.8.6 | Note 6.8.7 | Note 6.8.8 |
| 6.9 Dosage of I.V. infusion epinephrine |  | | Note 6.9.1 | | Note 6.9.2 | Note 6.9.3 | Note 6.9.4 |  | Note 6.9.5 | Note 6.9.6 |
| 6.10 Status of S.C. epinephrine | Note 6.10.1 | |  | | Note 6.10.2 | Note 6.10.3 | Note 6.10.4 | Note 6.10.5 |  |  |
| 6.11 Contraindication of epinephrine | Note 6.11.1 | |  | | Note 6.11.2 | Note 6.11.3 | Note 6.11.4 | Note 6.11.5 |  |  |
| 6.12 Reduce ADR^a^ of epinephrine | Note 6.12.1 | | Note 6.12.2 | | Note 6.12.3 | Note 6.12.4 | Note 6.12.5 | Note 6.12.6 | Note 6.12.7 |  |
| 7. H1a | Note 7.1 | | Note 7.2 | | Note 7.3 | Note 7.4 | Note 7.5 | Note 7.6 | Note 7.7 | Note 7.8 |
| 8. β2-agonist | Note 8.1 | | Note 8.2 | | Note 8.3 | Note 8.4 | Note 8.5 | Note 8.6 | Note 8.7 | Note 8.8 |
| 9. Glucocorticoids | Note 9.1 | | Note 9.2 | | Note 9.3 | Note 9.4 | Note 9.5 | Note 9.6 | Note 9.7 | Note 9.8 |
| 10. Fluid resuscitation | Note 10.1 | | Note 10.2 | | Note 10.3 | Note 10.4 | Note 10.5 | Note 10.6 | Note 10.7 | Note 10.8 |
| 11. Monitor duration | Note 11.1 | | Note 11.2 | | Note 11.3 | Note 11.4 |  | Note 11.5 |  |  |
| 12. Report drug-induced anaphylaxis |  | |  | |  |  | Note 12.1 |  | Note 12.2 |  |
| 13. Prophylactic intervention | Note 13.1 |  | | Note 13.2 | | Note 13.3 |  |  | Note 13.4 | Note 13.5 |
| 14. Patient education | Note 15.1 | | Note 15.2 | | Note 15.3 | Note 15.4 | Note 15.5 | Note 15.6 |  | Note 15.7 |

Guidelines are sorted from high quality to low quality (quality of guidelines is showed on Table 1)

EAACI: European Academy of Allergy and Clinical Immunology; ANZAAG: Australian and New Zealand Anaesthetic Allergy Group; ANZCA: Australian and New Zealand College of Anaesthetists; AAAAI: American Academy of Allergy, Asthma and Immunology; WAO: World Allergy Organization; RCUK: The Resuscitation Council; DGAKI: the German Society for Allergology and Clinical Immunology; AeDA: he Association of German Allergologists; GPA: the Society of Pediatric Allergy and Environmental Medicine; DAAU: the German Academy of Allergology and Environmental Medicine; BVKJ: the German Professional Association of Pediatricians; ÖGAI: the Austrian Society for Allergology and Immunology; SGAI: the Swiss Society for Allergy and Immunology; DGAI: the German Society of Anaesthesiology and Intensive Care Medicine; DGP: the German Society of Pharmacology; DGPM: the German Society for Psycho somatic Medicine; AGATE: he German Working Group of Anaphylaxis Training and Education; DAAB: the patient organization German Allergy and Asthma Association; ACAAI: the American College of Allergy, Asthma and Immunology; ASCIA: Australasian Society of Clinical Immunology and Allergy Anaphylaxis Working Party; AAGBI: Association of Anaesthetists of Great Britain and Ireland; SSAI: Scandinavian Society of Anaesthesiology and Intensive Care Medicine.

1. C grade recommendation: CNIAID/FAAN criteria
2. Text: Anaphylaxis should be considered if skin signs co-exist with bronchospasm or hypotension. Hypotension or tachycardia alone, especially where this is unresponsive to vasopressors or is unanticipated should raise suspicion. Bradycardia may also be a presenting sign of anaphylaxis. Bronchospasm or difficulty with ventilation may be a feature of anaphylaxis or the sole presenting feature in some cases. The absence of skin signs does not rule out the diagnosis, as skin signs may not appear until the circulation is restored.
3. Strong Recommendation (2014): Base the diagnosis of anaphylaxis on the history and physical examination, using scenarios described by the National Institutes of Allergy and Infectious Disease (NIAID) Panel but recognizing that there is a broad spectrum of anaphylaxis presentations that require clinical judgment. Do not rely on signs of shock for the diagnosis of anaphylaxis.

Text (2015): the criteria used by the NIAID/FAAN workshop to diagnose anaphylaxis have been shown to be useful in an emergency department setting to accurately establish a diagnosis of anaphylaxis.

1. Text: Mainly adopted CNIAID/FAAN criteria. Cox L 2010 supported footnotes for anaphylaxis with involvement of only one body organ system, and Simons 2007 supported footnotes for anaphylaxis in infants and young children.
2. Text: A single set of criteria will not identify all anaphylactic reactions. There is a range of signs and symptoms, none of which are entirely specific for an anaphylactic reaction; however, certain combinations of signs make the diagnosis of an anaphylactic reaction more likely.

Table for recognition of an anaphylactic reaction (2008): Anaphylaxis is likely when all of the following 3 criteria are met: • Sudden onset and rapid progression of symptoms; • Life-threatening Airway and/or Breathing and/or Circulation problems • Skin and/or mucosal changes (flushing, urticaria, angioedema)

The following supports the diagnosis: Exposure to a known allergen for the patient. Remember: • Skin or mucosal changes alone are not a sign of an anaphylactic reaction. • Skin or mucosal changes can be subtle or absent in up to 20% of reactions (some patients can have only a decrease in blood pressure, i.e., a Circulation problem). • There can also be gastrointestinal symptoms (e.g., vomiting, abdominal pain, incontinence).

1. Text: In a consensus conference, the following symptoms were regarded as being of specific importance for the diagnosis of anaphylaxis (then cited CNIAID/FAAN criteria)
2. Text: Clinical features include hypotension, tachycardia or bradycardia; cutaneous flushing, rash or urticaria; bronchospasm; hypoxia; angioedema and cardiac arrest. If an adverse event such as hypotension or bronchospasm occurs during anaesthesia it is appropriate to suspect anaphylaxis unless there exists a significantly more likely cause.
3. Table 2. Classification of clinical manifestations of anaphylaxis during anaesthesia.

Clinical manifestations of Class I: Generalised cutaneous signs: erythema, urticaria with or without angioedema.

Clinical manifestations of Class II: Moderate multiorgan involvement with cutaneous signs, hypotension and tachycardia, bronchial hyperreactivity (cough, ventilatory impairment)

Clinical manifestations of Class III: Severe life-threatening multiorgan involvement that requires specific treatment: collapse, tachycardia or bradycardia, cardiac arrhythmias, bronchospasm; the cutaneous signs may be absent or occur only after the arterial blood pressure recovers.

Clinical manifestations of Class IV: Circulatory or respiratory arrest

Clinical manifestations of Class V: Death due to a lack of response to cardiorespiratory resuscitation

1. Text: A critical step once the provisional diagnosis of anaphylaxis has been made is the grading of the severity of anaphylaxis in order to administer the recommended initial dose of adrenaline (epinephrine).

Figure 5:

Mild (Grade1): Generalised mucocutaneous signs: Erythema, Urticaria +/- Angioedema

Moderate (Grade 2): Moderate—Multi-organ manifestation may include: Hypotension, tachycardia; Evidence of bronchospasm, cough, difficult ventilation; Mucocutaneous signs.

Life threatening (Grade 3): Life threatening and requiring immediate and specific treatment: Severe hypotension; Bradycardia or tachycardia, arrhythmias; Severe bronchospasm, and/or airway oedema; Cutaneous signs may be absent, or present only after correction of hypotension.

Arrest (Grade 4): Cardiopulmonary Arrest

1. Table 2. Severity grading of anaphylactic reactions

Grade I. skin: Itch, Flush, Urticaria, Angioedema

Grade II. skin: Itch, Flush, Urticaria, Angioedema. Abdomen: Nausea, Cramps. Airways: Rhinorrhea, Hoarseness, Dyspnea. Cardiovascular system: Tachycardia (> 20/min), Hypertension (> 20 mm Hg syst.), Arrhythmia.

Grade III. Skin: Itch, Flush, Urticaria, Angioedema. Abdomen: Vomiting, Defecation. Airways. Laryngeal edema, Bronchospasm, Cyanosis. Cardiovascular system: Shock.

Grade IV: Skin: Itch, Flush, Urticaria, Angioedema: Vomiting, Defecation. Airways: Respiratory arrest. Cardiovascular system: Cardiac arrest.

1. Text from Example of an individualized anaphylaxis emergency action plan:

1. Immediately administer adrenaline auto-injector into the upper outer thigh

2. Call an ambulance stating that the patient is having an anaphylactic reaction

3. Lay person having the reaction down (with legs up if possible); if there is difficulty in breathing, allow them to sit up but not stand

4. If no improvement after 5 min, administer a second adrenaline auto-injector.

When in doubt, administer the adrenaline auto-injector.

1. Text: symptom-orientated self-medication. correct positioning. making an emergency call. Potential suspected elicitors (foods, insects, drugs) should be preserved if possible.
2. Text: Patients who are given intravenous adrenaline should be monitored with continuous ECG, pulse oximetry, and frequent noninvasive blood pressures.
3. Text: Standard monitoring as recommended by ANZCA PS18 Guidelines on Monitoring during Anaesthesia (http://www.anzca.edu.au/documents/ps18-2013-recommendations-on-monitoring-during-ana) should be utilised throughout resuscitative efforts.
4. Text (2015): Monitor and record the patient’s blood pressure, cardiac rate and function, respiratory status, and oxygenation at frequent and regular intervals. Start frequent oxygen saturation measurement, start continuous noninvasive monitoring, and obtain an electrocardiogram, if available.
5. Text and Table 5. Basic Management of Anaphylaxis: At frequent and regular intervals, monitor the patient’s blood pressure, cardiac rate and function, respiratory status and oxygenation and obtain electrocardiograms; start continuous noninvasive monitoring if possible.
6. Text: All patients who have had an anaphylactic reaction should be monitored (e.g., by ambulance crew, in the emergency department, etc.) as soon as possible. Minimal monitoring includes pulse oximetry, non-invasive blood pressure and 3- lead ECG.
7. Text for Anaphylaxis with predominant cardiovascular reaction: Continuous monitoring of blood pressure and pulse are necessary.

Text: In anaphylaxis with life-threatening systemic reactions monitoring in an intensive care unit is recommended.

1. Text (2015): If airway oedema is suspected then early endotracheal intubation should be considered.
2. Anaphylaxis treatment protocol (2015): Refractory anaphylaxis: Advanced airway management. Use supraglottic airway, endotracheal intubation, or cricothyroidotomy for marked stridor, severe laryngeal edema, or when ventilation using the bag-valve-mask is inadequate and EMS has not arrived.
3. Texr for management of respiratory distress: Supplemental oxygen should be administered by face mask or by oropharyngeal airway at a flow rate of 6–8 L/min to all patients with respiratory distress and those receiving repeated doses of epinephrine (Table 5). It should also be considered for any patient with anaphylaxis who has concomitant asthma, other chronic respiratory disease, or cardiovascular disease.
4. Text: A laryngeal mask or a laryngeal tube can be helpful. Only in rare cases will endotracheal intubation by an experienced physician (usually emergency physician, anaesthesiologist) become necessary.

Text: Anaphylaxis with predominant obstruction of the upper airways. In the case of insufficient response to these therapeutic measures ( intramuscular injection of adrenaline and administration of inhaled adrenaline ), coniotomy may be required.

1. Text for Immediate management: Intubate the trachea if necessary and ventilate the lungs with oxygen.
2. C grade recommendation: Adrenaline is potentially lifesaving and must therefore promptly be administered as the first-line treatment for the emergency management of anaphylaxis.
3. C grade recommendation for immediate management: Adrenaline (epinephrine) is pivotal in management and, in the recommended doses, causes vasoconstriction, bronchodilation, increased cardiac output, reduced mucosal oedema, and reduced mediator release
4. Strong recommendation (2015): At the onset of anaphylaxis, administer epinephrine intramuscularly in the mid-outer thigh;
5. Text: The World Health Organization (www.who.int) classifies epinephrine (adrenaline) as an essential medication for the treatment of anaphylaxis. Previous WAO publications and anaphylaxis guidelines published in indexed, peer-reviewed journals consistently emphasize prompt injection of epinephrine as the first-line medication of choice in anaphylaxis. The evidence base for prompt epinephrine injection in the initial treatment of anaphylaxis is stronger than the evidence base for the use of antihistamines and glucocorticoids in anaphylaxis.
6. Text: Adrenaline is the most important drug for the treatment of an anaphylactic reaction. Adrenaline should be given to all patients with lifethreatening features.
7. Text: The most important drug in the acute therapy of anaphylaxis is adrenaline (epinephrine).
8. Text for immediate management: Administer adrenaline intravenously.
9. Text: The cornerstones of treatment are adrenaline and fluid therapy. Adrenaline is a highly potent and efficient treatment in most cases of anaphylaxis.
10. Text (2015): Because the clinical course of anaphylaxis can be unpredictable, prompt and early use of epinephrine should be considered even with mild symptoms or single-system involvement
11. Text: Epinephrine should be injected by the intramuscular route in the mid-anterolateral thigh as soon as anaphylaxis is diagnosed or strongly suspected
12. Text: Adrenaline seems to work best when given early after the onset of the reaction.
13. Text: In a patient not in need of resuscitation, immediate intramuscular application of a dose of 0.3 to 0.5 mg adrenaline (body weight range 30 to 50 kg) to the outer upper thigh is the drug therapy of first-choice.
14. Text: It should be administered as early as possible
15. B grade recommendation: Adrenaline should be administered by intramuscular injection into the mid-outer thigh.
16. C grade recommendation for Immediate management: IM adrenaline into the lateral thigh should be considered in the initial management of perioperative anaphylaxis where IV access is not yet established or is lost, where haemodynamic monitoring is not in situ at the start of the reaction, or while awaiting preparation of an adrenaline infusion.
17. Text: The intramuscular route is the best for most individuals who have to give adrenaline to treat an anaphylactic reaction.
18. Text: In a patient not in need of resuscitation, immediate intramuscular application of a dose of 0.3 to 0.5 mg adrenaline (body weight range 30 to 50 kg) to the outer upper thigh is the drug therapy of first-choice.

Only ‘Table 7. Pharmacotherapy for children, adolescents and adults under out-patient conditions’ indicated dose of Adrenaline Intravenous bolus, while ‘Table 6. Pharmacotherapy for children, adolescents and adults in intensive care’ didn't

1. Text for Drug doses in children: The intramuscular route is preferred where there is no venous access or where establishing venous access would cause a delay in drug administration.
2. Table 3. SSAI Guideline on treatment of anaphylactic reactions during anaesthesia.

Adults: Without i.v. access: 0.5–0.8 mg i.m.

Children: Without i.v. access: 0.005–0.01 mg/kg i.m.

1. Text: Intramuscular adrenaline (1 mg/ml) should be given at a dose of 0.01 ml/kg of body weight to a maximum total dose of 0.5 ml. When using adrenaline auto-injectors, patients weighing between 7.5–25 kg should receive 0.15 mg dose with patients being moved to 0.3 mg dose at 25–30 kg. There are no data to inform us which patients should receive a 0.5-mg dose auto-injector, if this is available.

D grade recommendation: The adrenaline dose can be repeated after at least a 5-min interval.

1. Figure for Immediate management:

I.M. Adrenaline (Adult), 1:1000 1mg/mL, 500 mcg lateral thigh, every 5 minutes prn. I.M. Adrenaline (Peadiatric), 1:1000 1mg/mL, lateral thigh, <6 years =0.15 mL (150mcg), 6-12 years = 0.3mL (300mcg), every 5 minutes prn.

1. Anaphylaxis treatment protocol (2015): Immediate measures: Epinephrine intramuscular. 0.3-0.5 mg (0.01 mg/kg for children); repeat intramuscular epinephrine every 5-15 min for up to 3 injections if the patient is not responding.

Text (2015): The adult dose of 1:1,000 epinephrine is 0.2 to 0.5 mL, whereas the pediatric dose is 0.01 mg/kg, with a maximum of 0.3 mg.

1. Text: in a dose of 0.01 mg/kg of a 1:1,000 (1 mg/mL) solution, to a maximum of 0.5 mg in adults (0.3 mg in children). Depending on the severity of the episode and the response to the initial injection, the dose can be repeated every 5–15 minutes, as needed. Most patients respond to 1 or 2 doses of epinephrine injected intramuscularly promptly; however, more than 2 doses are occasionally required.
2. Table for Intramuscular adrenaline:

Adrenaline IM dose—–adults, 0.5 mg IM (=500 g = 0.5 mL of 1:1000) adrenaline.

Adrenaline IM dose—–children, >12 years: 500 g IM (0.5 mL), i.e., same as adult dose 300 g (0.3 mL) if child is small or prepubertal; >6—12 years: 300 g IM (0.3 mL); >6 months—6 years: 150 g IM (0.15 mL); <6 months: 150 g IM (0.15 mL).

Text: Repeat the IM adrenaline dose if there is no improvement in the patient’s condition. Further doses can be given at about 5-min intervals according to the patient’s response

1. Text: In a patient not in need of resuscitation, immediate intramuscular application of a dose of 0.3 to 0.5 mg adrenaline (body weight range 30 to 50 kg) to the outer upper thigh is the drug therapy of first-choice. In case of no response, the injection can be repeated every 5–10 minutes, depending on side effects.

Table 7. Pharmacotherapy for children, adolescents and adults under out-patient conditions: Adrenaline Intramuscular 0.01 ml/kg bw (1 mg/1 ml)

1. Text for Drug doses in children:

> 12 years: 500 lg IM (0.5 ml of a 1:1000 solution), 300 lg IM (0.3 ml of a 1:1000 solution) if the child is small.

6–12 years: 300 lg IM (0.3 ml of a 1:1000 solution)

Up to 6 years: 150 lg IM (0.15 ml of a 1:1000solution)

1. Table 3:

Adults: Without i.v. access: 0.5–0.8 mg i.m. Children: Without i.v. access: 0.005–0.01 mg/kg i.m.

1. B grade recommendation: Adrenaline should be administered by intramuscular injection into the mid-outer thigh
2. Figure for Immediate management:

I.M. Adrenaline, lateral thigh.

1. Strong recommendation (2015): At the onset of anaphylaxis, administer epinephrine intramuscularly in the mid-outer thigh;
2. Text: Epinephrine should be injected by the intramuscular route in the mid-anterolateral thigh as soon as anaphylaxis is diagnosed or strongly suspected.
3. Text: The best site for IM injection is the anterolateral aspect of the middle third of the thigh.
4. Text: the outer upper thigh.
5. Text： The Immediate Management Card also has the words ‘Moderate’ and ‘Life Threatening’ as descriptors of ‘Grade 2 and 3’ respectively in the ‘Initial IV Adrenaline Bolus’ section
6. Text (2015): For imminent or established cardiopulmonary arrest, rapidly establish venous access and administer an intravenous bolus dose of epinephrine because ventricular arrhythmias have been reported after epinephrine administration.
7. Text: If cardiac arrest is imminent or has already occurred, an intravenous bolus dose of epinephrine is indicated; however, in other anaphylaxis scenarios, this route of administration should be avoided, for the reasons listed below.
8. Text: Patients who require repeated IM doses of adrenaline may benefit from IV adrenaline. It is essential that these patients receive expert help early. If the patient requires repeated IV bolus doses of adrenaline, start an adrenaline infusion.

Table for Intravenous (IV) adrenaline (for specialist use only): Ensure patient is monitored.

1. Text: If the patient is unstable or during resuscitation, i.e. in case of respiratory and/or circulatory arrest,

Both Table 6. Pharmacotherapy for children, adolescents and adults in intensive care and Table 7. Pharmacotherapy for children, adolescents and adults under out-patient conditions indicated dose of Adrenaline Intravenous bolus.

1. Text for immediate management: Administer adrenaline intravenously (following dosage indicated this meat IV bolus). (This is a guideline for anaphylaxis associated with anaesthesia).
2. Figure: Initial I.V. Adrenaline Bolus (Adult), Dilution 1 mg in 10 mL = 100 mcg/mL. Give dose below every 1-2 minutes prn. Increase dose if unresponsive. Moderate (Grade 2) 20 mcg/0.2 mL, Life Threatening (Grade 3) 100-200 mcg = 1-2 mL.

Initial I.V. Adrenaline Bolus (Peadiatric), Dilution 1 mg in 50 mL = 200 mcg/L. Give dose below every 1-2 minutes prn. Increase dose if unresponsive. Moderate (Grade 2) 0.1 mL/kg (2mcg/kg), Life Threatening (Grade 3) 0.2-0.5 mL/kg (4-10mcg/kg).

1. Text (2015): For adults, the dose is 1 mg intravenously (as a 1:10,000 dilution). For a child, the dose is 0.01 mL/kg to a maximum single dose of 1 mg (give as a 1:10,000 dilution). This can be repeated every 3 to 5 minutes as cardiopulmonary resuscitation is continued.
2. Text: dilute solutions appropriate for intravenous administration (1:10,000 [0.1 mg/mL] or 1:100,000 [0.01 mg/mL]).
3. Table for IV adrenaline (for specialist use only):

Adrenaline IV bolus dose—–adult. Titrate IV adrenaline using 50 μg boluses according to response. If repeated adrenaline doses are needed, start an IV adrenaline infusion. The pre-filled 10 mL syringe of 1:10,000 adrenaline contains 100 μg/mL. A dose of 50 μg is 0.5 mL, which is the smallest dose that can be given accurately. Do not give the undiluted 1:1000 adrenaline concentration IV.

Adrenaline IV bolus dose—–children. There is no evidence on which to base a dose recommendation—–the dose is titrated according to response. A child may respond to a dose as small as 1 g/kg. This requires very careful dilution and checking to prevent dose errors.

1. Text: a dilution of 1 mg adrenaline in 10 ml NaCl 0.9 %, i.e. a solution of 0.1 mg/ml is administered, depending on effects and side effects, under continuous control of circulatory parameters.

Table 6. Pharmacotherapy for children, adolescents and adults in intensive care and Table 7. Pharmacotherapy for children, adolescents and adults under out-patient conditions: Adrenaline Intravenous bolus1 0.1 ml/kg bw (of 1 mg/10 ml) for <30 kg bw, 0,05–0,1 ml/kg bw (of 1 mg/10 ml) for > 30 kg bw

1. Text: An initial dose of 50 μg (0.5 ml of 1 : 10 000 solution) is appropriate (adult dose). Several doses may be required if there is severe hypotension or bronchospasm.
2. Table 3. SSAI Guideline on treatment of anaphylactic reactions during anaesthesia:

Adults: Use diluted adrenaline i.v. maximum concentration 0.1 mg/ml

Mild to moderate reaction: 0.01–0.05 mg i.v.

Circulatory collapse: 0.1–1.0 mg i.v.

Children:

Mild to moderate reaction: 0.001–0.005 mg/kg i.v.

Circulatory collapse: 0.01 mg/kg i.v.

1. D grade recommendation: Patients who require repeated intramuscular doses of adrenaline may benefit from an adrenaline infusion.
2. D grade recommendation for Immediate management: After three boluses of adrenaline via either the IV or IM route an adrenaline infusion should be prepared and commenced as early as possible in the clinically appropriate dosage.
3. Anaphylaxis treatment protocol (2015): Additional measures: Epinephrine infusion. For patients with inadequate response to intramuscular epinephrine and intravenous saline, give epinephrine by continuous infusion by micro-drip in office setting (infusion pump in hospital setting).
4. Text: if shock is imminent or has already developed, epinephrine needs to be given by slow intravenous infusion. Ideally, epinephrine should be administered intravenously only by physicians who are trained, experienced and equipped to give vasopressors through infusion pump and titrate
5. the dose frequently, based on continuous monitoring of blood pressure and cardiac rate and function.
6. Text: Patients who require repeated IM doses of adrenaline may benefit from IV adrenaline. It is essential that these patients receive expert help early. If the patient requires repeated IV bolus doses of adrenaline, start an adrenaline infusion.
7. A continuous infusion of approx. 0.05–1 µg/kg/minute is equally effective (as 1 mg adrenaline bolus for patient who is unstable or during resuscitation, i.e. in case of respiratory and/or circulatory arrest).

Table 6. Pharmacotherapy for children, adolescents and adults in intensive care: Adrenaline Continuous infusion 0.05–1.0 µg/kg/min

1. Text: If several doses of adrenaline are required, consider starting an intravenous infusion of adrenaline (adrenaline has a short half-life).
2. Text: Continuous infusion of adrenaline is advantageous in patients who need repetitive doses of adrenaline.
3. Figure for Immediate management: Adrenaline Infusion (Adult), 3 mg Adrenaline in 50 mL saline. Conmence at 3 mL/hr = 3 mcg/min. Titrate to max. 40 mL/hr = 40 mcg/min. (Infusion rate 0.05-0.5 mcg/kg/min)

Adrenaline Infusion (Peadiatric), 1 mg Adrenaline in 50 mL saline (20 mcg/mL). Conmence at 0.3 mL/kg/hr = 0.1 mcg/kg/min. Titrate to max. 6 mL/kg/hr = 2 mcg/kg/min.

1. Anaphylaxis treatment protocol (2015): Additional measures: Epinephrine infusion. add 1 mg (1 mL of 1:1,000) of epinephrine to 1,000 mL of 0.9 NL saline; start infusion at 2 μg/min (2 mL/min = 120 mL/h) and increase up to 10 μg/min (10 mL/min = 600 mL/h); titrate dose continuously according to blood pressure, cardiac rate and function, and oxygenation.
2. Text: dilute solutions appropriate for intravenous administration (1:10,000 [0.1 mg/mL] or 1:100,000 [0.01 mg/mL]).
3. Table for IV adrenaline (for specialist use only): An infusion of adrenaline with the rate titrated according to response in the presence of continued haemodynamic monitoring is an effective way of giving adrenaline during anaphylaxis. Use local guidelines for the preparation and

infusion of adrenaline.

1. Text for Drug doses in children: Prepare a syringe containing 1 ml of 1:10 000 adrenaline for each 10 kg body weight (0.1 ml/kg of 1:10 000 adrenaline solution = 10 μg/kg). Titrate to response, starting with a dose of one-tenth of the contents of the syringe, i.e. 1 μg/kg. Often a child will respond to as little as 1 μg/kg. In smaller children, further dilution may be needed to allow dose titration
2. Table 3. SSAI Guideline on treatment of anaphylactic reactions during anaesthesia:

Adults: i.v. infusion starting at: 0.05–0.1 μg/kg/min

1. Text: The use of subcutaneous or inhaled adrenaline in the treatment of anaphylaxis is not recommended.
2. Text (2015): Published studies on epinephrine pharmacokinetics in patients not in anaphylaxis have shown that intramuscular administration in the vastus lateralis muscle produces a more rapid rate of increase in blood epinephrine levels than subcutaneous or intramuscular administration in the deltoid muscle.
3. Text: Subcutaneous epinephrine injection causes local vasoconstriction that potentially leads to delayed absorption.
4. Text: The subcutaneous or inhaled routes for adrenaline are not recommended for the treatment of an anaphylactic reaction because they are less effective than the IM route.
5. Text: Subcutaneous injection of adrenaline is no longer recommended because of insufficient absorption resulting in delayed onset of action.
6. Text: There are no absolute contraindications to treatment with adrenaline in a patient experiencing anaphylaxis; benefits outweigh the risks in the elderly and patients with pre-existing cardiovascular disease.
7. Text (2015): However, there is no absolute contraindication to the administration of epinephrine as clearly stated in the Food and Drug Administration package insert for AIE. This includes patients with acute coronary syndrome, and although the risk-to-benefit ratio needs to be assessed with care in such patients, it usually favors the administration of epinephrine. Moreover, cardiovascular disease does not “forbid” the use of epinephrine in the treatment of anaphylaxis. Nonetheless, there are no means by which data can be collected to support this statement because clearly the problem does not lend itself to experimental analysis.
8. Text: Although caution is necessary and dosing errors need to be avoided, epinephrine is not contraindicated in the treatment of anaphylaxis in patients with known or suspected cardiovascular disease, or in middle-aged or elderly patients without any history of coronary artery disease who are at increased risk of ACS only because of their age. There is no absolute contraindication to treatment with epinephrine in such patients, although the benefits and risks need to be carefully weighed.
9. Text (1999): Some fluorohydrocarbons used as refrigerants as well as cocaine sensitise the heart to epinephrine (adrenaline) and are contraindications to its use.
10. Text: In case of severe life-threatening anaphylaxis there is no absolute contraindication for adrenaline. The indication should, however, be carefully considered in patients with pre-existing heart disease.
11. Text: Adrenaline infusion must be given by those experienced in the use of vasopressors in their daily clinical practice, for example anesthetists, ED, and critical care doctors. Intravenous adrenaline in patients with adequate circulation may cause life-threatening hypertension, myocardial ischemia, and arrhythmias. Patients who are given intravenous adrenaline should be monitored with continuous ECG, pulse oximetry, and frequent noninvasive blood pressures.
12. D grade recommendation for refractory management: An arterial line is highly recommended where possible to aid cardiovascular monitoring, blood sampling and continuous monitoring of adrenaline effects.

Text for Immediate management: As adrenaline has a narrow therapeutic window clinicians need to be aware of the potential for toxicity including accidental overdose, particularly during crisis management.

1. Text (2015): If an infusion of epinephrine is started in the office setting, then monitor by available means (eg, every-minute blood pressure and pulse and electrocardiographic monitoring, if available) and be prepared to treat ventricular arrhythmias.
2. Summary from text: correct dose and concentration are important in reducing adverse effect of epinephrine; monitor the cardiac rate and unction in the IV infusion of epinephrine.
3. Text for IM adrenaline: Monitor the patient as soon as possible (pulse, blood pressure, ECG, pulse oximetry). This will help monitor the response to adrenaline.

Text: Patients who are given IV adrenaline must be monitored—–continuous ECG and pulse oximetry and frequent non-invasive blood pressure measurements as a minimum.

1. Text: Control of pulse and blood pressure is mandatory.
2. Text for Drug doses in children: Adrenaline. Intravenous. Check carefully for decimal point and concentration errors.
3. B grade recommendation: Third-line interventions. Oral H1- (and H2)-antihistamines may relieve cutaneous symptoms of anaphylaxis.
4. B grade recommendation for post crisis management: Oral antihistamines are useful for the symptomatic treatment of urticaria, angioedema and pruritus.
5. Anaphylaxis treatment protocol (2015): Optional treatment (efficacy has not been established): H1 antihistamine. Consider giving 25-50 mg of diphenhydramine intravenously for adults and 1 mg/kg (maximum 50 mg) for children; use 10 mg of cetirizine if an oral antihistamine is administered; once there is full recovery, there is no evidence that this medication needs to be continued.
6. Text: In anaphylaxis, H1-antihistamines relieve itching, flushing, urticaria, angioedema, and nasal and eye symptoms; however, they should not be substituted for epinephrine because they are not life-saving; that is, they do not prevent or relieve upper airway obstruction, hypotension, or shock.

Table for second line medicaitons: H1-antihistamine for intravenous infusion eg. chlorpheniramine 10 mg (adult), 2.5-5 mg (child) or diphenhydramine 25-50 mg (adult) (1 mg/kg, maximum 50 mg [child])

1. Text: Antihistamines are a second line treatment for an anaphylactic reaction. The evidence to support their use is weak, but there are logical reasons for them. Antihistamines (H1-antihistamine) may help counter histamine-mediated vasodilation and bronchoconstriction. They may not help in reactions depending in part on other mediators but they have the virtue of safety. Used alone, they are unlikely to be life-saving in a true anaphylactic reaction. Inject chlorphenamine slowly intravenously or intramuscularly. The dose of chlorphenamine depends on age.
2. Text: Compared to adrenaline, antihistamines show a slower onset of action; however, they show a favorable benefit/side effect profile in a broad range of indications. An effect upon the allergic reaction can be assumed and therefore, antihistamines should be given early in all anaphylactic reactions in order to block the effects of histamine. Officially the maximum licensed dose of oral antihistamines is recommended. The only H1 antihistamines registered for intravenous application in the acute treatment of anaphylaxis are the first-generation substances dimetindene (0.1 mg/kg bw) and clemastine (0.05 mg/ kg bw) with their well-known sedating side effects.
3. Text for Secondary management: Administer chlorphenamine 10 mg IV (adult dose).

Text for Drug doses in children:

Chlorphenamine. > 12 years: 10 mg IM or IV slowly

6 to 12 years: 5 mg IM or IV slowly

6 months to 6 years: 2.5 mg IM or IV slowly

< 6 months: 250 μg/kg IM or IV slowly

1. Text: Corticosteroids and antihistamines have a place as secondary treatment for anaphylaxis, and help to prevent oedema, cutaneous symptoms and relapse of the anaphylactic reaction, which can occur up to 24 h after the initial reaction.

Table 3. SSAI Guideline on treatment of anaphylactic reactions during anaesthesia:

Adults: H1 antagonist, e.g. Clemastin 2 mg or Deksklorfeniramin 10 mg or Promethazin 50 mg given i.v.

Children: e.g. Clemastin 0.0125–0.025 mg/kg or Deksklorfeniramin 5 mg or Promethazin 0.3–1.0 mg/kg given i.v./i.m.

1. D grade recommendation: Inhaled (nebulized) short-acting beta-2 agonists should additionally be given to relieve symptoms of bronchoconstriction.
2. Adult refractory management card, recommendation for resistant bronchospasm:

Salbutamol, metered dose inhaler 12 puffs (1200 mcg); I.V. bolus 100-200 mcg +/- infusion 5-25 mcg/min.

1. Recommendation (2015): In addition to epinephrine administered for anaphylaxis, consider administering a nebulized β2-agonist (eg, albuterol) for signs and symptoms of bronchospasm.

Anaphylaxis treatment protocol (2015): Additional measures: Albuterol. Consider administration of 2.5-5 mg of nebulized albuterol in 3 mL of saline for lower airway obstruction; repeat as necessary every 15 min.

1. Text: Extrapolating from their use in acute asthma, selective beta-2 adrenergic agonists such as salbutamol (albuterol) are sometimes given in anaphylaxis as additional treatment for wheezing, coughing, and shortness of breath not relieved by epinephrine. Although this is helpful for lower respiratory tract symptoms, these medications should not be substituted for epinephrine because they have minimal alpha-1 adrenergic agonist vasoconstrictor effects and do not prevent or relieve laryngeal edema and upper airway obstruction, hypotension, or shock.

Table for second line medicaitons: ß2-adrenergic agonist, eg. salbutamol (albuterol) solution, 2.5 mg/3 mL or 5 mg/3 mL (adult), (2.5 mg/3 mL [child]) given by nebulizer and face mask.

1. Text: Bronchodilators. If the patient has asthma-like features alone, follow the British Thoracic Society—–SIGN asthma guidelines (www.britthoracic.org.uk). As well as the drugs listed above, consider further bronchodilator therapy with salbutamol (inhaled or IV), ipratropium (inhaled), aminophyline (IV) or magnesium (IV).
2. Text: In the case of mainly bronchial obstruction, additional administration of an inhalative β-adrenoreceptor agonist, e.g. salbutamol or terbutaline, at a dose of 2–4 puffs, is effective. A spacer device should be used in order to improve the efficacy of inhalation when using an aerosol spray.
3. Text for Secondary management: Treat persistent bronchospasm with an intravenous infusion of salbutamol.
4. Table 3. SSAI Guideline on treatment of anaphylactic reactions during anaesthesia: Nebulised β2-agonist may be used for symptomatic treatment of bronchospasm, but is not first-line treatment.
5. D grade recommendation: Oral or intravenous glucocorticoids are commonly used in anaphylaxis and are thought to possibly prevent protracted anaphylaxis symptoms, particularly in patients with concomitant asthma, and also biphasic reactions; however, this has not been proven and they have a slow onset of action. Oral or parenteral glucocorticoids may be given once first- and second-line therapies have been administered. High-dose nebulized glucocorticoids may be beneficial for upper airway obstruction.
6. D grade recommendation for post crisis management: Steroids have been of benefit in the management of other allergic diseases and they are recommended as part of secondary management. They may be useful in cases where there is a protracted reaction.

Figure for post crisis management: Dexamethasone 0.1-0.4 mg/kg (Pediatric maximum 12 mg), Hydrocortisone 2-4 mg/kg (Pediatric maximum 200 mg).

1. Anaphylaxis treatment protocol (2015): Optional treatment (efficacy has not been established): Corticosteroids. Administer 1-2 mg/kg up to 125 mg per dose, intravenously or orally, of methylprednisolone or an equivalent formulation; once there is full recovery, there is no evidence that this medication needs to be continued.
2. Text: Glucocorticoids switch off transcription of a multitude of activated genes that encode proinflammatory proteins. Extrapolating from their use in acute asthma, the onset of action of systemic glucocorticoids takes several hours. Although they potentially relieve protracted anaphylaxis symptoms and prevent biphasic anaphylaxis, these effects have never been proven.

Table for second line medications: glucocorticoid for intravenous infusion, eg. hydrocortisone 200 mg (adult), maximum 100 mg (child); or methylprednisolone 50-100 mg (adult); 1 mg/kg, maximum 50 mg (child)

1. Text: Steroids (after initial resuscitation). Corticosteroids may help prevent or shorten protracted reactions. In asthma, early corticosteroid treatment is beneficial in adults and children. There is little evidence on which to base the optimum dose of hydrocortisone in anaphylaxis. In hospital patients with asthma, higher doses of hydrocortisone do not seem to be better than smaller doses. Inject hydrocortisone slowly by the intravenously or intramuscularly. The dose of hydrocortisone for adults and children depends on age (Figure 3)

Figure 3. The anaphylaxis algorithm: Adults or child >12 years, chlorphenamine (IM or slow IV) 10 mg, hydrocortisone (IM or slow IV) 200 mg; child 6-12 years: 5 mg and 100 mg respectively; child 6 months-6 years: 2.5 mg and 50 mg respectively; child <6 months: 250 micrograms/kg and 25 mg respectively.

1. Text: Due to their slow onset of action, glucocorticoids play a minor role in the acute phase of anaphylaxis treatment. However, glucocorticoids are effective in the treatment of asthma and against protracted or biphasic anaphylactic reactions. An unspecific membrane stabilizing effect within the first 10–30 minutes of application of high dose glucocorticoids (500–1,000 mg) independent of the potency of the glucocorticoids has been postulated in review articles. When there is no intravenous catheter, glucocorticoids may be applied rectally, especially in small children (e.g. prednisolone suppositories) or orally.
2. Text for Secondary management: Administer hydrocortisone 200 mg IV (adult dose).

Text for Drug doses in children:

Hydrocortisone

> 12 years: 200 mg IM or IV slowly

6 to 12 years: 100 mg IM or IV slowly

6 months to 6 years: 50 mg IM or IV slowly

< 6 months: 25 mg IM or IV slowly

1. Text: Corticosteroids and antihistamines have a place as secondary treatment for anaphylaxis, and help to prevent oedema, cutaneous symptoms and relapse of the anaphylactic reaction, which can occur up to 24 h after the initial reaction.

Table 3. SSAI Guideline on treatment of anaphylactic reactions during anaesthesia:

Adults: Hydrocortisone 250 mg i.v. or Methylprednisolone 80 mg i.v.

Children: Hydrocortisone 50–100 mg i.v. or Methylprednisolone 2 mg/kg i.v.

1. D grade recommendation: Intravenous fluids (crystalloids) should be administered (boluses of 20 ml/kg) in patients experiencing cardiovascular instability.
2. D grade recommendation for Immediate management: Aggressive management of fluid resuscitation is a critical step in ensuring blood flow to vital organs. Repeated boluses of 20 ml/kg may be required.
3. Anaphylaxis treatment protocol (2015): Immediate measures: Intravenous fluids. Establish intravenous line for venous access and fluid replacement; keep open with 0.9 NL saline, push fluids for hypotension or failure to respond to epinephrine using 5-10 mg/kg as quickly as possible and up to 30 mL/kg in first hour for children and 1-2 L for adults
4. Text: During anaphylaxis, large volumes of fluids potentially leave the patient’s circulation and enter the interstitial tissue; therefore, rapid intravenous infusion of 0.9% saline (isotonic saline or normal saline) should be commenced as soon as the need for it is recognized (Table 5). The rate of administration should be titrated according to the blood pressure, cardiac rate and function, and urine output. All patients receiving such treatment should be monitored for volume overload.

Table 5. Basic Management of Anaphylaxis: Establish intravenous access using needles or catheters with wide-bore cannulae (14 or 16 gauge for adults). When indicated, give 1-2 litres of 0.9% (isotonic) saline rapidly. (eg. 5–10 mL/kg in the first 5–10 minutes to an adult; or 10 mL/kg to a child).

1. Text: Fluids (give as soon as available). If there is intravenous access, infuse intravenous fluids immediately. Give a rapid IV fluid challenge (20 mL/kg in a child or 500—1000 mL in an adult) and monitor the response; give further doses as necessary. There is no evidence to support the use of colloids over crystalloids in this setting. Consider colloid infusion as a cause in a patient receiving a colloid at the time of onset of an anaphylactic reaction and stop the infusion. Hartmann’s solution or 0.9% saline are suitable fluids for initial resuscitation. A large volume of fluid may be needed. If intravenous access is delayed or impossible, the intra-osseous route can be used for fluids or drugs when resuscitating children or adults, but only by healthcare workers who are accustomed to do so. Do not delay the administration of IM adrenaline attempting intra-osseous access.
2. Text: For severe anaphylactic reactions, the supply of large amounts of uid within a short time is necessary. This can only be achieved through large-bore venous access. If intravenous access is not possible, a special intra-osseous needle can be inserted preferably into the tibia. In case of anaphylactic shock, a supply of 0.5–1 liters, and possibly up to 2–3 liters of fluid – depending on the response – in a very short time is required for adults, for children initially 20 ml/kg body weight. Primarily, normal saline (NaCl 0.9 %) or balanced electrolyte solutions should be used. When large quantities of electrolyte solutions are given, they remain in the intravascular space for a short time only. Therefore, failing stabilization after the application of larger volumes of electrolytes (> 1 L) the additional application of colloid volume substitutes can be considered.
3. Text: Administer saline 0.9% or lactated Ringer’s solution at a high rate via an intravenous cannula of an appropriate gauge (large volumes may be required). "Text: The cornerstones of treatment are adrenaline and fluid therapy.
4. Table 3. SSAI Guideline on treatment of anaphylactic reactions during anaesthesia: NaCl 9 mg/ml, Ringer’s acetate or colloids. 20 ml/kg, more may be needed
5. D grade recommendation: Patients who presented with respiratory compromise should be closely monitored for at least 6–8 h, and patients who presented with circulatory instability require close monitoring for 12–24 h.
6. D grade recommendation for post crisis management: Most patients who have had a moderate to lifethreatening reaction will require admission to an intensive care unit/high dependency unit for around 24 hours. Where the reaction has been either minor or moderate and settled quickly with treatment, a minimum of six hours close monitoring is recommended
7. Strong Recommendation (2015): Individualize the duration of direct observation and monitoring after anaphylaxis but provide longer periods of observation for those patients with a history of risk factors for severe anaphylaxis (eg, asthma, previous biphasic reactions, or protracted anaphylaxis) for at least 4 to 8 hours.
8. Text: After apparent resolution of symptoms, duration of monitoring in a medically supervised setting should be individualized. For example, patients with moderate respiratory or cardiovascular compromise should be monitored for at least 4 hours, and if indicated, for 8–10 hours or longer, and patients with severe or protracted anaphylaxis might require monitoring and interventions for days.
9. Text: The observation of the anaphylaxis patient until he/she is in definite long-lasting remission is crucial.
10. Text: All anaphylactic drug reactions should be reported to the Medicines and Healthcare products Regulatory Agency (MHRA) using the Yellow Card scheme.
11. Text: Cases of anaphylaxis occurring during anaesthesia should be reported to the Medicines Control Agency and the AAGBI National Anaesthetic Anaphylaxis Database. Reports are more valuable if the diagnosis is recorded following specialist investigation of the reaction.
12. Text: Preventive strategies to avoid recurrence include allergen avoidance and allergen immunotherapy where possible should be implemented

A grade recommendation: The use of subcutaneous adrenaline alone as a premedication with snakebite antivenom reduces the risk of anaphylaxis to

the snake antivenom administration. The use of hydrocortisone alone does not reduce severe adverse reaction to snake antivenom. The routine use of prophylactic systemic premedication (H1- and/or H2-antihistamines or glucocorticoids) cannot be recommended in unselected people undergoing procedures with radiocontrast media as they do not prevent life-threatening reactions. There are no available data to support the use of premedication in patients with a previous reaction to another allergen.

1. Recommendation (2015): Individualize avoidance measures taking into consideration factors such as the patient’s age, activity, occupation, hobbies, residential conditions, access to medical care, and level of personal anxiety

Recommendation (2015): Use pharmacologic prophylaxis such as glucocorticosteroids and antihistamines in select situations (eg, to prevent recurrent anaphylactic reactions to RCM or to prevent idiopathic anaphylaxis).Recognize that pretreatment with antihistamines and corticosteroids may not prevent perioperative anaphylactic events.

Text (2015): Pharmacologic prophylaxis such as glucocorticoids and antihistamines can be used in select situations such as in the prevention of anaphylaxis to drugs or biologic agents (eg, radiocontrast material [RCM]) or to prevent recurrent episodes of idiopathic anaphylaxis. Prophylactic treatment is inconsistently effective and often fails to prevent events.

1. Text：Most recommendations for preventing recurrences of anaphylaxis, either by strict avoidance of the specific trigger(s) or relevant immunomodulation are based on expert opinion and consensus, rather than on rigorous, randomized, placebo-controlled, double-blind trials. An important exception to this statement is the use of subcutaneous immunotherapy with the relevant insect venom(s) to prevent recurrence of stinging insect anaphylaxis

Text: For patients at increased risk of anaphylaxis from RCM, a nonionic RCM should be administered and premedication with a corticosteroid and an antihistamine should be considered; however, use of premedication is controversial and does not prevent all future reactions.

1. Text: There is no evidence that pre-treatment with hydrocortisone or histamine-blocking drugs will reduce the severity of anaphylaxis.
2. Text: Pre-medication with antihistamines and steroids will probably not prevent anaphylactic shock but can reduce/prevent reactions caused by nonspecific histamine release. These reactions can also be prevented by avoiding histamine-liberating drugs altogether, or by injecting drugs slowly and one by one.
3. D grade recommendation: Training in the recognition and management of anaphylaxis, including the use of adrenaline auto-injectors, should be offered to all professionals dealing with patients at risk of anaphylaxis.

D grade recommendation: Training in the recognition and management of anaphylaxis should be offered to all patients and caregivers of children at risk of anaphylaxis ideally from the time of diagnosis. Training packages should be developed with the target groups. Training should cover allergen avoidance, symptoms of allergic reactions, when and how to use an adrenaline auto-injector, and what other measures are needed within the context of an anaphylaxis management plan. Training may involve more than one session to allow revision, an interactive scenario-based approach, a standardized program with manual and educational material and simulation tools. Content and language should be tailored to be understood and memorized.

1. Text: Prior to discharge from hospital, patients who have had a suspected anaphylaxis require a letter that contains a description of the reaction and the agents administered prior to the reaction. Referral to an anaesthetic allergy testing centre is required to investigate the reaction. The anaesthetic allergy testing centres in Australia, New Zealand, Hong Kong and Malaysia are listed on the ANZAAG website—www.anzaag.com
2. Anaphylaxis treatment protocol (2015): Discharge management: Education. Educate patient and family on how to recognize and how to treat anaphylaxis.
3. Anaphylaxis education should ideally begin before patients are discharged from the emergency department or other healthcare facility where their anaphylaxis was treated. Patients should be advised that they have experienced a potentially life-threatening medical emergency (“killer allergy”),and that if their symptoms recur within the next 72 hours, they should inject epinephrine and call emergency medical services or be taken to the nearest emergency facility by family or caregivers. They should also be advised that they are at increased risk for future episodes of anaphylaxis, and that they need follow-up, preferably assessment or reassessment by an allergy/immunology specialist. Medical identification (for example, bracelet or wallet card) stating their diagnosis of anaphylaxis, relevant concomitant diseases, and concurrent medications should be recommended. Anaphylaxis education should be personalized according to the needs of the individual patient, taking into consideration their age, concomitant diseases, concurrent medications, relevant anaphylaxis trigger(s), and likelihood of encountering such trigger(s) in the community.
4. Text: Patients need to know the allergen responsible and how to avoid it. Patients need to be able to recognise the early symptoms of anaphylaxis, so that they can summon help quickly and prepare to use their emergency medication. Patients and those close to them (e.g., family, friends, carers) should receive training in using the auto-injector and should practise regularly using a suitable training device, so that they will know what to do in an emergency.
5. Text: The patient should be trained in: the recognition of an anaphylactic reaction, symptom-orientated self-medication. correct positioning. making an emergency call.
6. Text: The patient should be warned against any substance which has tested positive (including skin testing, basophil allergen challenge tests, leucocyte histamine release test, flow cytometric analysis of in vitro-activated basophils, and drug provocation).

**Reference**

1. Li X, Cui C, Zheng H, et al. A Systematic Quality Appraisal of Anaphylaxis Diagnosis and Treatment Guidelines Using the AGREE Ⅱ Instrument[J]. Chin J Pharmacoepidemiol, 2020, 29(03): 336-341.
2. Guideline registration

The protocol was approved by the International Practice Guidelines Registry Platform (http://www.guidelines-registry.cn/) on August 2017 under registration ID IPGRP-2017CN026. The protocol describes:

- the formation of the project team;
- the scope of the guideline;
- the management of any conflict and funding interests;
- the formulation of clinical questions and outcomes;
- the synthesis and evaluation of the evidence;
- the assembly of the recommendations;
- the conduct of external reviews; and
- the plan for reporting, publication, and updating of the guideline.

The protocol has been published in the Chinese Journal of Pharmacoepidemiology^[1]^.

**Reference**

1. Li X, Zheng H, Men P, et al. Protocol of the Guideline for Emergency Management of Anaphylaxis[J]. Chin J Pharmacoepidemiol, 2020, 29(03):193-197.
2. Selection of Clinical Questions and Outcomes

The formulation of the clinical questions is the cornerstone of the guideline because it guides the direction^[1]^.We created a multidisciplinary team to pose the candidate questions, and then took a three-step Delphi approach^[2,3]^ to select questions, which ensured that we addressed the questions of highest concern to clinicians.

The selection and definition of clinical questions and outcomes were carried under the supervision of methodologists who took the following approach:

1. A draft list of clinical questions and outcomes was created and revised by the steering group and guideline development working group. The guideline expert advisory group then added clinical questions and suggested revisions.
2. A total of 34 clinical questions, and 6 outcomes were selected after thorough consultation.
3. At the first panel meeting on 29 August 2017, a Delphi method was conducted that included three rounds of inquiry. The active coefficient, coefficient of variation, and the frequencies of each score were calculated for quality control.
4. After the three rounds of the Delphi method, 28 pivotal clinical questions were selected that covered the diagnosis, preparation for the treatment, treatment, and administration after the treatment. Six important outcomes were also defined and included for the guideline. The remaining foreground questions (four essential and two important) were excluded from the guideline and saved for further revision or updates. Regarding the six outcomes, four were recognized as critical and the rest were recognized as important.
5. Experts contributing to the research reach and active coefficient of 100%, and the degree of consensus was good. The frequency of feedback scoring was ≥ 4 for all 28 foreground questions included and were greater than 75%. The result was achieved in the first round. Two outcomes: fatality rate and severity, each reached a higher degree of consensus with a coefficient of variation < 15%. More details on this process has been document in a manuscript that has been accepted by the Journal of Peking University (health science)^[4]^.
6. The steering group discussed the 28 pivotal clinical questions that were selected and decided to include 27 of them in the guideline. The question ‘how to perform desensitation therapy’ was deleted because it was outside the scope of emergency management.
7. When drafting recommendations, the steering group decided not to draft recommendation for “What are the key points for differential diagnosis of anaphylaxis?”. The reasoning was because no relevant original evidence and few related materials were found, and the experience lacked consensus.
8. For the question ‘What are the requirements for pre-hospital and in-hospital first-aid equipment?’, the panel reached consensus on recommendation as ‘Pre-hospital and in-hospital first aid equipment should contain enough adrenaline injection. (strong recommendation)’. Since this recommendation largely overlaps current recommendation 7.1
   (Epinephrine is the first-line medicine in GRADE II to IV patients. It can also be considered in GRADE I patients who have persistent gastrointestinal symptoms. [strong recommendation]), the panel decided to delete this clinical question and recommendation.

Ultimately, this guideline answered a total of 25 clinical questions. There was little evidence of economics for anaphylaxis, and the resource varied across China as well as the world. Hence, we only include questions that would be critical all around the world and did not select an economic outcome.

**Reference**

1. World Health Organization. WHO handbook for guideline development[M]. World Health Organization, 2014.
2. Dalkey N C . The Delphi method: an experimental study of group opinion[J]. Futures, 1969, 1(5):408-426. DOI:10.1016/S0016-3287(69)80025-X
3. Dalkey N, Helmer O. An experimental application of the Delphi method to the use of experts. Manag Sci. 1963;9(3):458–67. DOI:10.1287/mnsc.9.3.458.
4. Zheng H, Li X, Men P, et al. Selecting and defining the clinical questions and outcomes of guideline for the emergency treatment of anaphylaxis. Journal of Peking University (Health Sciences) ,2020,52(04):715-718.

## Table 1 The rating of the importance of outcomes

| Rating | Outcomes |
| --- | --- |
| Critical | Mortality  ICU admission^a^  Severity of anaphylaxis  Duration of symptoms  Cardiovascular adverse reaction (for safety of epinephrine)  Occurrence of anaphylaxis (for prevention)^a^ |
| Important | Hospital admission  Duration of hospital stay^a^  Further treatment^a^  Biphasic anaphylaxis or recurrence of anaphylaxis |
| Of little importance | Pharmacokinetics^a^  Inaccurate therapy^a^ |

^a^ This outcome was added through subsequent consensus by email.

1. External reviews

We conducted external reviews for ‘Guidelines for the Emergency Treatment of Anaphylaxis’ based on guidance from international guidelines development handbooks^[1-3]^ and from our previous experience^[4,5]^.

We conducted the external review for the 25 recommendations by questionnaire. Respondents were selected from six hospitals in five cities by convenience sampling. It incorporated 30 health care workers and 1 patient. The 30 medical workers comprised 18 doctors, 5 pharmacists and 5 nurses derived from 10 departments. It appraised the accuracy, clarity, feasibility of the guideline and respondents were encouraged to make suggestions in other aspects. The accuracy, clarity and feasibility of every recommendation were accepted by no less than 90% of respondents. The reviewers provided 72 comments regarding our recommendation.

In addition, the recommendations in this guideline were published on the (Chinese) Adverse Drug Reactions Journal for the public consultation^[6]^. Then we received feedback from 4 readers.

According to the feedback, two external review group leaders (Wang and Zhu) and the steering committee revised the recommendations. In the end, a total of 13 recommendations were modified.

## Table 1. Background of external reviewer

| Hosital | Doctor | Pharmacist | Nurses |
| --- | --- | --- | --- |
| Peking Union Medical College Hospital | 3 | 1 | 1 |
| Peking University Thirds Hospital | 3 | 1 | 1 |
| Shanghai Jiaotong University Medical School Ruijin Hospital | 3 | 1 | 1 |
| Hospital of Traditional Chinese Medicine of Zhongshan | 3 | 1 | 1 |
| Wuhan General Hospital of Guangzhou Military Command | 3 | 1 | 1 |
| Qilu Hospital of Shandong University | 3 | 1 | 1 |

## Table 2. Result of recognition in external review

|  | Accuracy | Clarity | Feasibility |
| --- | --- | --- | --- |
| Recommendation 1 | 100% | 97% | 100% |
| Recommendation 2 | 100% | 94% | 100% |
| Recommendation 3 | 97% | 97% | 97% |
| Recommendation 4 | 100% | 100% | 100% |
| Recommendation 5 | 97% | 97% | 97% |
| Recommendation 6.1 | 97% | 97% | 97% |
| Recommendation 6.2 | 97% | 97% | 97% |
| Recommendation 6.3 | 90% | 94% | 90% |
| Recommendation 6.4 | 100% | 100% | 100% |
| Recommendation 6.5 | 100% | 100% | 100% |
| Recommendation 6.6 | 100% | 100% | 100% |
| Recommendation 6.7 | 100% | 100% | 100% |
| Recommendation 6.8 | 93% | 94% | 94% |
| Recommendation 6.9 | 93% | 100% | 97% |
| Recommendation 6.10 | 90% | 97% | 94% |
| Recommendation 6.11 | 97% | 97% | 97% |
| Recommendation 6.12 | 97% | 97% | 97% |
| Recommendation 7 | 97% | 100% | 100% |
| Recommendation 8 | 100% | 100% | 100% |
| Recommendation 9 | 93% | 94% | 94% |
| Recommendation 10 | 97% | 100% | 100% |
| Recommendation 11 | 90% | 97% | 94% |
| Recommendation 12 | 100% | 100% | 100% |
| Recommendation 13 | 97% | 100% | 97% |
| Recommendation 14 | 97% | 97% | 97% |

**Reference**

1. World Health Organization. WHO handbook for guideline development[M]. World Health Organization, 2014.
2. Institute of Medicine (US) Committee on Standards for Developing Trustworthy Clinical Practice Guidelines; Graham R, Mancher M, Miller Wolman D, et al., editors. Clinical Practice Guidelines We Can Trust. 2011 Mar. Trustworthy Clinical Practice Guidelines: Challenges and Potential [Internet]. [cited 2020 Aug 11] Available from: https://www.ncbi.nlm.nih.gov/books/NBK209532/
3. Brouwers M C, Kho M E, Browman G P, et al. AGREE II: advancing guideline development, reporting and evaluation in health care[J]. CMAJ 2010;182:E839-42. DOI:10.1503/cmaj.090449. 20603348
4. Chen K, Song Z, Liu W, et al. Chinese Practice Guideline for Individualized Medication of Voriconazole: Recommendations External Review[J]. Chin J Pharmacoepidemiol, 2017, 26(02):143-148.
5. Zhou P, Chen Y, Xu B, et al. Rapid advice Guideline for Azithromycin for Injection in Pediatrics: recommendations external review. Chin Hosp Pharm J, 2018,38(17):1773-1776.
6. Li X, Zhai S, Wang Q, et al. Recommendation in Guideline for the Emergency Management[J]. Adverse Drug Reactions Journal,2019(02):85-91.
